# IRF7 drives macrophages to kill bacteria and improves septic outcomes via autophagy

**DOI:** 10.1172/jci.insight.189420

**Published:** 2025-11-10

**Authors:** Guiming Chen, Kangxin Li, Haihua Luo, Lianxu Zhao, Yong Jiang

**Affiliations:** 1 Department of Neurology, Shenzhen Hospital, Southern Medical University, Shenzhen, Guangdong, China.; 2 Guangdong Provincial Key Laboratory of Proteomics, State Key Laboratory of Organ Failure Research, School of Basic Medical Sciences, Southern Medical University, Guangzhou, Guangdong, China.; 3 Department of Respiratory and Critical Care Medicine, The Tenth Affiliated Hospital (Dongguan People’s Hospital), Southern Medical University, Dongguan, Guangdong, China.; 4 State Key Laboratory of Metabolic Dysregulation & Prevention and Treatment of Esophageal Cancer and; 5 Henan Key Laboratory of Critical Care Medicine, Department of Emergency Medicine, The First Affiliated Hospital, Zhengzhou University, Zhengzhou, Henan, China.; 6 Institute of Infection and Immunity, Henan Academy of Innovations in Medical Science, Zhengzhou, Henan, China.

**Keywords:** Infectious disease, Therapeutics, Autophagy, Bacterial infections, Macrophages

## Abstract

Sepsis contributes substantially to mortality rates worldwide, yet clinical trials that have focused on its underlying pathogenesis have failed to demonstrate benefits. Recently, enhancing self-defense has been regarded as an emerging therapeutic approach. Autophagy is a self-defense mechanism that protects septic mice, but its regulatory factor is still unknown. Moreover, the role of interferon regulatory factor 7 (IRF7) in sepsis has been debated. Here, we showed that *Irf7* deficiency increased mortality during polymicrobial sepsis. Furthermore, IRF7 drove macrophages to protect against sepsis. Mechanistically, IRF7 is a transcription factor that upregulates the expression of autophagy-related genes responsible for autophagosome formation and autolysosome maturation, induces autophagic killing of bacteria, and ultimately reduces septic organ injury. Recombinant adeno-associated virus 9–*Irf7*–mediated IRF7 overexpression promoted the autophagic clearance of pathogens and improved sepsis outcomes, which may be the mechanism underlying the observed improvement in bacterial clearance. These findings provide evidence that IRF7 is the underlying regulatory factor that drives autophagy to eliminate pathogens in macrophages during sepsis. Collectively, IRF7 overexpression represents a potential host-directed therapeutic strategy for preclinical sepsis models, operating independently of antibiotic mechanisms.

## Abstract 2

IRF7 drives autophagy in macrophages to eliminate pathogens and finally improve sepsis outcome, suggesting therapeutic potential.

## Graphical abstract

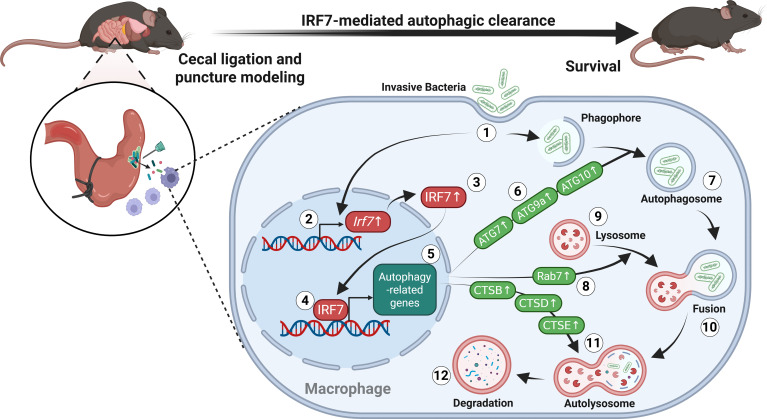

## Introduction

Sepsis is life-threatening organ dysfunction because of a dysregulated host response to infection (1). Owing to its complexity and elusiveness, sepsis is a major cause of death in intensive care units; thus, the pathogenesis of sepsis has been a common topic of research in recent decades (2). Unfortunately, clinical trials based on the pathogenesis of sepsis have failed to demonstrate benefits (3, 4). Therefore, more studies have focused on the mechanisms of self-defense during sepsis in recent years (5, 6).

Bacterial clearance, which is performed mainly by macrophages, plays an important role in the development of sepsis and sepsis-induced organ injury (7). Research has revealed that autophagy is induced during septic insult and that triggering autophagy increases the bacterial killing efficiency of macrophages to decrease the bacterial load, thus limiting organ injury and death in septic mice (6, 8). However, much remains to be learned regarding which endogenous factors regulate autophagy in sepsis. Autophagy consists of several sequential steps — initial activation, phagophore elongation, autophagosome formation, and autolysosome maturation — which are controlled by autophagy-related genes (ATGs) (9). It is clear that transcription factors determine the ATG repertoire (10–12).

Interferon regulatory factors (IRFs) are pivotal transcription factors controlling innate immunity, primarily recognized for directing type I interferon (IFN) transcription during infections through central regulators IRF3 and IRF7 (13). During viral infections, IRF7 amplifies IRF3-induced type I IFN production via a positive feedback loop wherein IFN signaling further activates IRF7 (14). However, recent studies reveal that IRFs critically drive nonviral infections through IFN-independent mechanisms, exemplified by IRF8 coordinating pan-autophagic gene expression to clear *Listeria monocytogenes* (11) and IRF1 suppressing autophagy to potentiate apoptosis in endotoxin-challenged macrophages (15), establishing IRFs as master regulators of infection-associated macrophage autophagy. Crucially, our preliminary data revealed divergent temporal dynamics: IRF1 peaked in macrophages at 2 hours (early phase), while IRF7 progressively increased from 8 to 24 hours (middle-late phase), with other IRFs remaining at baseline (Supplemental Figure 1, A and B; supplemental material available online with this article; https://doi.org/10.1172/jci.insight.189420DS1). Given that IRF7’s upregulation pattern precisely coincides with the autophagic flux window during the middle-late stage of sepsis (6), we hypothesized that IRF7 programs autophagy to control disease outcomes. Notably, IRF7’s functional role in sepsis remains unresolved, with reports indicating protection (16, 17) or detriment (18). Consequently, this study delineates IRF7’s functional role and underlying molecular mechanisms using a clinically relevant murine sepsis model and complementary in vitro assays.

## Results

### Irf7-deficient mice are more susceptible to polymicrobial sepsis.

**Figure 1:**
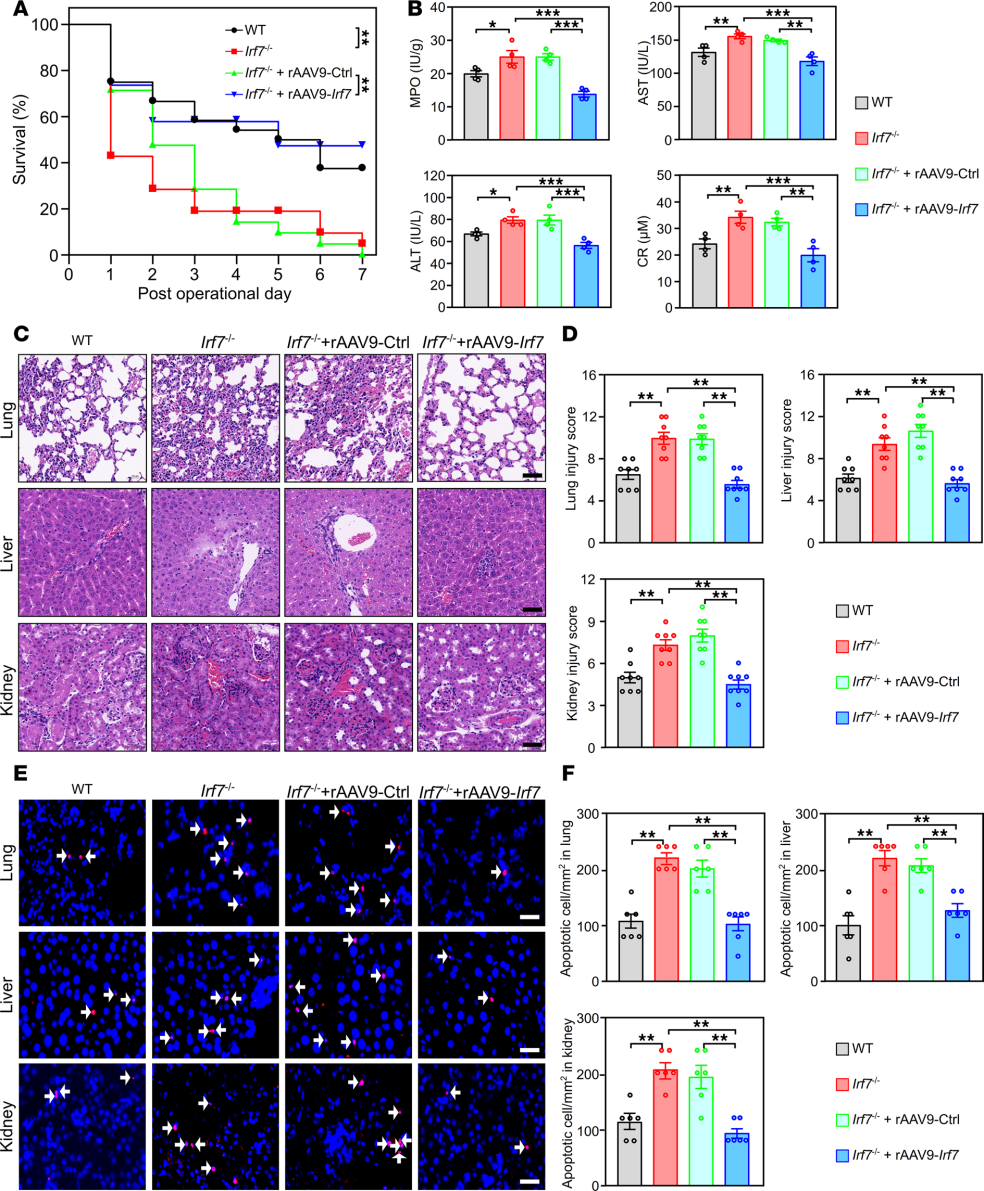
IRF7 is required for defense against polymicrobial sepsis. (**A**) Effect of interferon regulatory factor 7 (*Irf7*) gene deficiency on the survival of mice subjected to CLP modeling. Statistical analysis of log rank test was performed on Kaplan-Meier survival curves. rAAV9-Ctrl or rAAV9-*Irf7*, 1 × 10^11^ vector genomes (vg)/mouse. WT group, *n* = 24; *Irf7*^–/–^ group, *n* = 21; *Irf7*^–/–^ + rAAV9-Ctrl group, *n* = 21; and *Irf7*^–/–^ + rAAV9-*Irf7* group, *n* = 19. (**B**) Myeloperoxidase (MPO) activity in lung and plasma aspartate aminotransferase (AST), alanine aminotransferase (ALT), and creatinine (CR) levels. Data represent the mean ± SEM. *P < 0.05, **P < 0.01, ***P < 0.001, 1-way ANOVA with Bonferroni’s correction. (**C** and **D**) Tissue injury and inflammation of mice that underwent CLP were measured by H&E staining (**C**), and the corresponding histological scores of lung, liver, and kidney were respectively shown in **D**. Scale bar, 50 μm. Data represent the mean ± SEM. **P < 0.01, 1-way ANOVA with Bonferroni’s correction. (**E** and **F**) The representative pictures of TUNEL staining (**E**) and the corresponding apoptotic cell quantification of (**F**) lung, liver, and kidney. White arrow, TUNEL-positive cells. Scale bar, 25 μm. Error bars, ± SEM. **P < 0.01, 1-way ANOVA with Bonferroni’s correction.

To determine whether IRF7 is associated with the pathogenesis of sepsis, we first monitored the 7-day survival rates of wild-type (WT) and *Irf7*^–/–^ mice after cecal ligation and puncture (CLP), a gold standard model of sepsis (19). As shown in Figure 1A, *Irf7* deficiency decreased the survival rate of the mice subjected to CLP, but injection with recombinant adeno-associated virus 9–*Irf7* (rAAV9-*Irf7*) increased the survival rate of these *Irf7*^–/–^ mice, which was close to WT mice that underwent CLP. Moreover, rAAV9-Ctrl did not affect the survival rate of the *Irf7*^–/–^ mice. Additionally, the levels of myeloperoxidase (MPO), aspartate aminotransferase (AST), alanine aminotransferase (ALT), and creatinine (CR) in *Irf7*^–/–^ mice were markedly higher than those in WT mice after CLP, but these values in *Irf7*^–/–^ and rAAV9-*Irf7* mice decreased and were similar to those in WT mice (Figure 1B). Histological analysis revealed that the lungs, livers, and kidneys of *Irf7*^–/–^ mice presented more pathological changes than the WT mice did, and rAAV9-*Irf7* reduced these pathological changes in *Irf7*^–/–^ mice (Figure 1, C and D). Consistent with these findings, *Irf7* deficiency markedly increased the number of apoptotic cells in the lungs, livers, and kidneys following CLP, while rAAV9-*Irf7* reduced the number of apoptotic cells in the lungs, livers, and kidneys of *Irf7*^–/–^ mice that underwent CLP (Figure 1, E and F). Moreover, cytokine profiling revealed time-dependent dysregulation. *Irf7*^–/–^ mice showed markedly elevated serum TNF-α, IL-1β, or IL-6 at 16 hours, followed by IL-12 elevation specifically at 24 hours, despite comparable levels of all cytokines at earlier time points (0–12 hours). Crucially, rAAV9-*Irf*7 administration partially normalized these hyperinflammatory cytokines to WT CLP baselines, demonstrating IRF7’s essential role in restraining late-phase hyperinflammation (Supplemental Figure 2A). Taken together, these results clearly demonstrated that IRF7 was required for defense against polymicrobial sepsis in mice.

### IRF7-dependent macrophages protect mice from sepsis.

**Figure 2:**
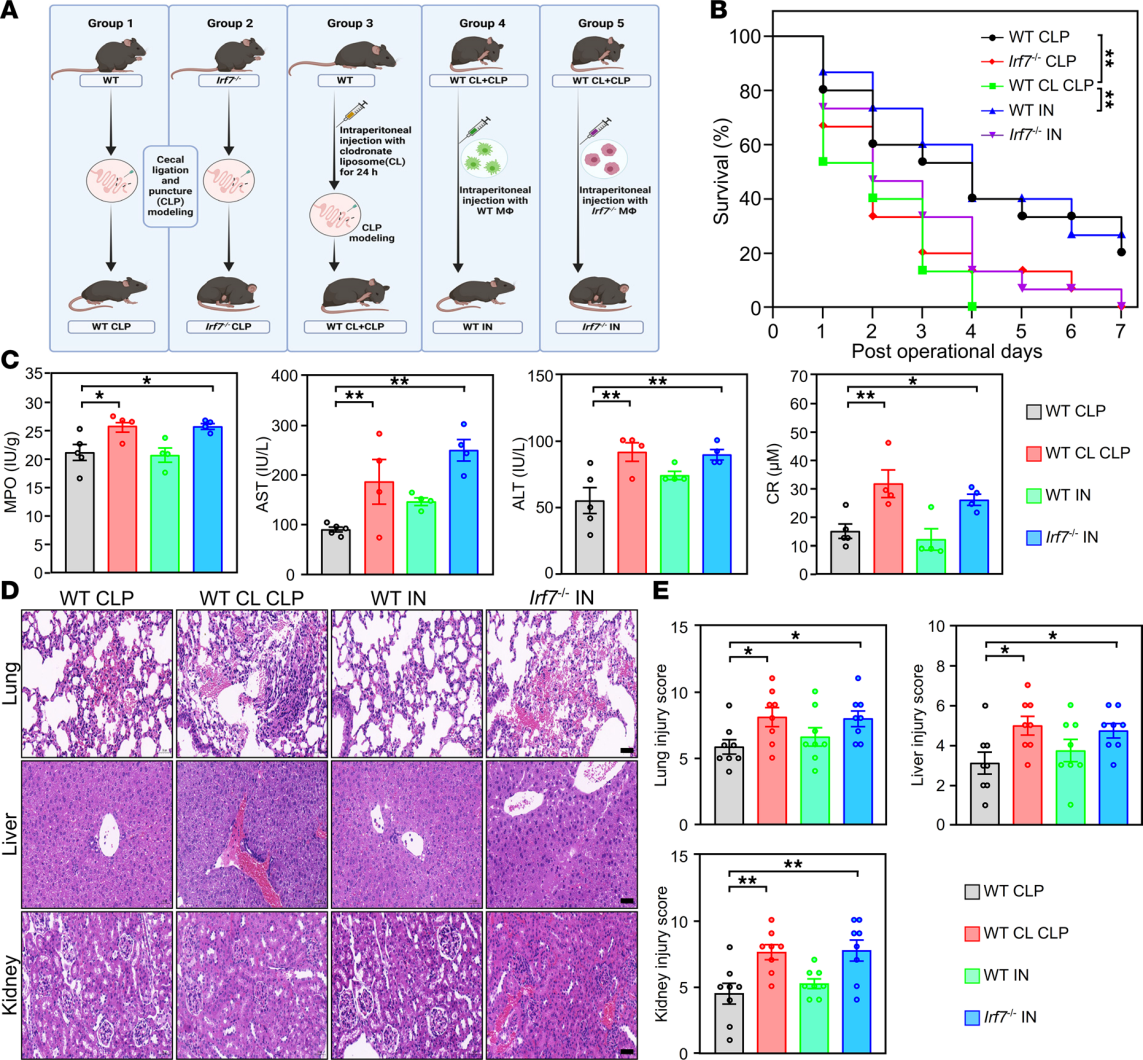
IRF7 drives macrophages to protect against sepsis. (**A**) Schematic of the treatment for the different groups of mice. WT CLP, WT mice underwent CLP. *Irf7*^–/–^ CLP, *Irf7*^–/–^ mice underwent CLP. WT CL CLP, WT mice pretreated with clodronate liposome (CL) for depletion of local macrophages that underwent CLP. WT IN, recipient WT CL mice were intraperitoneally injected with WT peritoneal macrophages 1 hour after CLP. *Irf7*^–/–^ IN, recipient WT CL mice were intraperitoneally injected with *Irf7*^–/–^ peritoneal macrophages 1 hour after CLP. (**B**) Survival rates of mice were determined until 7 days after CLP (*n* = 15, log rank test). **P < 0.01. (**C**) MPO activity in lung and plasma AST, ALT, CR levels. Data represent the mean ± SEM. *P < 0.05, **P < 0.01, 1-way ANOVA with Bonferroni’s correction. (**D** and **E**) Tissue injury and inflammation of mice that underwent CLP were measured by H&E staining (**D**), and the corresponding histological scores of lung, liver, and kidney were respectively shown in **E**. Scale bar, 50 μm. Error bars, ± SEM. *P < 0.05, **P < 0.01, 1-way ANOVA with Bonferroni’s correction.

Macrophages, T cells, and NK cells contribute to sepsis pathogenesis (20–22). We found that IRF7 protein was exclusively upregulated in macrophages (peak 16 hours post-CLP), with baseline expression in T cells and NK cells across all time points (Supplemental Figure 3, A and B). In addition, *Irf7* deficiency reduced survival in septic mice, while T cell depletion further decreased survival in *Irf7*^–/–^ CLP mice, suggesting that T cells provide another protective mechanism distinct from IRF7-mediated defense (Supplemental Figure 3C). Conversely, NK cell depletion increased the final number of survivors in both WT and *Irf7*^–/–^ mice (WT NK1.1 Ab vs. WT, 12 vs. 7; Irf7^–/–^ NK1.1 Ab vs. Irf7^–/–^, 6 vs. 0), indicating that NK cells do not mediate IRF7-dependent protection (Supplemental Figure 3C). Critically, macrophage depletion reduced survival in WT CLP mice, phenocopying the survival reduction caused by *Irf7* deficiency. Concurrently, macrophage depletion failed to alter survival outcomes in septic *Irf7*^–/–^ mice (Supplemental Figure 3C), indicating the detrimental effect of *Irf7* deficiency is largely mediated through impaired macrophage function. To further validate macrophage-intrinsic IRF7 dependency, WT and *Irf7*^–/–^ peritoneal macrophages isolated from WT and *Irf7*^–/–^ mice, respectively, were intraperitoneally infused into WT CLP mice pretreated with clodronate liposomes (WT CL CLP mice) to deplete the local macrophages, hereafter referred to as WT IN and *Irf7*^–/–^ IN mice (Figure 2A). As shown in Figure 2B, the survival rate of the WT CL CLP mice was as low as that of the *Irf7*^–/–^ CLP mice, but the survival rate increased significantly in WT IN but not *Irf7*^–/–^ IN mice, the latter of which had a survival rate similar to that of WT CLP mice. Additionally, the MPO, AST, ALT, and CR levels in WT CL CLP mice (Figure 2C) and the degree of organ injury (Figure 2, D and E) in WT CL CLP mice were reduced by WT IN but not *Irf7*^–/–^ IN. In sum, these results suggest that IRF7 drives macrophages to protect against sepsis.

### IRF7 is required for macrophage-mediated bacterial killing during sepsis.

**Figure 3:**
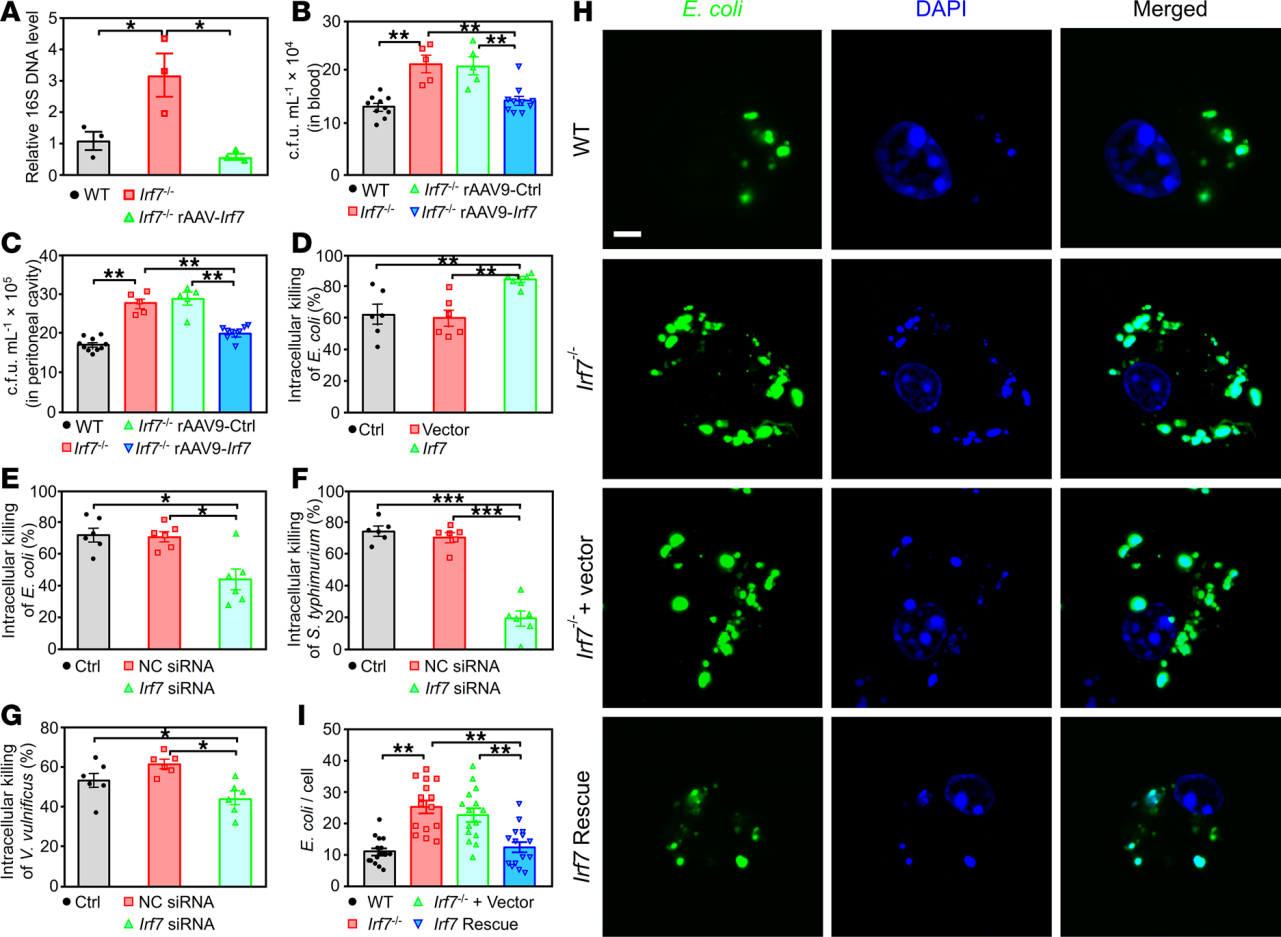
IRF7 promotes bacterial killing of macrophages in sepsis. (**A**) Relative bacteria DNA in blood from the indicated CLP mice was determined by quantitative PCR assay. Data represent the mean ± SEM. *P < 0.05, 1-way ANOVA with Bonferroni’s correction. (**B** and **C**) Bacterial loads in the blood (**B**) and peritoneal cavity (**C**) were determined by CFU assay. Data represent the mean ± SEM. **P < 0.01, 1-way ANOVA with Bonferroni’s correction. (**D**) Intracellular killing of *E*. *coli* by WT BMDMs transfected with either empty vector or *Irf7* gene. Data represent the mean ± SEM. **P < 0.01, 1-way ANOVA with Bonferroni’s correction. (**E**–**G**) Intracellular killing of *E*. *coli* (**E**), *S*. *typhimurium* (**F**), and *V*. *vulnificus* (**G**) by WT BMDMs transfected with either negative control (NC) siRNA or *Irf7* siRNA. Data represent the mean ± SEM. *P < 0.05, ***P < 0.001, 1-way ANOVA with Bonferroni’s correction. (**H** and **I**) BMDMs of WT, *Irf7*^–/–^, *Irf7*^–/–^ + Vector, and *Irf7* Rescue groups were infected with GFP–*E*. *coli* at MOI 50 for 45 minutes followed by adding 100 μg/mL gentamicin to kill extracellular bacteria for 1 hour. After 1 hour of further cultivation, cells were washed with PBS for 3 times and fixed with 4% paraformaldehyde, then stained with DAPI. (**H**) Representative confocal images. Scale bar, 5 μm. (**I**) Intracellular *E*. *coli* number per cell with SEM. **P < 0.01, 1-way ANOVA with Bonferroni’s correction.

Bacterial clearance, which is mainly carried out by macrophages, plays important roles in both the development of sepsis and sepsis-induced organ injury (7). Our data revealed that rAAV9-*Irf7* reduced the amount of bacterial DNA in the blood of *Irf7*^–/–^ mice after CLP (Figure 3A). Additionally, compared with those in WT mice, the bacterial loads in both the blood and peritoneal cavities were markedly elevated in *Irf7*^–/–^ mice after CLP, but reduced in *Irf7*^–/–^ + rAAV9-*Irf7* mice, reaching a level similar to that in WT mice (Figure 3, B and C). However, the total number of bacteria in the ligated cecum did not significantly differ among the WT, *Irf7*^–/–^, *Irf7*^–/–^ + rAAV9-Ctrl, and *Irf7*^-–/–^ + rAAV9-*Irf7* mice before cecal puncture (Supplemental Figure 4A). Thus, we next focused on determining if IRF7 is required for bacterial clearance during sepsis. The *Irf7* gene did not affect the phagocytic capacity of bone marrow–derived macrophages (BMDMs) (Supplemental Figure 4B) but did enhance the intracellular killing of *E*. *coli* (Figure 3D). Furthermore, *Irf7* siRNA significantly decreased the intracellular killing of *E*. *coli* (Figure 3E), *Salmonella* *typhimurium* (Figure 3F), and *Vibrio* *vulnificus* (Figure 3G) in macrophages. Next, BMDMs were incubated with GFP–*E*. *coli* for 45 minutes, after which the labeled bacteria were cleared over 2 hours. The immunofluorescence staining results revealed that the bacterial counts in the *Irf7*^–/–^ BMDMs were substantially greater than those in WT cells but were reduced by *Irf7* gene (Figure 3, H and I). Taken together, these results suggest that IRF7 promotes bacterial killing during polymicrobial sepsis.

### IRF7 is involved in the autophagic response of macrophages during sepsis.

**Figure 4:**
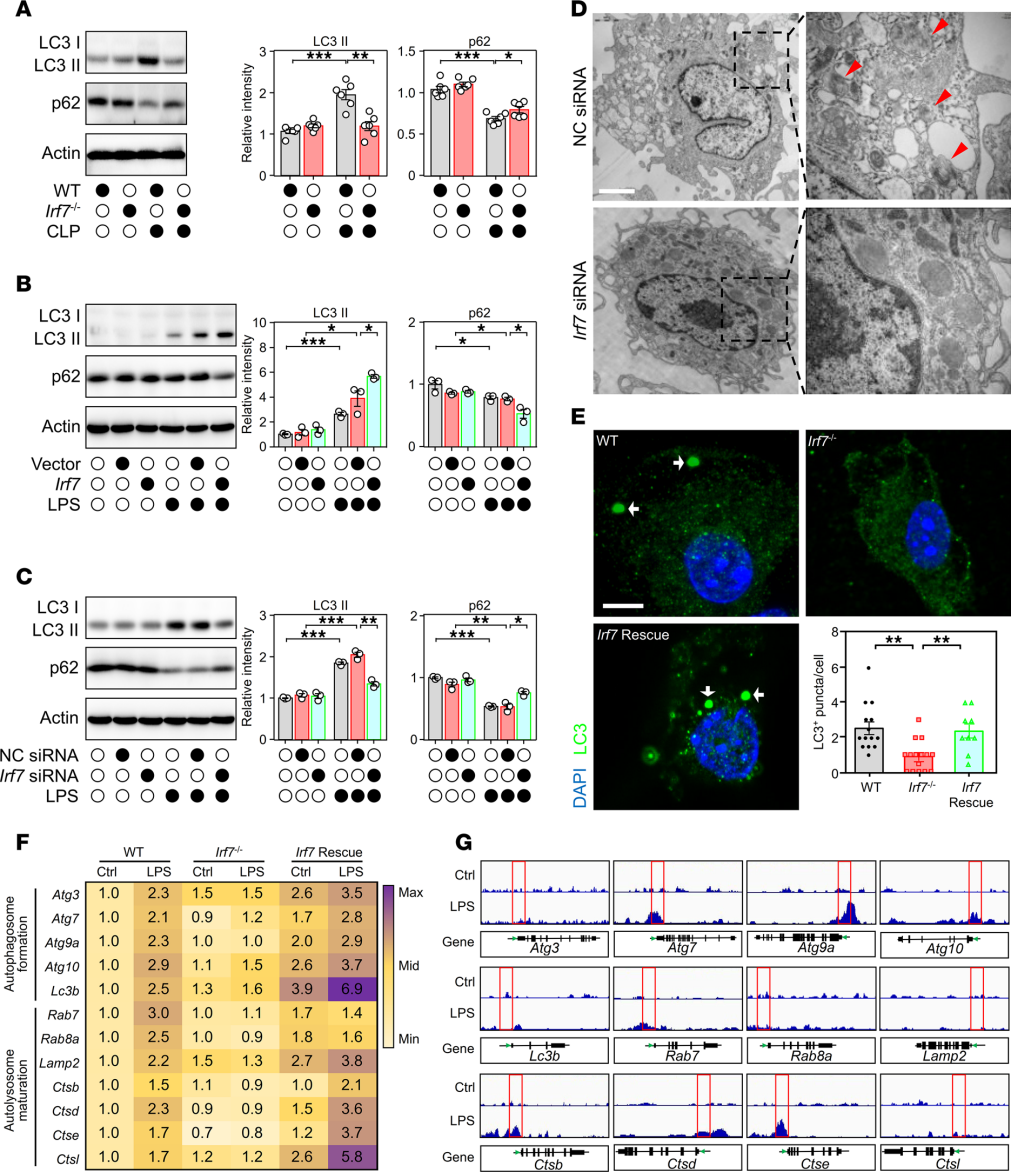
IRF7 promotes the autophagic response of macrophages during sepsis. (**A**) Immunoblot analysis of LC3 and p62 in protein extracts from peritoneal macrophages from WT and *Irf7*^–/–^ mice that underwent CLP for 16 hours. The protein intensities were normalized to Actin. Data represent the mean ± SEM. *P < 0.05, **P < 0.01, ***P < 0.001, 1-way ANOVA with Bonferroni’s correction. (**B** and **C**) After treatment with LPS for 16 hours, representative immunoblot detecting LC3 and p62 in cellular lysates isolated from BMDMs transfected with (**B**) either empty vector or *Irf7* gene or (**C**) either NC siRNA or *Irf7* siRNA. Data represent the mean ± SEM. *P < 0.05, **P < 0.01, 1-way ANOVA with Bonferroni’s correction. (**D**) Transmission electron microscopy (TEM) images revealed the ultrastructure of fixed BMDMs in different groups. Red triangles mark autophagosomes surrounded by dual/multi-layer membrane. Scale bar, 2 μm. (**E**) Representative confocal images of WT, *Irf7*^–/–^, and *Irf7* Rescue BMDMs treated with LPS for 16 hours. White arrow, LC3-positive puncta (autophagosome). Lower right panel: Scatterplot shown as the LC3-positive puncta number per cell with SEM. **P < 0.01, 1-way ANOVA with Bonferroni’s correction. Scale bar, 5 μm. (**F**) Heatmaps of reverse transcription quantitative PCR data for ATGs of WT, *Irf7*^–/–^, and *Irf7* Rescue BMDMs administrated without (Ctrl) or with LPS. Numerals and color scale indicate fold-change over WT Ctrl group. (**G**) ChIP-Seq signal intensities of selected ATGs were represented by sequencing. Red boxes, transcription start regions. *Cts*, cathepsin gene.

Next, the potential mechanism by which IRF7 mediates its protective effects against polymicrobial sepsis was investigated. We observed that the level of microtubule-associated protein 1 light chain 3 (LC3), a component of the autophagosome membrane, was decreased in *Irf7*^–/–^ mice compared with WT mice after CLP, and the level of p62, which is degraded during the autophagy process, was increased in *Irf7*-deficient mice (Figure 4A). In vitro, LPS-challenged BMDMs demonstrated that *Irf7* overexpression not only upregulated LC3 protein levels and accelerated p62 protein degradation (Figure 4B) but also coordinately induced key autophagy regulators across initiation (Unc-51 like autophagy activating kinase 1, ULK1), nucleation (Beclin1), elongation (ATG7), maturation and fusion (Ras-related protein Rab-7, RAB7; lysosomal-associated membrane protein 2, LAMP2), and mitochondrial autophagy (dynamin-related protein 1; DRP1) (Supplemental Figure 5A). Conversely, *Irf7* siRNA significantly disrupted this molecular cascade, altering expression of all 8 effectors (Figure 4C and Supplemental Figure 5B). Additionally, ultrastructural analysis revealed that interfering with *Irf7* expression markedly decreased the number of autophagosomes in LPS-treated macrophages (Figure 4D). The results of immunofluorescence staining revealed that autophagosomes were absent in *Irf7*^–/–^ BMDMs treated with LPS, but autophagosomes reappeared in the *Irf7* rescue group (Figure 4E). In sum, these results suggest that IRF7 is a regulator of autophagy in macrophages during sepsis.

IRF7 is a well-known transcription factor (23). Here, we observed that IRF7 translocated from cytoplasm into nuclei of BMDMs after LPS treatment (Supplemental Figure 6A). Next, mRNA analysis verified reduced expression of 12 ATGs in LPS-treated *Irf7*^–/–^ BMDMs versus LPS-treated WT cells, with full restoration upon IRF7 reconstitution (Figure 4F). We next performed chromatin immunoprecipitation sequencing (ChIP-Seq) in LPS-treated versus untreated BMDMs. Figure 4G displays IRF7 binding signals for these 12 ATGs (Figure 4F) exhibiting IRF7-dependent expression shifts upon LPS stimulation, revealing direct promoter occupancy for 7 targets (*Atg7*, *Atg9a*, *Atg10*, *Rab7*, *Ctsb*, *Ctsd*, and *Ctse*) but no enrichment for 5 genes (*Atg3*, *Lc3b*, *Rab8a*, *Lamp2*, *Ctsl*). These data indicated that IRF7 orchestrates autophagy through both direct promoter binding and indirect coordination of downstream effectors. Kyoto Encyclopedia of Genes and Genomes (KEGG) pathway enrichment analysis of IRF7-bound targets further revealed marked enrichment in cellular pathways critical for intracellular bacterial clearance, specifically endocytosis, lysosomal processing, and autophagy (Supplemental Figure 6B). These integrated findings collectively establish IRF7 as a master transcriptional regulator of sepsis-induced autophagy.

### IRF7 is required for the autophagic bacterial killing.

**Figure 5:**
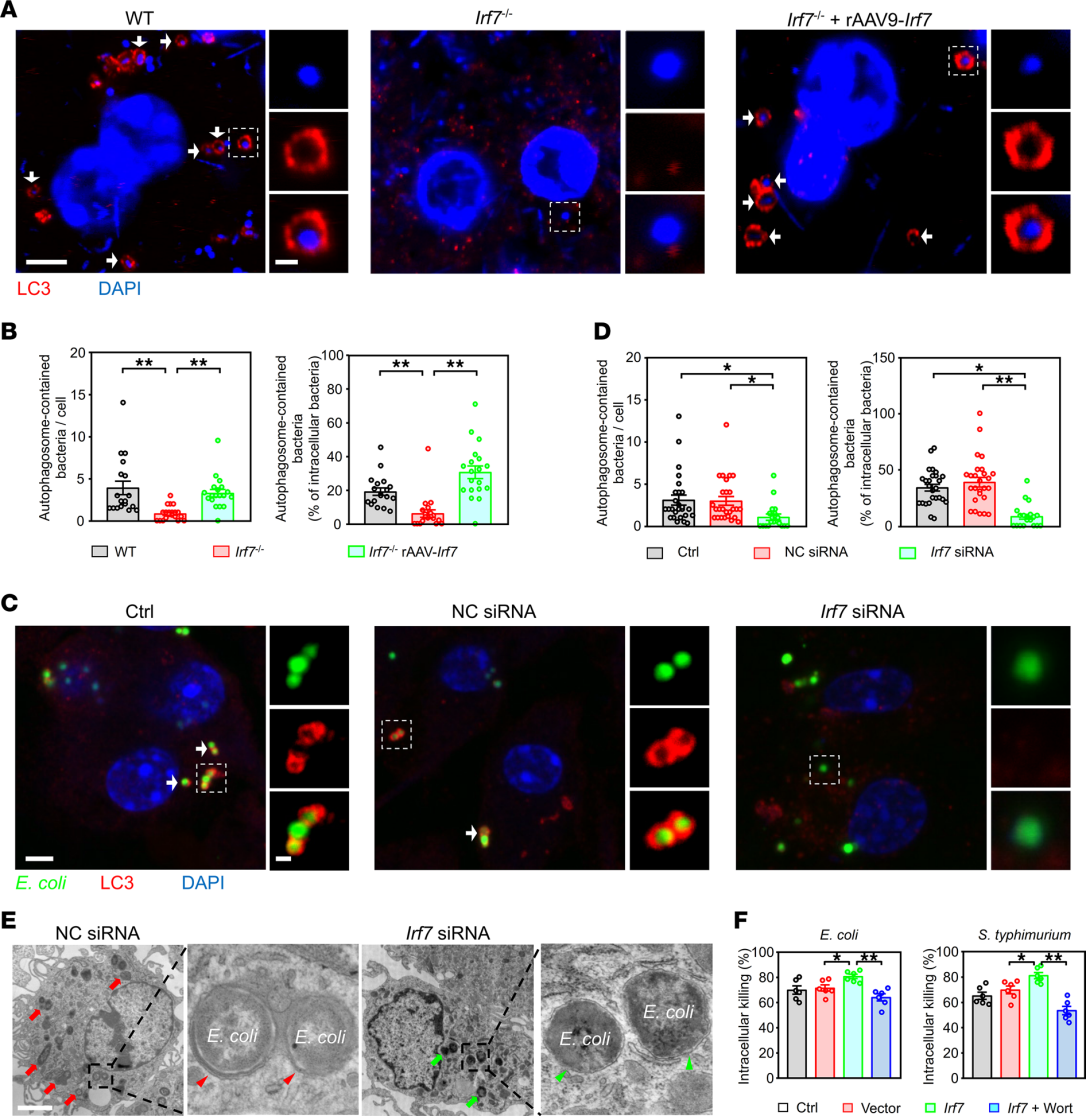
IRF7 triggers macrophages to kill bacteria via autophagy. (**A** and **B**) Analysis of autophagosomes containing bacteria in peritoneal macrophages of WT, *Irf7*^–/–^, and *Irf7* + rAAV-*Irf7* mice that underwent CLP for 16 hours. rAAV9-*Irf7*, 1 × 10^11^ vg/mouse. (**A**) Representative fluorescent pictures. White arrow, LC3-positive vesicles (autophagosomes) packed with smaller DAPI-positive dots (bacteria). Scale bars (main image, 5 μm; inset image, 1 μm). (**B**) Scatterplot with bar depicts the number of bacteria surrounded with autophagosomes per cell (left panel) and the percentage of bacteria surrounded with autophagosomes of the intracellular bacteria (right panel). Error bars, ± SEM. **P < 0.01, 1-way ANOVA with Bonferroni’s correction. (**C** and **D**) Analysis of autophagosomes containing *E*. *coli* in BMDMs. IRF7 in BMDMs was silenced by *Irf7* siRNA. Then, the BMDMs from the indicated groups were incubated with *E*. *coli* for 2 hours. (**C**) Representative fluorescence pictures. White arrow, LC3-positive vesicles packed with GFP-positive dots (*E*. *coli*). Scale bars (main image, 5 μm; inset image, 1 μm). (**D**) Scatterplot with bar depicts the number of bacteria surrounded with autophagosomes per cell (left panel) and percentage of bacteria surrounded with autophagosomes of the intracellular bacteria (right panel). Error bars, ± SEM. *P < 0.05, **P < 0.01, 1-way ANOVA with Bonferroni’s correction. (**E**) Representative TEM images of BMDMs treated with *E*. *coli*. Red arrows represent autophagosome-contained bacteria. Red arrowhead indicates the double membrane, a classic feature of autophagosome. Green arrows, no- or single-membrane-encapsulated bacteria. Green arrowhead, single membrane. Scale bar, 2 μm. (**F**) Intracellular killing of *E*. *coli* and *S*. *typhimurium* by BMDMs. Wortmannin (Wort) was pre-added where indicated (100 nM for 4 hours). *P < 0.05, **P < 0.01.

Drugs that induce (Tat-Beclin1; BECN1) or inhibit (wortmannin; Wort) autophagy decreased and increased bacterial loads, respectively, in WT mice 16 hours after CLP (Supplemental Figure 7A), suggesting that autophagy is the critical mechanism by which pathogens are eliminated during sepsis. Next, we isolated peritoneal macrophages from mice after CLP. Fewer intracellular bacteria were contained in the autophagosomes in *Irf7*^–/–^ macrophages while the number of bacteria in autophagosomes was restored in macrophages from *Irf7*^–/–^ + rAAV9-*Irf7* mice (Figure 5, A and B). In vitro, overexpression or knockdown of IRF7, achieved using *Irf7* gene and *Irf7* siRNA, respectively, was confirmed by Western blot (Supplemental Figure 8). *Irf7* siRNA dramatically reduced the numbers of LC3-positive vesicles enclosing *E*. *coli* (Figure 5, C and D). Correspondingly, *E*. *coli* in WT BMDMs transfected with *Irf7* siRNA were rarely encapsulated within double-membrane autophagosomes (Figure 5E). To further verify that IRF7 is associated with autophagy-mediated bacterial killing, we pretreated *Irf7*-transfected macrophages with Wort. As shown in Figure 5F, Wort markedly inhibited bacterial killing augmented by *Irf7* in BMDMs. Collectively, our data revealed that IRF7 promoted bacterial killing via autophagy in macrophages during sepsis.

### IRF7 defends against polymicrobial sepsis via autophagy.

**Figure 6:**
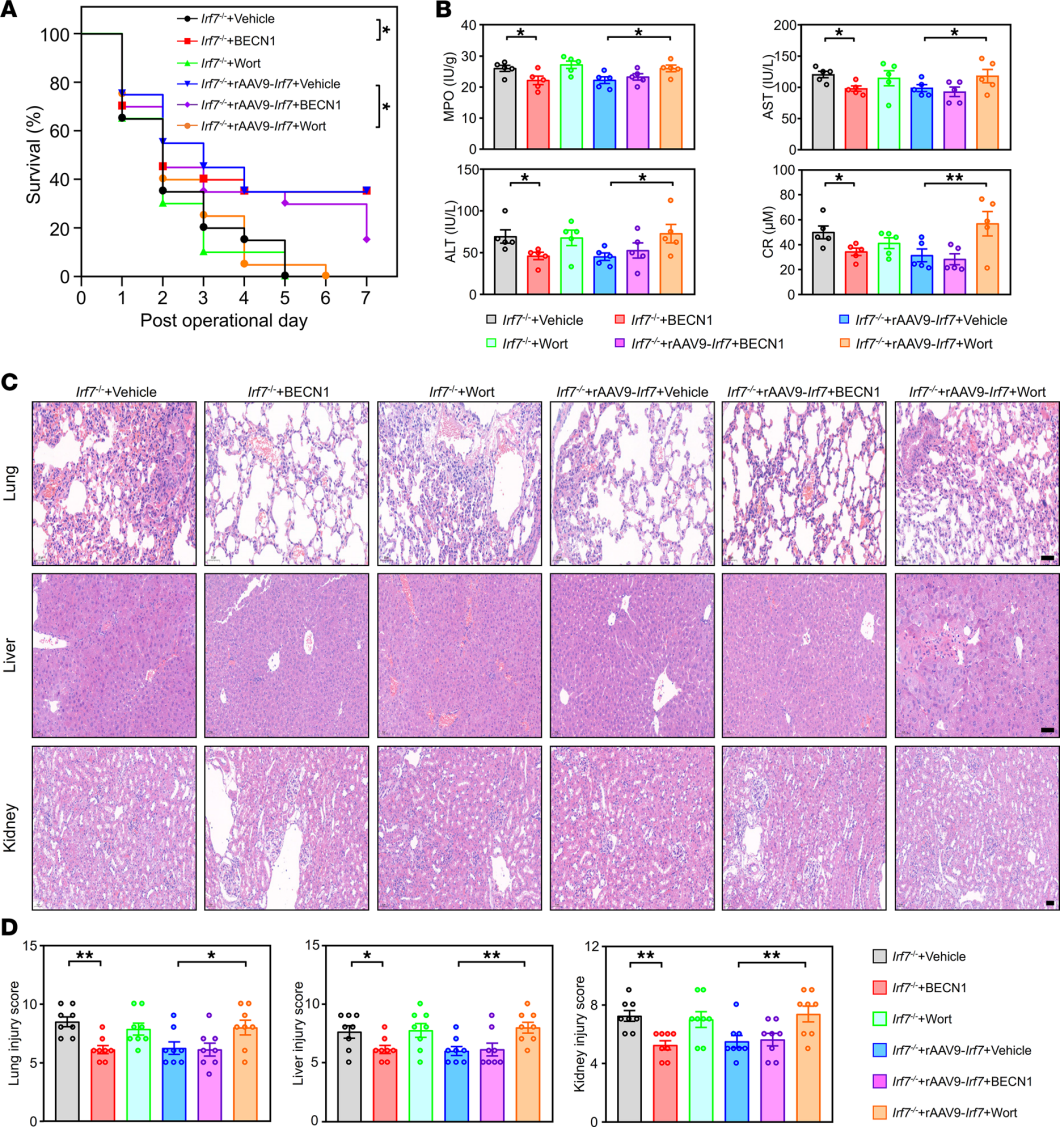
IRF7 defends against polymicrobial sepsis via autophagy. (**A**) Survival rates of mice were determined until 7 days after CLP (*n* = 20, log rank test). *P < 0.05. TAT-Beclin1 (BECN1), 15 mg/kg. Wort, 0.5 mg/kg. rAAV9-*Irf7*, 1 × 10^11^ vg/mouse. (**B**) MPO activity in lung and plasma AST, ALT, CR levels. Data represent the mean ± SEM. *P < 0.05, **P < 0.01, 1-way ANOVA with Bonferroni’s correction. (**C**) H&E staining. Scale bar, 50 μm. (**D**) The corresponding histological scores of lung, liver, and kidney. Data represent the mean ± SEM. *P < 0.05, **P < 0.01, 1-way ANOVA with Bonferroni’s correction.

To determine whether the ability of IRF7 to defend against polymicrobial sepsis is dependent on autophagy, we treated mice that underwent CLP with either BECN1 or Wort. BECN1 significantly increased the survival rate of *Irf7*^–/–^ mice after CLP, whereas Wort could not decrease the rate (Figure 6A). Moreover, in *Irf7*^–/–^ + rAAV9-*Irf7* mice, the survival rate was markedly reduced by Wort (Figure 6A). BECN1 failed to further improve survival in septic *Irf7*^–/–^ mice administrated rAAV9-*Irf7* at a dose of 1 × 10^11^ vg/mouse, which had already achieved comparable maximum survival benefits (Figure 6A and Supplemental Figure 9A). This indicates that rAAV9-*Irf7* mediates sepsis protection in *Irf7*^–/–^ mice via a mechanism that can be activated by BECN1. Furthermore, the levels of MPO, AST, ALT, and CR (Figure 6B) and extent of organ injury (Figure 6, C and D) were dramatically inhibited by BECN1 in *Irf7*^–/–^ mice and increased by Wort in *Irf7*^–/–^ + rAAV9-*Irf7* mice. Taken together, these data suggest that IRF7 defends against polymicrobial sepsis via autophagy.

### IRF7 overexpression alleviates sepsis-induced organ injury.

**Figure 7:**
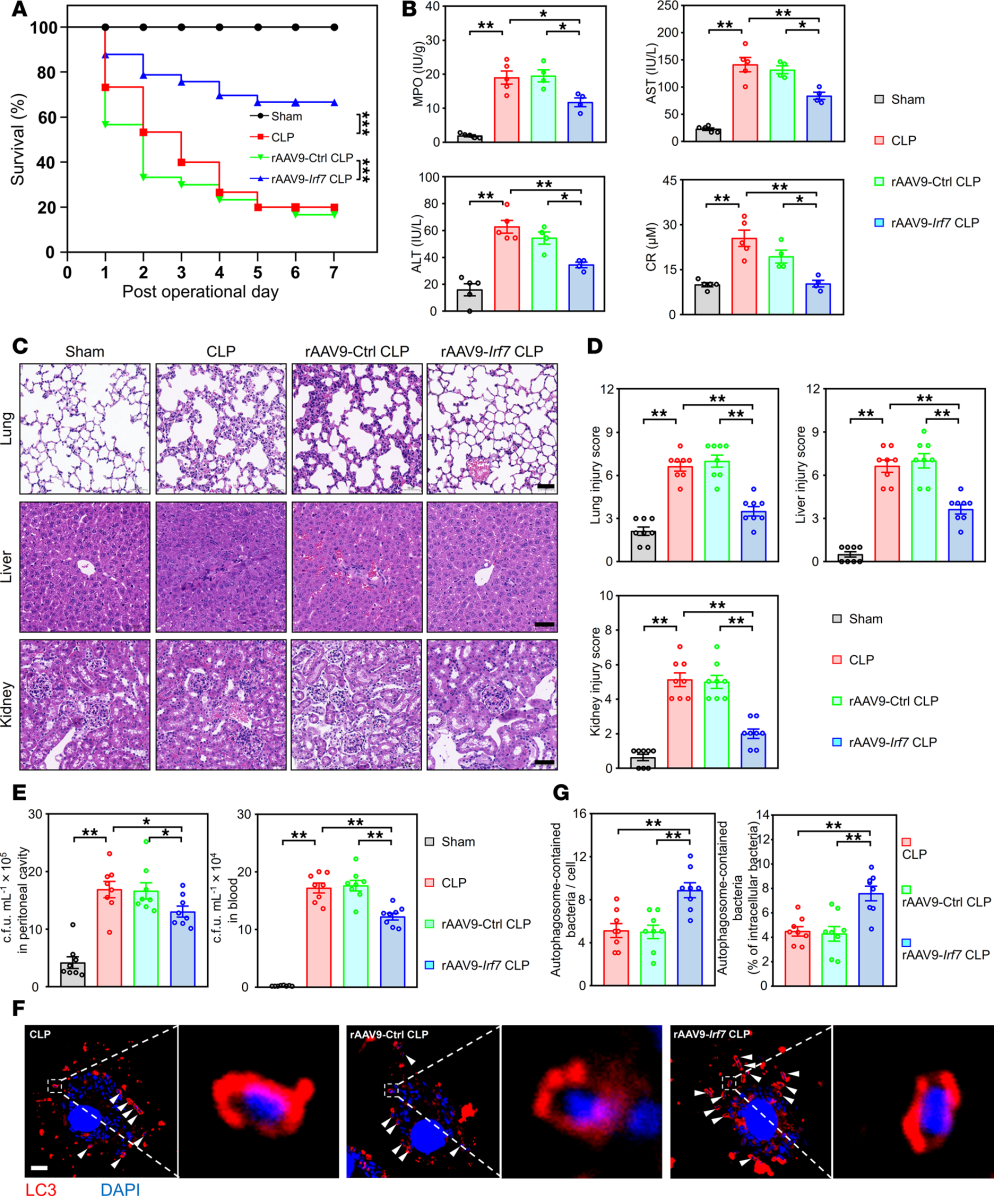
IRF7 overexpression improves sepsis. (**A**) Survival of WT mice subjected to CLP were represented by Kaplan-Meier survival curves (*n* = 10–33, log rank test). ***P < 0.001. rAAV9-*Irf7*, 1 × 10^11^ vg/mouse. (**B**) MPO activity in lung and plasma AST, ALT, CR levels. Data represent the mean ± SEM. *P < 0.05, **P < 0.01, 1-way ANOVA with Bonferroni’s correction. (**C** and **D**) Tissue injury and inflammation of mice that underwent CLP were measured by H&E staining (**C**), and the corresponding histological scores of lung, liver, and kidney were respectively shown in **D**. Scale bar, 50 μm. Data represent the mean ± SEM. **P < 0.01, 1-way ANOVA with Bonferroni’s correction. (**E**) Bacterial loads in the blood and peritoneal cavity were determined by CFU assay. Data represent the mean ± SEM. *P < 0.05, **P < 0.01, 1-way ANOVA with Bonferroni’s correction. (**F** and **G**) Analysis of autophagosomes containing bacteria in peritoneal macrophages of mice that underwent CLP for 16 hours. (**F**) Representative fluorescence pictures. White arrow, LC3-positive vesicles packed with smaller DAPI-positive dots. Scale bar, 5 μm. (**G**) Scatterplot with bar depicts the number of bacteria surrounded with autophagosomes per cell (left panel) and percentage of bacteria surrounded with autophagosomes of the intracellular bacteria (right panel). Data represent the mean ± SEM. **P < 0.01, 1-way ANOVA with Bonferroni’s correction.

Next, to determine whether IRF7 is a target for treating sepsis, we intravenously administrated rAAV9-*Irf7* to WT mice. As shown in Figure 7A, rAAV9-*Irf7* dramatically increased the survival rates of WT septic mice. Additionally, the MPO, AST, ALT, and CR levels (Figure 7B) and degree of organ injury (Figure 7, C and D) were significantly reduced by rAAV9-*Irf7*. Consistent with these findings, rAAV9-*Irf7* inhibited apoptosis in the lungs, livers, and kidneys after CLP (Supplemental Figure 10, A and B). Furthermore, rAAV9-*Irf7* markedly decreased the bacterial loads in both the peritoneal cavity and blood of WT septic mice (Figure 7E). In peritoneal macrophages from WT septic mice, rAAV9-*Irf7* dramatically increased the number of bacteria in autophagosomes (Figure 7, F and G). Correspondingly, this protective effect was significantly abrogated by Wort treatment (Supplemental Figure 10C). In sum, inducing IRF7 expression via rAAV9-*Irf7* might promote the autophagic clearance of pathogens to improve sepsis outcomes.

## Discussion

Autophagy is believed to be an emerging therapeutic strategy for sepsis (24–26), but its underlying regulatory factors remain unknown. In this study, we revealed that IRF7 induced autophagy to drive macrophages to execute an antimicrobial defense during polymicrobial sepsis.

### IRF7 promotes the autophagic clearance of pathogens in macrophages to defend against polymicrobial sepsis.

The results suggested that IRF7 was stimulated to defend against sepsis. Many studies have proven IRF7 is a master regulator of type I IFN responses. Through the MyD88-IRAK4-IRAK1-TRAF6 pathway, IRF7 is activated to trigger large amounts of type I IFN in DCs (27). In monocytes/macrophages, IRF7 forms a heterodimer with IRF3 to induce type I IFN (28, 29). Unexpectedly, the expression of type I IFN genes (*Ifna* and *Ifnb*) peaked at the early stage of sepsis, but *Irf7* expression was high at the late stage (Supplemental Figure 11A). Moreover, *Irf7* deficiency did not inhibit *Ifna* or *Ifnb* expression (Supplemental Figure 11, B and C), suggesting that type I IFN responses are not the IRF7-driven determinants of peritoneal macrophage-mediated sepsis improvement. Therefore, identifying the underlying mechanism that IRF7 defends against sepsis is important to find new measures for sepsis therapy and new functions of IRF7.

Because DCs constitutively express high levels of IRF7 (30), the kinetics of IRF7 expression in peritoneal cells after CLP also suggest that DCs might not be the primary cells required for IRF7 to defend against sepsis. The results in the WT IN and *Irf7*^–/–^ IN mice revealed that IRF7 drove macrophages to defend against sepsis. Thus, it may be speculated that IRF7 modulates the functions of mature macrophages. This view is consistent with the known role of IRF7 as a regulator that participates in macrophage differentiation (31) and polarization (32). As scavengers, the basic function and one of the key characteristics of mature macrophages is their clearance of invading pathogens. This research revealed that IRF7 drove macrophages to kill *E*. *coli*, *S*. *typhimurium*, and *V*. *vulnificus*. Furthermore, IRF7 might limit *E*. *trada* infection (33). In the splenic marginal zone, IRF7 is required for the macrophage-mediated killing of *L*. *donovani* (34). The diversity of bacterial species killed due to IRF7 regulation not only indicates that IRF7 nonselectively eliminates pathogens but also suggests that IRF7 could regulate a conservative cellular response during evolution to clear microorganisms.

Autophagy, an evolutionarily conserved cellular process, is believed to be one innate immune defense mechanism against microbial challenge (35) and plays a protective role in septic animals (6). Identifying a key regulator triggering the autophagic response upon septic insult would thus be ideal. Increasing amounts of data have demonstrated that transcriptional control is the master mechanism in autophagy. For instance, transcription factor EB and FOXO3 are activated to trigger autophagy after starvation (10, 36). Research has also shown that IRF7 directs ATGs to trigger autophagy. In vitro, IRF1 inhibited macrophage autophagy and induced apoptosis via inducible nitric oxide synthase following LPS administration (15). Keiko’s group reported that IRF8 is required for LPS/IFN-γ–induced autophagy to clear *L*. *monocytogenes* by binding to and stimulating ATGs (11). Taken together, these findings also suggest that the IRF family comprises important transcription factors involved in controlling autophagy in response to infection.

Autophagic flux is initiated with the elongation of phagophores that engulf cytoplasmic materials, including intracellular microorganisms. Then, an autophagosome forms when the edges of the phagophore fuse. Autophagosomes then fuse with lysosomes to become autolysosomes, which degrade invading pathogens. IRF7 directs a few ATGs involved in autophagosome and autolysosome formation. Structural imaging data (Supplemental Figure 12) further revealed that IRF7 facilitates smooth autophagic flux, indicating that IRF7 is necessary for engulfing and degrading pathogens via autophagy.

IRF3 and IRF7 both regulate the transcription of type I IFN genes (13), but their effects in infected hosts have been debated. In acute pyelonephritis, IRF3 is required for mucosal innate immunity and protects the kidney from uropathogenic *E*. *coli*, whereas IRF7 agonism triggers a hyperinflammatory response and increases kidney injury (37). Moreover, in an animal model, IRF3 drove the pathogenesis of sepsis (38, 39). Unlike the effects of IRF3 on sepsis, our study revealed that IRF7 was activated to clear bacteria and ultimately reduced the death rate of septic mice. These studies suggest that these 2 master regulators of type I IFNs have different effects during different infectious diseases. However, what is the reason for this phenomenon? First, IRF3 is constitutively expressed and serves as a prime initiator of type I IFNs, but IRF7 is activated in the late phase of infectious disease and acts as a master regulator to trigger the transcription of type I IFN genes and IFN-stimulated genes (14). Therefore, the inflammatory response in different phases might play different roles in one disease. Beyond canonical type I IFN regulation, IRFs exhibit noncanonical functions, exemplified by our discovery of IRF7-mediated autophagy modulation. Critically, both our study and Qing et al. (40) redefine IRF7 as a master regulator of IFN-independent autophagy in macrophages. However, while we observe *E*. *coli* clearance through IRF7’s direct binding to ATG gene promoters (enhancing autophagic flux), Qing et al. report *Candida albicans* subversion of IRF7 for autophagy suppression via mTOR activation. The functional dichotomy may stem from pathogen-specific evasion strategies. While macrophages typically deploy autophagy to eliminate bacteria (41), certain pathogens like *C*. *albicans* and *Mycobacterium tuberculosis* (42) actively subvert this pathway for immune escape. Collectively, IRF7 may operate as a pathogen-adaptable switch, dynamically redirecting autophagy toward host defense or pathogen advantage, necessitating tailored therapeutic interventions targeting stimulus-specific mechanisms.

### IRF7 overexpression might be a nonantibiotic therapy for sepsis.

Because sepsis initiates from the proliferation of microbes, antibiotics are the key therapeutic drugs used for killing bacteria in patients with sepsis. However, considering that antibiotic resistance, organ injury, mitochondrial dysfunction, and microbiome disruption are caused by the inappropriate use of antibiotics, new treatments based on the intracellular mechanism of self-defense are being investigated for fighting invading bacteria in septic hosts. Autophagy is a cell-autonomous defense mechanism that involves the dissolution of cellular debris and intracellular pathogens. The findings of this study suggest that IRF7 might be a therapeutic target for improving cell-autonomous defenses to replace the antibiotics used in sepsis treatment.

An intriguing question, then, is how to trigger IRF7 to protect septic animals. We found that IRF7 might be stimulated by the IFN-β/JAK1/STAT3 pathway in polymicrobial sepsis (Supplemental Figure 13). In this study, we treated CLP mice with IFN-β. Unfortunately, IFN-β did not increase the survival of septic mice (Supplemental Figure 14A). These results might indicate that IFN-β is not the only trigger of IRF7 during sepsis such that IFN-β cannot fully stimulate IRF7 to improve sepsis outcomes. Moreover, these data might suggest that IFN-β not only triggers IRF7 to defend against sepsis but also plays a detrimental role in septic mice, which has been supported by 2 opposing studies. Kelly-Scumpia et al. reported that type I IFN signaling was required for the survival of CLP mice (43), but Dejager’s team reported that inhibiting type I IFN signaling protected septic mice (44). Anyway, IFN-β is not an ideal agonist for stimulating IRF7 to protect mice from polymicrobial sepsis.

Viral vectors are frequently used tools for gene delivery. To date, the 5 most commonly used strategies involve adenoviruses, lentiviruses, the herpes simplex virus, the vaccinia virus, and AAVs (45, 46). Owing to their natural propensity to persist in human cells and lack of known pathology, AAV vectors have the greatest potential for gene therapy in the clinic (47). In this study, WT mice were injected with rAAV9-*Irf7*, and the results showed that IRF7 overexpression promoted the autophagic clearance of pathogens and improved sepsis outcomes, establishing its potential as a nonantibiotic therapy. However, clinical translation requires overcoming preexisting immunity against AAV serotypes and the need for macrophage-specific targeting to prevent off-organ toxicity. To address these, future efforts should integrate biomarker-guided patient stratification using autophagic flux deficiency or antibiotic resistance profiles, develop combinatorial regimens pairing pathogen-targeted antibiotics with immunomodulators, and engineer tissue-specific AAV capsids for precision delivery. While clinical AAV experience in sepsis remains limited, recent successes substantiate the feasibility of this approach. AAV-mediated SOD delivery reduces sepsis-associated lung injury (48), while Arc overexpression rescues cognitive impairment (49). Furthermore, transformative clinical successes in other fields, such as alongside FDA-approved AAV9-based Zolgensma and AAV5-based Hemgenix achieving durable remission in spinal muscular atrophy and hemophilia B, respectively (50, 51), collectively validate targeted gene augmentation. These advances, coupled with emerging immunotoxicity mitigation strategies, provide a robust roadmap for translating IRF7-based therapies into clinical practice for sepsis.

## Methods

### Sex as a biological variable.

Our study examined male mice because male animals exhibited less variability in phenotype. It is unknown whether the findings are relevant for female mice.

### Animals.

Male 8- to 10-week-old C57BL/6 mice were used. *Irf7*^–/–^ mice were purchased from Cyagen Biosciences. These mice were housed under specific pathogen–free conditions on a 12-hour light/12-hour dark cycle with free access to food and water.

The CLP model was established as described previously (19). In brief, the mice were anesthetized with ketamine and xylazine. Then a midline laparotomy (2 cm) was performed under aseptic conditions to exteriorize the cecum. Fifty percent of the cecum was ligated with a silk suture and completely pierced once with a 21 G needle, followed by the extrusion of a small drop of fecal material. Finally, the exposed cecum was returned to the abdominal cavity and the abdomen was closed. For the sham mice, the cecum was exposed and replaced within the peritoneal cavity without ligation or puncture. The mice were resuscitated by applying 1 mL of warm saline subcutaneously after surgery.

rAAV9-*Irf7* (GeneChem) was intravenously injected into WT or *Irf7*^–/–^ mice 2 weeks before surgery. BECN1 or Wort was intraperitoneally injected 1 hour post-CLP.

### Cells.

Bone marrow cells were isolated from C57BL/6 mice and cultured in complete medium (DMEM, 10% FBS, 100 U/mL penicillin, and 100 μg/mL streptomycin) in the presence of 20 ng/mL mouse M-CSF (Miltenyi Biotec, 130-101-704) for 7 days to generate macrophages (52). The cells were then treated with LPS (100 ng/mL) for 16 hours. For bacterial infection, BMDMs were infected with bacteria at a bacteria-to-cell ratio of 50:1 (MOI of 50). Peritoneal macrophages were purified with anti-F4/80 microbeads (Miltenyi Biotec, 130-110-443), according to the manufacturer’s instructions.

### Measurement of MPO levels and examination of the serum.

Lung and blood samples were collected at 16 hours after CLP. The lungs were excised and snap-frozen in liquid nitrogen until the MPO level was measured. Blood samples were harvested in coagulative microcentrifuge tubes, placed at room temperature for 30 minutes, and centrifuged for 10 minutes at 8,000*g*. Finally, the serum was collected. The levels of MPO in the lung and AST, ALT, and CR in the serum were detected with commercial assay kits (Nanjing Jiancheng, A044-1-1 for MPO, C010-3-1 for AST, C009-3-1 for ALT, and C011-2-1 for CR) according to the manufacturer’s instructions to evaluate lung, liver, and kidney function.

### H&E and TUNEL staining.

Formalin-fixed tissues were embedded in paraffin and cut into 5 μm–thick sections. The sections were stained with H&E, then analyzed for inflammation and tissue damage via microscopy (Life Technology, EVOS XL Core). The sections were subjected to TUNEL staining using a one-step TUNEL kit (Keygen, KGA1405), then analyzed for apoptotic cells via fluorescence microscopy (Carl Zeiss, Axio Observer A1).

### ELISA.

Blood from mice was acquired and centrifuged at 8,000*g* for 5 minutes at 4°C. The serum was used for quantification of TNF-α (NeoBioscience, EMC102a), IL-1β (Elabscience, E-EL-M0037), IL-6 (Elabscience, E-EL-M0044), IL-12 (Elabscience, E-EL-M3062), and IFN-γ (Elabscience, E-EL-M0048) with ELISA kits following the manufacturers’ protocols.

### Depletion of cells in vivo.

For depletion of macrophages (53), mice were given intraperitoneal injection of 150 μL CL (Liposoma, C-005) 24 hours prior to CLP modeling. For depletion of T cells (54), mice were daily intravenously injected with 100 μg InVivoMAb anti-CD3 antibody (BioXCell, BE0001-1) 5 days prior to surgery. For depletion of NK cells (55), mice were intraperitoneally injected every second day with 50 μg of InVivoMAb anti-mouse NK1.1 antibody (BioXCell, BE0036) 6 days prior to surgery.

### Adoptive transfer of peritoneal macrophages.

Adoptive transfer was performed as described previously (7). In brief, thioglycolate-elicited peritoneal cells (56) from WT or *Irf7*^–/–^ mice were collected in 10 mL PBS. And 3 × 10^6^ purified peritoneal macrophages were intraperitoneally injected into recipient WT CL mice 1 hour after CLP.

### RNA interference.

According to the manufacturer’s instructions, BMDMs were transfected with the *Irf7* gene or *Irf7* siRNA (Genepharma) with Lipofectamine 3000 (Invitrogen, L3000015) or Lipofectamine RNAiMAX transfection reagent (Invitrogen, 13778150), respectively. In brief, the *Irf7* gene, Lipofectamine 3000, *Irf7* siRNA, and Lipofectamine RNAiMAX were diluted in Opti-MEM reduced serum medium (Invitrogen). Then, the *Irf7* gene was mixed with Lipofectamine 3000, and *Irf7* siRNA was mixed with Lipofectamine RNAiMAX before incubation for 5 minutes at room temperature. The cells were incubated with the transfection mixture for 24 hours and then analyzed. The *Irf7* gene was constructed in the pcDNA3.1-HA vector. The *Irf7* siRNA consisted of 3 pooled 19-nucleotide duplexes (CUUGCGCCAAGACAAUUCA, CUGGAUGUGACCAUCAUGU, GCACUUUCUUCCGAGAACU).

### RT-qPCR.

Total RNA was isolated using TRIzol reagent (Invitrogen, 15596018CN), then reverse-transcribed using the ReverTra Ace qPCR RT Kit (TOYOBO, FSQ-101). Quantitative PCR was performed on an ABI 7500 system (Applied Biosystems) using the SYBR green fluorescence system. The primers used in this study are listed in Supplemental Table 1. The *Actin* gene was used for normalization.

### Western blot.

Total cell lysates were extracted using RIPA solution supplemented with phenylmethylsulfonyl fluoride and protease inhibitor cocktail. Proteins were separated via SDS-PAGE and transferred to PVDF membranes. The membranes were incubated with primary antibodies against IRF1 (Cell Signaling Technology, 8478, 1:1,000), IRF2 (Cell Signaling Technology, 76647, 1:1,000), IRF3 (Cell Signaling Technology, 11904, 1:1,000), IRF4 (Cell Signaling Technology, 62834, 1:1,000), IRF5 (Cell Signaling Technology, 96527, 1:1,000), IRF6 (MedChemExpress, HY-P82917, 1:1,000), IRF7 (Cell Signaling Technology, 72073, 1:1,000), IRF8 (Cell Signaling Technology, 98344, 1:1,000), IRF9 (Cell Signaling Technology, 28845, 1:1,000), LC3 (Novus Biologicals, NB100-2220, 1:1,000), p62 (Cell Signaling Technology, 5114, 1:1,000), JAK1 (Cell Signaling Technology, 3344, 1:1,000), p-JAK1 (Cell Signaling Technology, 3331, 1:1,000), STAT3 (Cell Signaling Technology, 4904, 1:1,000), p-STAT3 (Cell Signaling Technology, 9131, 1:1,000), ULK1 (Cell Signaling Technology, 8054, 1:1,000), Beclin1 (Cell Signaling Technology, 3,495, 1:1,000), ATG7 (Cell Signaling Technology, 8,558, 1:1,000), RAB7 (Cell Signaling Technology, 9,367, 1:1,000), LAMP2 (Cell Signaling Technology, 49067, 1:1,000), DRP1 (Cell Signaling Technology, 8570, 1:1,000), or Actin (Cell Signaling Technology, 4967, 1:1,000) overnight at 4°C. Immunoreactivity was visualized by ECL kit (MilliporeSigma, WBULS0100).

### Immunofluorescence staining.

Peritoneal macrophages and BMDMs were fixed with 4% paraformaldehyde, permeabilized with 0.5% Triton X-100 for 10 minutes, and incubated with primary antibodies against LC3 (Novus Biologicals, NB100-2220, 1:100) and IRF7 (Cell Signaling Technology, 72073, 1:100) overnight at 4°C. The macrophages were then stained with a goat anti-rabbit IgG secondary antibody conjugated with Alexa Fluor 488 (Molecular Probes, A-11008, 1:100) or Alexa Fluor 546 (Molecular Probes, A-21085, 1:100) for 2 hours at room temperature, then counterstained with DAPI for 10 minutes. The stained cells were analyzed using confocal microscopy (Carl Zeiss, LSM 780).

### Transmission electron microscopy.

Macrophages were fixed with 2.5% glutaraldehyde, treated with 1% osmium tetroxide, and then dehydrated with a series of ethanol. After infiltration, the cells were embedded in Polybed 812 epoxy resin. Ultrathin sections were cut using an ultramicrotome and a diamond knife, collected on 200-mesh copper grids, and counterstained with 5% uranyl acetate and lead citrate. The samples were then examined at 60 kV using a Hitachi H7500 instrument.

### ChIP-Seq.

BMDMs were cross-linked and lysed, and chromatin was fragmented by micrococcal nuclease. The lysates were immunoprecipitated with an anti-IRF7 antibody (MilliporeSigma, ABF130). The immunocomplexes were then purified to collect immunoprecipitated DNA, which was used to generate a sequencing library and sequenced using the HiSeq 2500 system (Illumina). The IRF7-binding genes are listed in Supplemental Table 2 and were subjected to KEGG analysis. Integrative Genomics Viewer was used to show IRF7-binding ATGs.

### Bacterial killing assay.

Intracellular bacterial killing was determined as described previously (7, 57). Briefly, BMDMs were cultured in 24-well plates (2 × 10^5^ cells/well) and infected with bacteria at an MOI of 50 for 45 minutes. The macrophages in one of duplicate wells were washed with 0.01% EDTA and lysed with 0.1% Triton X-100. After serial 10-fold dilutions, the number of internalized bacteria was determined via a CFU assay [CFU (45 minutes)]. A second series of internalization assays was run in parallel. For this assay, the cells were infected for 45 minutes, after which 100 μg/mL gentamicin (Sigma) was added for 1 hour to kill the extracellular bacteria. After 1 hour of further cultivation, the cells were lysed to analyze the viable intracellular bacteria [CFU (45 minutes + 2 hours)]. The intracellular bacterial killing rate was calculated as follows: {[CFU (45 minutes) – CFU (45 minutes + 2 hours)] / CFU (45 minutes)} × 100%.

### Assessment of bacterial load.

Fifty percent of the cecum was ligated as described for the animal model and excised. The excised ceca were scraped with the back of a sterile scalpel to collect feces, which were placed in 10 mL of precooled PBS. The fecal materials were placed on ice until bacteriological analysis was performed.

Peritoneal lavage fluid was collected as previously described (58). Briefly, 5 mL of PBS was slowly injected into the peritoneal cavity using an 18 G needle after the abdominal wall was exposed. The abdomen was gently massaged for 1 minute, after which the peritoneal fluid was collected with the needle. To harvest as much of the bacteria in the peritoneal cavity as possible, another 5 mL of PBS was injected and recovered again. The peritoneal fluid was placed on ice for further experiments. Next, the abdominal wall was opened, and the intestines were exteriorized to expose the postcava. Blood samples were obtained from the postcaval vein, placed into heparinized microcentrifuge tubes, and kept on ice (59).

After serial dilutions, the bacterial loads in the fecal materials, lavage fluid, and blood were determined via a CFU assay.

### Statistics.

Statistical analyses were performed with SPSS 24 (IBM) using log rank test (survival curves) or 1-way ANOVA. The data are presented as the means ± SEM. *P* < 0.05 was considered statistically significant.

### Study approval.

All animal experiments were approved by the Animal Welfare and Ethics Committee of Southern Medical University and performed in accordance with institutional guidelines.

### Data availability.

All data are included in the Supporting Data Values file. Any data that support the findings of this study are available from the corresponding author upon reasonable request. The ChIP-Seq data used in this study were deposited in the Genome Sequence Archive of the National Genomics Data Center with accession number CRA030059.

## Author contributions

The project was conceptualized by YJ. Methodology was provided by GC. Formal analyses were performed by YJ, LZ, and HL. Investigation was performed by GC and KL. The original draft of the manuscript was written by GC and KL. Reviewing and editing of the manuscript were performed by YJ and LZ. Visualization was provided by GC, KL, HL, and YJ. Supervision was performed by YJ and LZ. Project administration was provided by YJ. Funding was acquired by YJ and LZ.

GC (first position) and KL (second position) are co–first authors. GC initiated the study and performed initial experiments. KL performed the majority of revision experiments and data analysis. Authorship order reflects their relative contributions to the work.

## Funding support

Grants from the National Natural Science Foundation of China (82241061) to YJ.

Guang Dong Basic and Applied Basic Research Foundation (2022B1515120024) to YJ.

Special Support Plan for Outstanding Talents of Guangdong Province (2019JC05Y340) to YJ.

Grants from the Training Program of the Clinical Research Plan of Southern Medical University (LC2016PY031) to LZ.

## Supplementary Material

Supplemental data

Unedited blot and gel images

Supporting data values

## Version 1 11/10/2025

Electronic publication
