# Supplementary material for: IRF7 drives macrophages to kill bacteria and improves septic outcomes via autophagy
Source: JCI Insight. 2025 Nov 10;10(21):e189420. doi: 10.1172/jci.insight.189420 (PMC12643508; doi:10.1172/jci.insight.189420)
Supplement: Supplemental data [file jciinsight-10-189420-s008.pdf]

IRF7 drives macrophages to kill bacteria and improves septic outcomes via autophagy

Guiming Chen[1,2,3,4#], Kangxin Li[4,5,6#], Haihua Luo[2], Lianxu Zhao[1*], Yong Jiang[2,3,4,5,6*]

[1]Department of Neurology, Shenzhen Hospital, Southern Medical University, Shenzhen, Guangdong, China.

[2]Guangdong Provincial Key Laboratory of Proteomics, State Key Laboratory of Organ Failure Research, School of Basic Medical Sciences, Southern Medical University, Guangzhou, Guangdong, China.

[3]Department of Respiratory and Critical Care Medicine, The Tenth Affiliated Hospital (Dongguan People's Hospital), Southern Medical University, Dongguan, Guangdong, China.

[4]State Key Laboratory of Metabolic Dysregulation & Prevention and Treatment of Esophageal Cancer, The First Affiliated Hospital, Zhengzhou University, Zhengzhou, Henan, China.

[5]Henan Key Laboratory of Critical Care Medicine, Department of Emergency Medicine, The First Affiliated Hospital, Zhengzhou University, Zhengzhou, Henan, China.

[6]Institute of Infection and Immunity, Henan Academy of Innovations in Medical Science, Zhengzhou, Henan, China.

[#]Co-first authors

[*]Co-corresponding authors

Contact for correspondence:

Yong Jiang
State Key Laboratory of Metabolic Dysregulation & Prevention and Treatment of Esophageal Cancer
The First Affiliated Hospital of Zhengzhou University
No.1 Jianshe Dong Road, Erqi District, Zhenzhou, Henan 450001, China
Tel: 86.0371.66278801
jiang48231@163.com

Lianxu Zhao
Department of Neurology
Shenzhen Hospital of Southern Medical University
No.1333 Xinhu Road, Baoan District, Shenzhen, Guangdong 518000, China
Tel: 86.0755. 23360590
zhaolianxu@163.com

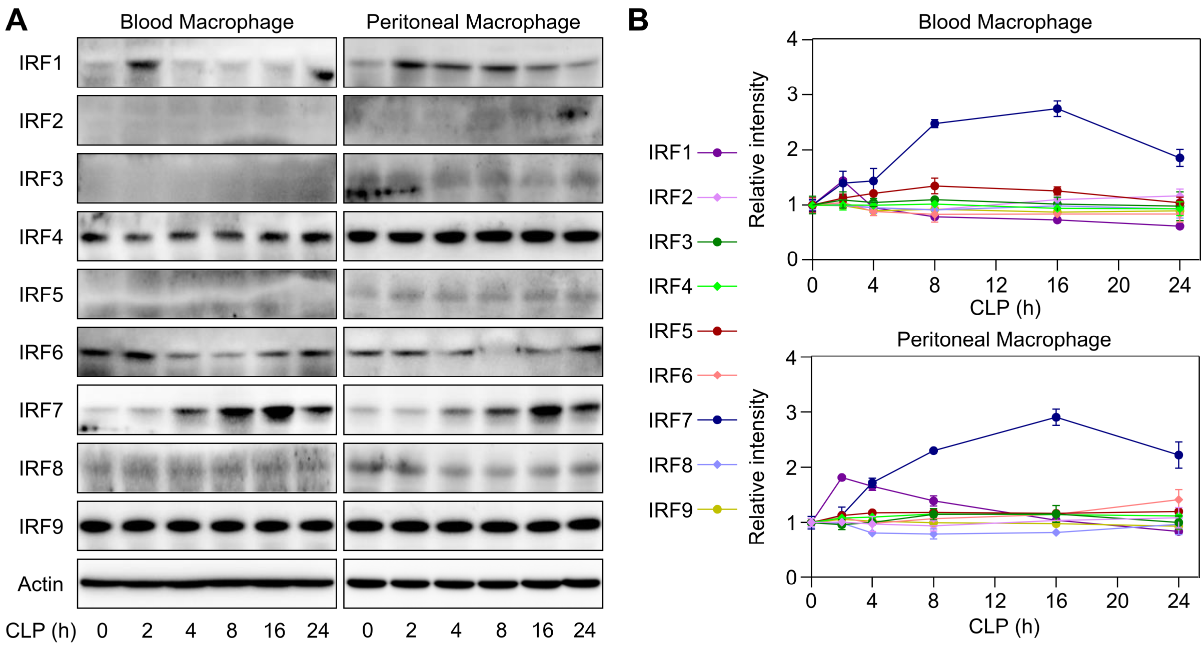

Supplemental Figure 1. IRF7 was stimulated on the late stage of polymicrobial sepsis. (**A**) Representative immunoblots detecting IRF1, IRF2, IRF3, IRF4, IRF5, IRF6, IRF7, IRF8 and IRF9 in blood or peritoneal macrophages isolated from septic mice. (**B**) Line chart of the corresponding intensity of **A**.

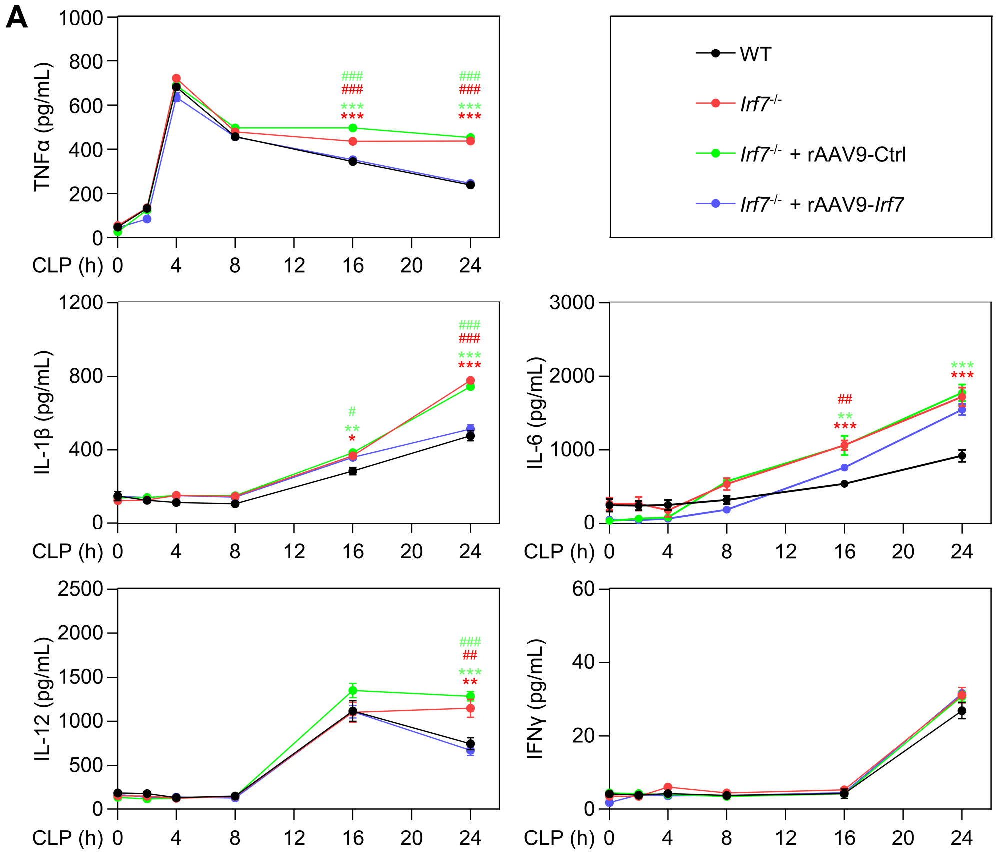

Supplemental Figure 2. *Irf7* deficiency enhanced the inflammatory response on the late phase of polymicrobial sepsis. (**A**) Pro-inflammatory cytokines, including TNFα, IL-1β, IL-6, IL-12 and IFNγ in the serum from septic mice were determined by ELISA assay. Data represent the mean ± SEM. * $P < 0.05$ (*vs.* WT), ** $P < 0.01$ (*vs.* WT), *** $P < 0.001$ (*vs.* WT); # $P < 0.05$ (*vs.* *Irf7*$^{-/-}$+rAAV9-*Irf7*), ## $P < 0.01$ (*vs.* *Irf7*$^{-/-}$+rAAV9-*Irf7*), ### $P < 0.001$ (*vs.* *Irf7*$^{-/-}$+rAAV9-*Irf7*). One-way ANOVA.

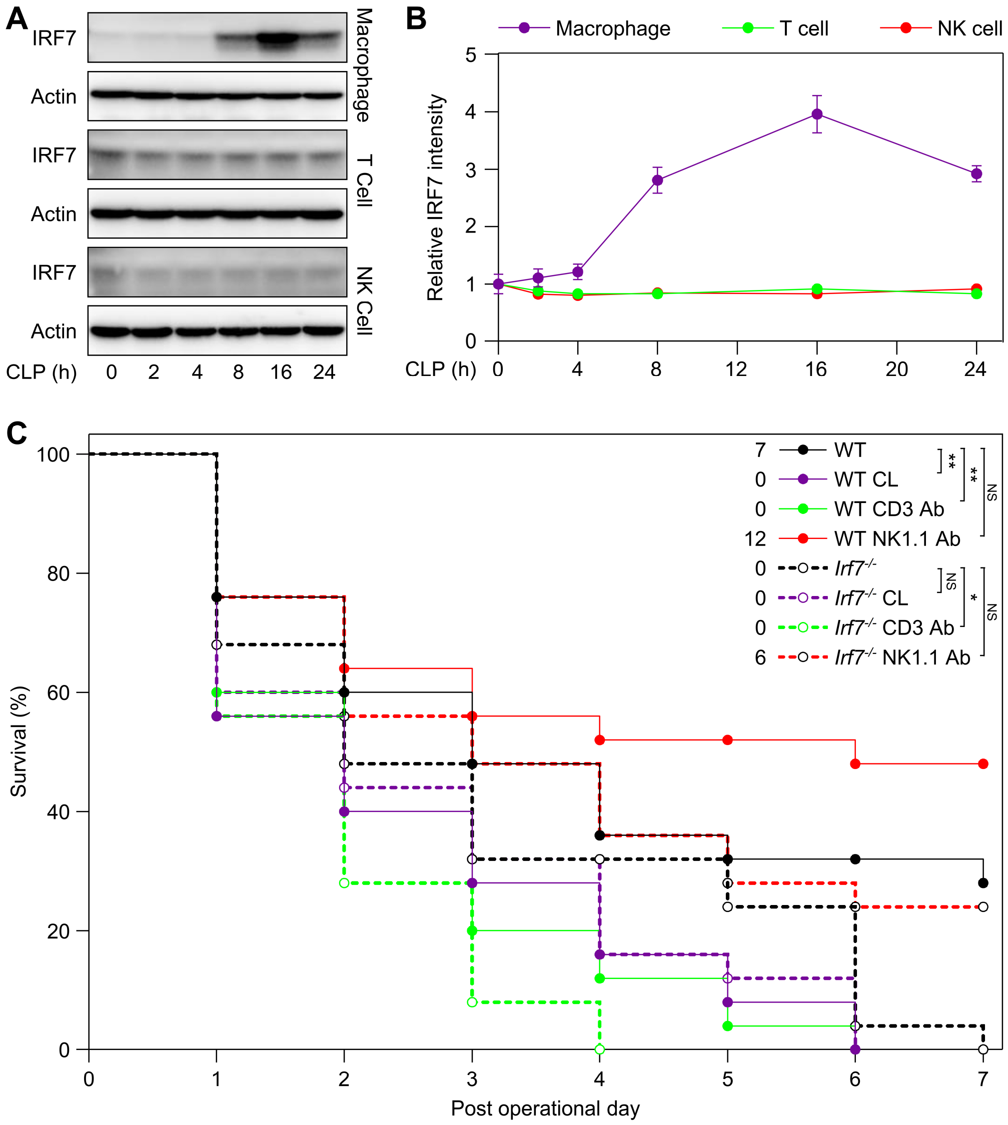

Supplemental Figure 3. IRF7 drove macrophages, but not T cells or NK cells to modulate sepsis development. (**A**) Representative immunoblot detecting IRF7 in macrophages, T cells and NK cells isolated from septic mice. (**B**) Line chart of the corresponding protein intensity of **A**. (**C**) Macrophages, T cells or NK cells in mice were depleted by using clodronate liposomes (CL), anti-CD3 antibodies (CD3 Ab) or anti-NK1.1 antibodies (NK1.1 Ab), respectively. Survival rates of mice were determined until 7 days after CLP (n = 25, log rank test). The left-side numbers in group labels represent surviving mouse counts at the 7-day endpoint post-surgery. NS (not significant) $P \geq 0.05$, * $P < 0.05$, ** $P < 0.01$.

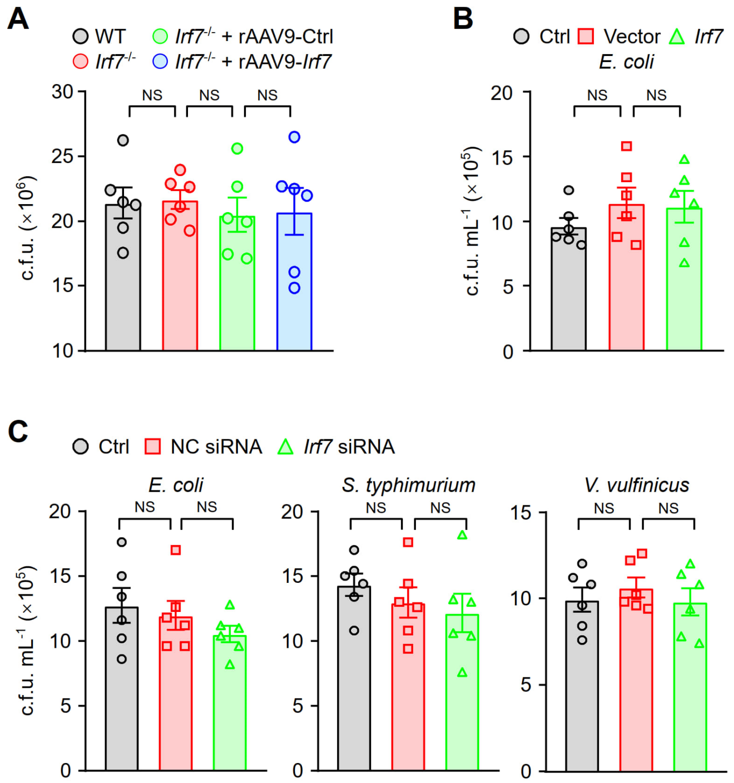

Supplemental Figure 4. IRF7 had no effect on taking up bacteria by macrophages. (**A**) Total fecal bacteria from ligated cecum. Data represent the mean ± SEM. NS $P \geq 0.05$, one-way ANOVA. (**B**, **C**) Total intracellular bacteria in macrophages infected with bacteria for 45 min. Data represent the mean ± SEM. NS $P \geq 0.05$, one-way ANOVA.

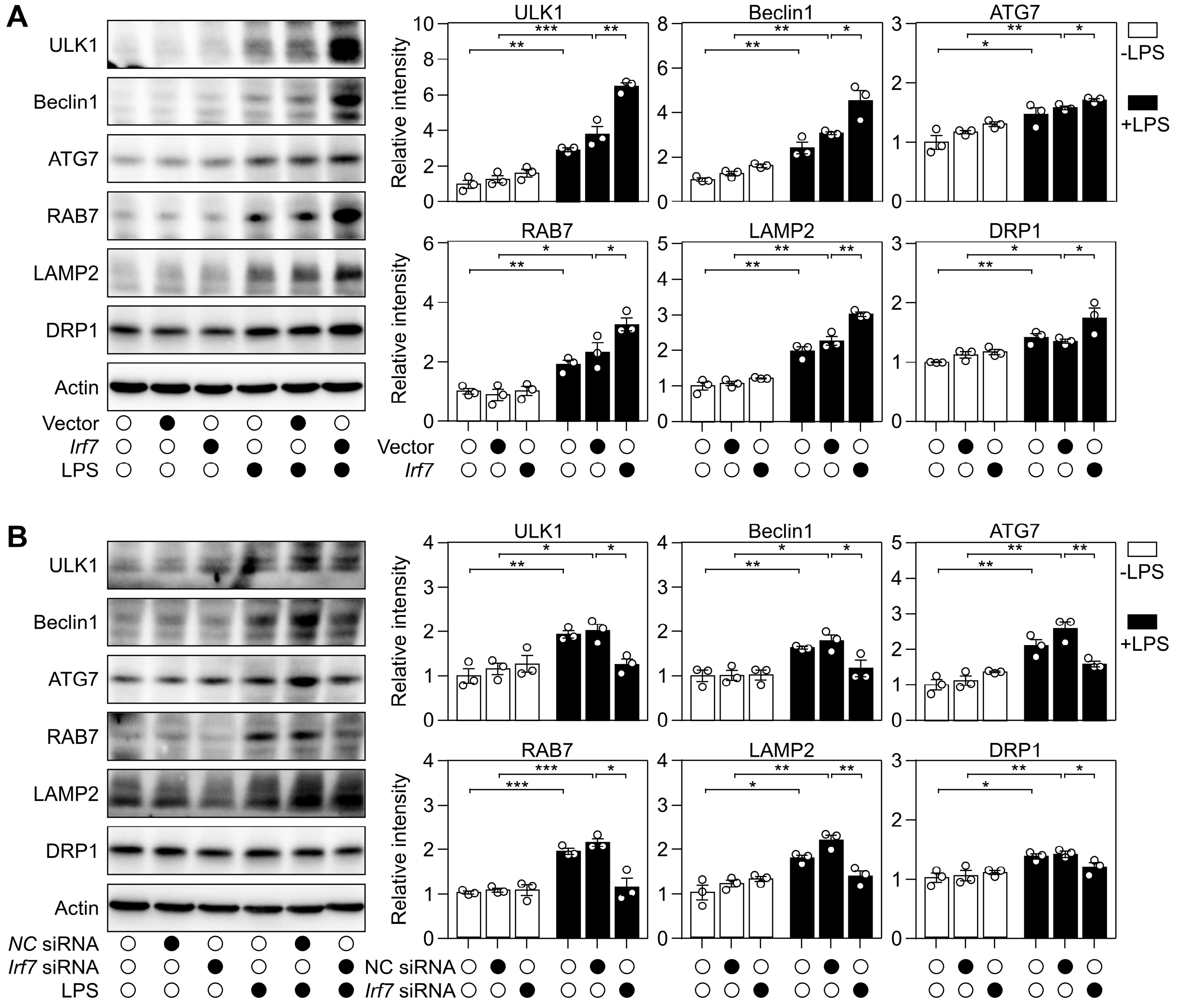

Supplemental Figure 5. IRF7 promoted the ATGs expression. (**A, B**) Representative immunoblots detecting ULK1, Beclin1, ATG7, RAB7, LAMP2 and DRP1 in cellular lysates isolated from BMDMs transfected with (**A**) either empty vector or *Irf7* gene, (**B**) either *NC* siRNA or *Irf7* siRNA. Data represent the mean ± SEM. * $P < 0.05$, ** $P < 0.01$, *** $P < 0.001$, one-way ANOVA.

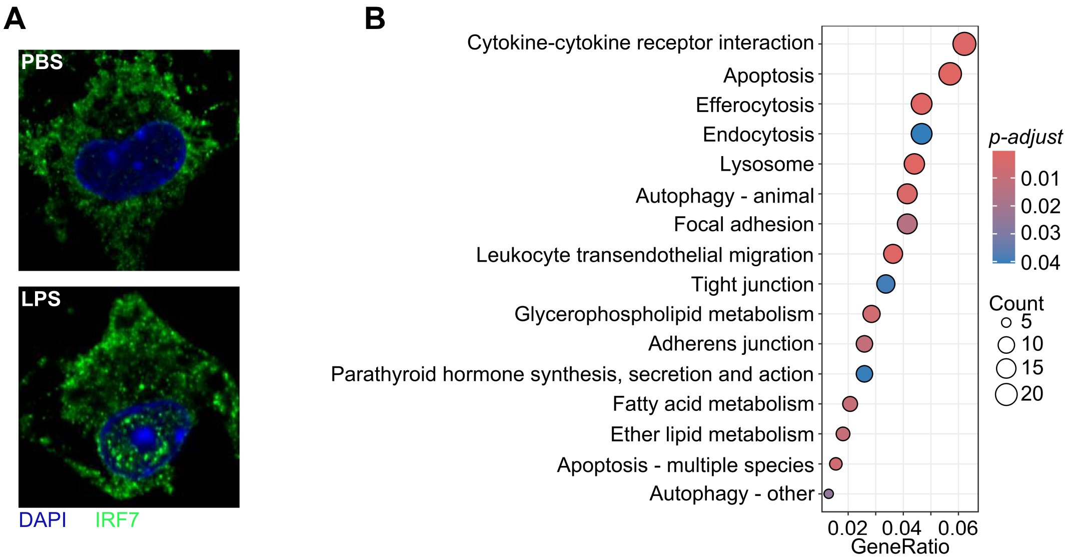

Supplemental Figure 6. IRF7 translocated into nucleus to direct transcription after LPS treatment. (**A**) IRF7 translocated into nucleus. BMDMs were treat with either PBS or 100 ng/mL LPS for 16 hours. Cells were stained with anti-IRF7 antibody and DAPI. (**B**) IRF7-binding Genes were subjected to Kyoto Encyclopedia of Genes and Genomes (KEGG) pathway enrichment analysis.

A

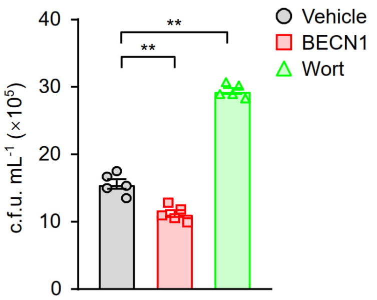

Supplemental Figure 7. Autophagy was involved in eliminating pathogens during sepsis. (**A**) Bacterial loads in peritoneal cavity of WT septic mice. WT mice were underwent CLP and administrated with either Tat-Beclin1 (BECN1) or Wortmannin (Wort). After 16 hours, bacteria were collected from peritoneal cavity, and the counts were determined by colony forming unit assay. Data represent the mean ± SEM. ** P < 0.01, one-way ANOVA.

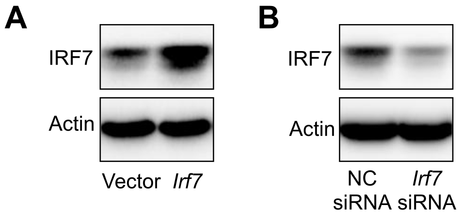

Supplemental Figure 8. Manipulating IRF7 expression in BMDMs. (**A**) Western blot results showed that IRF7 were overexpressed in BMDMs after transfected with *Irf7* gene. (**B**) Western blot results showed that IRF7 were knocked down in BMDMs after transfected with *Irf7* siRNA.

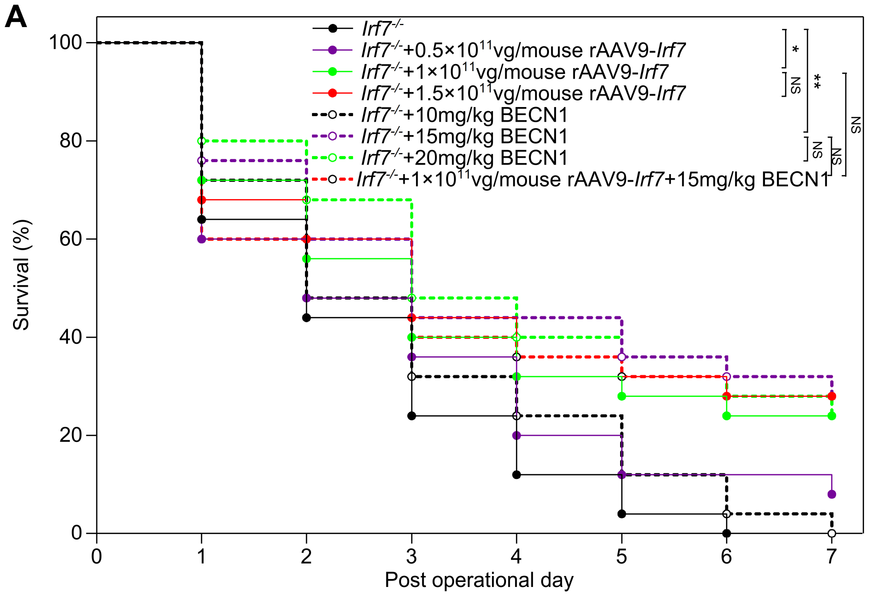

Supplemental Figure 9. Recombinant AAV9-*Irf7* protected septic mice via autophagy. (**A**) Within a certain dose range, increasing rAAV9-*Irf7* or BECN1 did not improve the survival rate of septic mice. Survival rates of mice were determined until 7 days after CLP (n = 25, log rank test). NS (not significant) $P \geq 0.05$, * $P < 0.05$, ** $P < 0.01$.

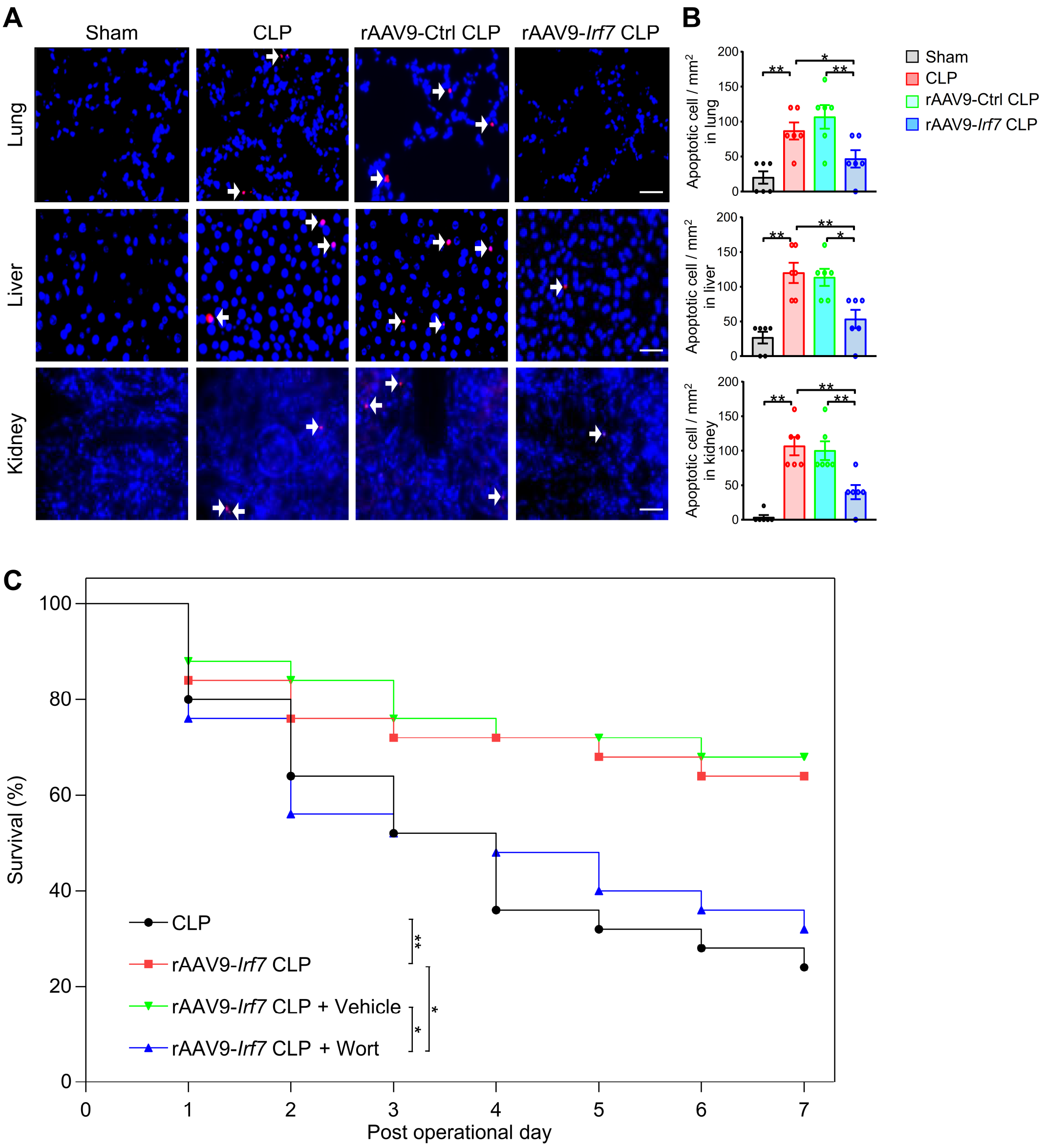

Supplemental Figure 10. Recombinant AAV9-*Irf7* protected WT mice subjected to CLP via autophagy. (**A, B**) Recombinant AAV9-*Irf7* decreased the number of apoptotic cells in the organs of WT mice subjected to CLP. The representative pictures of TUNEL staining (**A**), and the corresponding apoptotic cell quantification (**B**) in lung, liver and kidney. White arrow, TUNEL-positive cells. Scare bar, 25 μm. Error bars, ±SEM. One-way ANOVA with Bonferroni's correction. (**C**) Wort, an autophagy inhibitor, inhibited the protective effect of rAAV9-*Irf7* on WT mice underwent CLP. Survival rates of mice were determined until 7 days after CLP (n = 25, log rank test). * $P < 0.05$, ** $P < 0.01$.

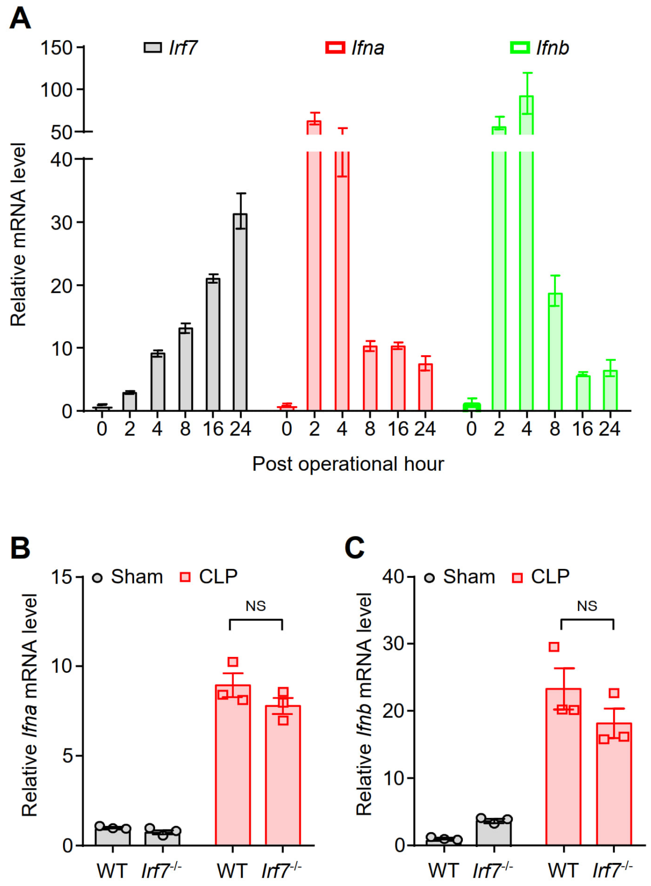

Supplemental Figure 11. IRF7 was not required for the expression of *Ifna* or *Ifnb* during polymicrobial sepsis. (**A**) Relative mRNA level of *Irf7*, *Ifna* and *Ifnb* in peritoneal cells isolated from WT mice underwent CLP for different time. n = 3. Relative mRNA level of (**B**) *Ifna* and (**C**) *Ifnb* in peritoneal cells from WT or *Irf7*$^{-/-}$ mice underwent CLP for 16 hours. Data represent the mean ± SEM. NS, $P \geq 0.05$, one-way ANOVA.

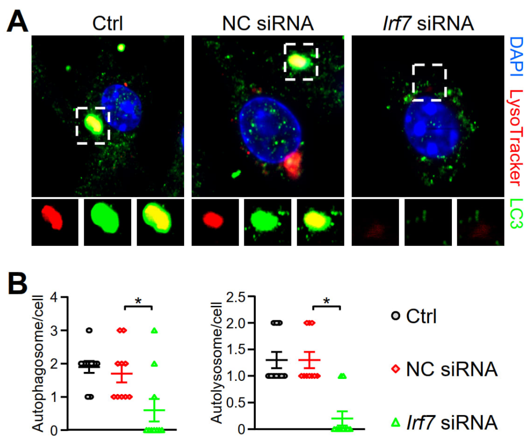

Supplemental Figure 12. *Irf7* knockdown inhibited autophagosome formation and autolysosome maturation. (**A**, **B**) BMDMs treated with LPS were loaded with LysoTracker, and further stained with LC3 and DAPI. (**A**) Representative fluorescent micrograph. (**B**) Autophagosome (LC3 positive puncta) number per cell with SEM (left panel), and autolysosome (LC3 and LysoTracker dual-positive puncta) number per cell with SEM (middle panel). Data represent the mean ± SEM. * $P < 0.05$, one-way ANOVA.

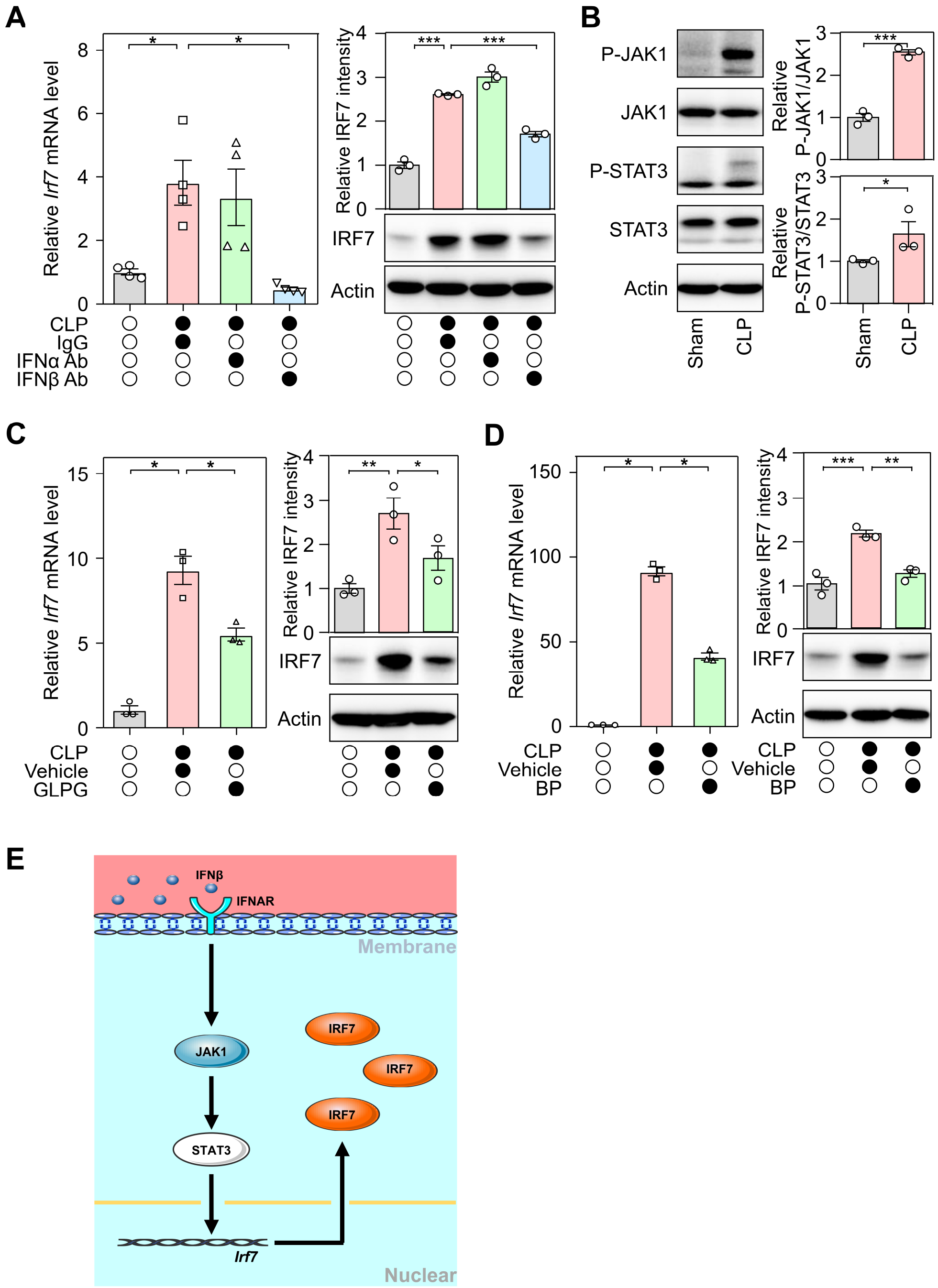

Supplemental Figure 13. IRF7 might be triggered by the IFNβ-JAK1-STAT3 pathway. (**A**) IFNβ neutralizing antibody (IFNβ Ab) inhibited IRF7 expression. *Irf7* mRNA (left panel) or IRF7 protein (right panel) levels in peritoneal cells were determined by RT-qPCR or Western blot assay. (**B**) JAK1-STAT3 pathway were activated after CLP. (**C**) GLPG0634 (GLPG), a selective JAK1 inhibitor, inhibited IRF7 expression in peritoneal cells. (**D**) BP-1-102 (BP), a selective STAT3 inhibitor, inhibited IRF7 expression in peritoneal cells. (**E**) The potential mechanism that controlled IRF7 expression during sepsis. Data represent the mean ± SEM. * $P < 0.05$, ** $P <$

0.01, *** P < 0.001, one-way ANOVA.

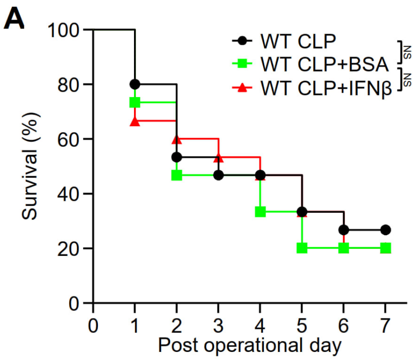

Supplemental Figure 14. IFNβ could not augment survival rates of septic mice. (**A**)Survival rates of mice were determined until 7 days after CLP**.** *n* = 15. NS, $P \geq 0.05$, log rank test.

Supplemental Table 1. Primer sequences for qPCR.

Gene name	Primer	Sequence
Irf7	Forward primer	5'-CAGGGGATCCAGTTGATCC-3'
	Reverse primer	5'-GAGCATTGCTGAGGCTCAC-3'
Ifnα	Forward primer	5'-CCTGTGTGATGCAGGAACC-3'
	Reverse primer	5'-TCACCTCCCAGGCACTGA-3'
Ifnβ	Forward primer	5'-ATGAGTGGTGGTTGCAGGC-3'
	Reverse primer	5'-TGACCTTTCAAATGCAGTAGATTCA-3'
Atg10	Forward primer	5'-TTCTGAAGTGACGAGACCTGC-3'
	Reverse primer	5'-GCCTCGGCTTATAGCACTCA-3'
Atg3	Forward primer	5'-GATGGTGATGGGGGATGGGTA-3'
	Reverse primer	5'-TTCCTCGTCTTCTTCATCACAC-3'
Lc3b	Forward primer	5'-CCCACCAAGATCCCAGTGATT-3'
	Reverse primer	5'-CTGCAAGCGCCGTCTGATTA-3'
Atg9a	Forward primer	5'-GTGCCAGGATTCAGGAAAATGG-3'
	Reverse primer	5'-GCAGTATGGAAGGGCAGACA-3'
Rab7	Forward primer	5'-TGGTGGACGACAGACTTGTT-3'
	Reverse primer	5'-TGAAAGTGTTGGGGGCAGTC-3'
Rab8a	Forward primer	5'-GTAGGGAAGACCTGTGTCCTG-3'
	Reverse primer	5'-CGTGATTGTCCGAAACCGC-3'
Lamp2	Forward primer	5' CATCGTGCTTTCCTACAACACT-3'
	Reverse primer	5' TCTGAACGACAGGAGTCAGGT-3'
Ctsl	Forward primer	5'-TCGGTGACATGACCAATGAGG-3'
	Reverse primer	5'-CACTGGCCCTGGTTCTTCAC-3'
Ctsb	Forward primer	5'-GGAAGGGTTGCGTTCGGTG-3'
	Reverse primer	5'-CACTGCCCCAAATGCCCAAC-3'
Ctse	Forward primer	5'-GACATCAGTCCCTTCGGAAGA-3'
	Reverse primer	5'-AGGGGTTCATTGACACTCGAATA-3'
Ctsd	Forward primer	5'-TCAAAAACTACCTGGATGCCCA-3'
	Reverse primer	5'-ACCCAGCAGGCTATGTCAAG-3'
Atg7	Forward primer	5'-GCTTTTGACATGAGTGCCTCC-3'
	Reverse primer	5'-CCAGATCTCATTTGCTGACTGC-3'
Actin	Forward primer	5'-GGCTGTATTCCCCTCCATCG-3'
	Reverse primer	5'-CCAGTTGGTAACAATGCCATGT-3'
16S rDNA	Forward primer	5'-GTGSTGCAYGGYTGTCGTCA-3'
	Reverse primer	5'-ACGTCRTCCMCACCTTCCTC-3'

Supplemental Table 2. IRF7-binding genes.

Gene ID	Symbol	Gene ID	Symbol
ENSMUSG00000000167	*Pih1d2*	ENSMUSG00000008496	*Pou2f2*
ENSMUSG00000000253	*Gmpr*	ENSMUSG00000008683	*Rps15a*
ENSMUSG00000000489	*Pdgfb*	ENSMUSG00000008855	*Hdac5*
ENSMUSG00000000579	*Dynlt1c*	ENSMUSG00000010406	*Mrpl52*
ENSMUSG00000000682	*Cd52*	ENSMUSG00000011114	*Tbrg1*
ENSMUSG00000000791	*Il12rb1*	ENSMUSG00000011267	*Zfp296*
ENSMUSG00000000982	*Ccl3*	ENSMUSG00000013236	*Ptprs*
ENSMUSG00000001053	*N4bp3*	ENSMUSG00000013662	*Atad1*
ENSMUSG00000001054	*Rmnd5b*	ENSMUSG00000013974	*Mcemp1*
ENSMUSG00000001089	*Luzp1*	ENSMUSG00000014164	*Klhl3*
ENSMUSG00000001313	*Rnd2*	ENSMUSG00000014353	*Tmem87b*
ENSMUSG00000001473	*Tubb6*	ENSMUSG00000014606	*Slc25a11*
ENSMUSG00000001627	*Ifrd1*	ENSMUSG00000014778	*Fhod1*
ENSMUSG00000001847	*Rac1*	ENSMUSG00000015112	*Slc25a13*
ENSMUSG00000002083	*Bbc3*	ENSMUSG00000015312	*Gadd45b*
ENSMUSG00000002147	*Stat6*	ENSMUSG00000015468	*Notch4*
ENSMUSG00000002222	*Rmnd5a*	ENSMUSG00000015656	*Hspa8*
ENSMUSG00000002228	*Ppm1j*	ENSMUSG00000015837	*Sqstm1*
ENSMUSG00000002847	*Pla1a*	ENSMUSG00000015932	*Dstn*
ENSMUSG00000002997	*Prkar2b*	ENSMUSG00000016087	*Fli1*
ENSMUSG00000003206	*Ebi3*	ENSMUSG00000016256	*Ctsz*
ENSMUSG00000003283	*Hck*	ENSMUSG00000016495	*Plgrkt*
ENSMUSG00000003363	*Pld3*	ENSMUSG00000016520	*Lnx2*
ENSMUSG00000003541	*Ier3*	ENSMUSG00000017002	*Slpi*
ENSMUSG00000003549	*Ercc1*	ENSMUSG00000017009	*Sdc4*
ENSMUSG00000003721	*Insig2*	ENSMUSG00000017057	*Il13ra1*
ENSMUSG00000004151	*Etv1*	ENSMUSG00000017386	*Traf4*
ENSMUSG00000004285	*Atp6v1f*	ENSMUSG00000017774	*Myo1c*
ENSMUSG00000004371	*Il11*	ENSMUSG00000017776	*Crk*
ENSMUSG00000004451	*Ralb*	ENSMUSG00000018347	*Zkscan6*
ENSMUSG00000004552	*Ctse*	ENSMUSG00000018476	*Kdm6b*
ENSMUSG00000004562	*Arhgef40*	ENSMUSG00000018570	*2810408A11Rik*
ENSMUSG00000004609	*Cd33*	ENSMUSG00000018648	*Dusp14*
ENSMUSG00000004798	*Ulk2*	ENSMUSG00000018736	*Ndel1*
ENSMUSG00000004864	*Mapk13*	ENSMUSG00000018740	*Slc25a35*
ENSMUSG00000004880	*Lbr*	ENSMUSG00000018899	*Irf1*
ENSMUSG00000004936	*Map2k1*	ENSMUSG00000018930	*Ccl4*
ENSMUSG00000005125	*Ndrg1*	ENSMUSG00000019054	*Fis1*
ENSMUSG00000005198	*Polr2a*	ENSMUSG00000019055	*Plod1*
ENSMUSG00000005225	*Plekha8*	ENSMUSG00000019373	*Cops3*
ENSMUSG00000005299	*Letm1*	ENSMUSG00000019564	*Arid3a*
ENSMUSG00000005514	*Por*	ENSMUSG00000019731	*Slc35e1*
ENSMUSG00000005824	*Tnfsf14*	ENSMUSG00000019810	*Fuca2*
ENSMUSG00000005873	*Reep5*	ENSMUSG00000019852	*Arfgef3*
ENSMUSG00000005936	*Kctd20*	ENSMUSG00000020009	*Ifngr1*
ENSMUSG00000005986	*Ankrd13d*	ENSMUSG00000020057	*Dram1*
ENSMUSG00000006221	*Hspb7*	ENSMUSG00000020120	*Plek*
ENSMUSG00000006310	*Zbtb32*	ENSMUSG00000020143	*Dock2*
ENSMUSG00000006362	*Cbfa2t3*	ENSMUSG00000020246	*Hcfc2*
ENSMUSG00000006445	*Epha2*	ENSMUSG00000020275	*Rel*
ENSMUSG00000006731	*B4galnt1*	ENSMUSG00000020303	*Stc2*
ENSMUSG00000006740	*Kif5b*	ENSMUSG00000020400	*Tnip1*
ENSMUSG00000006818	*Sod2*	ENSMUSG00000020422	*Tns3*
ENSMUSG00000006920	*Ezh1*	ENSMUSG00000020458	*Rtn4*
ENSMUSG00000006930	*Hap1*	ENSMUSG00000020463	*Ppp4r3b*
ENSMUSG00000007035	*Msh5*	ENSMUSG00000020477	*Mrps24*
ENSMUSG00000007659	*Bcl2l1*	ENSMUSG00000020485	*Supt4a*
ENSMUSG00000007817	*Zmiz1*	ENSMUSG00000020593	*Lpin1*
ENSMUSG00000007891	*Ctsd*	ENSMUSG00000020594	*Pum2*
ENSMUSG00000008129	*4930432K21Rik*	ENSMUSG00000020610	*Amz2*

Supplemental Table 2 (continued). IRF7-binding genes.

Gene ID	Symbol		Gene ID	Symbol
ENSMUSG00000020611	*Gna13*		ENSMUSG00000024180	*Tmem8*
ENSMUSG00000020651	*Slc26a4*		ENSMUSG00000024219	*Anks1*
ENSMUSG00000020669	*Sh3yl1*		ENSMUSG00000024228	*Nudt12*
ENSMUSG00000020715	*Ern1*		ENSMUSG00000024235	*Map3k8*
ENSMUSG00000020745	*Pafah1b1*		ENSMUSG00000024286	*Ccny*
ENSMUSG00000020901	*Pik3r5*		ENSMUSG00000024399	*Ltb*
ENSMUSG00000020941	*Map3k14*		ENSMUSG00000024401	*Tnf*
ENSMUSG00000021000	*Mia2*		ENSMUSG00000024451	*Arap3*
ENSMUSG00000021012	*Zc3h14*		ENSMUSG00000024483	*Ankhd1*
ENSMUSG00000021025	*Nfkbia*		ENSMUSG00000024507	*Hsd17b4*
ENSMUSG00000021051	*Ppp2r5e*		ENSMUSG00000024580	*Grpel2*
ENSMUSG00000021108	*Prkch*		ENSMUSG00000024621	*Csf1r*
ENSMUSG00000021127	*Zfp36l1*		ENSMUSG00000024659	*Anxa1*
ENSMUSG00000021133	*Susd6*		ENSMUSG00000024661	*Fth1*
ENSMUSG00000021171	*Esyt2*		ENSMUSG00000024669	*Cd5*
ENSMUSG00000021236	*Entpd5*		ENSMUSG00000024769	*Cdc42bpg*
ENSMUSG00000021240	*Abcd4*		ENSMUSG00000024789	*Jak2*
ENSMUSG00000021250	*Fos*		ENSMUSG00000024856	*Cdk2ap2*
ENSMUSG00000021281	*Tnfaip2*		ENSMUSG00000024900	*Cpt1a*
ENSMUSG00000021282	*Eif5*		ENSMUSG00000024912	*Fosl1*
ENSMUSG00000021384	*Susd3*		ENSMUSG00000024913	*Lrp5*
ENSMUSG00000021550	*2210016F16Rik*		ENSMUSG00000024968	*Rcor2*
ENSMUSG00000021591	*Glrx*		ENSMUSG00000024981	*Acsl5*
ENSMUSG00000021619	*Atg10*		ENSMUSG00000024986	*Hhex*
ENSMUSG00000021768	*Dusp13*		ENSMUSG00000025076	*Casp7*
ENSMUSG00000021785	*Ngly1*		ENSMUSG00000025077	*Dclre1a*
ENSMUSG00000021792	*Fam213a*		ENSMUSG00000025138	*Sirt7*
ENSMUSG00000021814	*Anxa7*		ENSMUSG00000025198	*Erlin1*
ENSMUSG00000021822	*Plau*		ENSMUSG00000025227	*Mfsd13a*
ENSMUSG00000021831	*Ero1l*		ENSMUSG00000025232	*Hexa*
ENSMUSG00000021939	*Ctsb*		ENSMUSG00000025383	*Il23a*
ENSMUSG00000021978	*Extl3*		ENSMUSG00000025407	*Gli1*
ENSMUSG00000022102	*Dok2*		ENSMUSG00000025429	*Pstpip2*
ENSMUSG00000022270	*Retreg1*		ENSMUSG00000025534	*Gusb*
ENSMUSG00000022353	*Mtss1*		ENSMUSG00000025602	*Zfp202*
ENSMUSG00000022367	*Has2*		ENSMUSG00000025958	*Creb1*
ENSMUSG00000022426	*Josd1*		ENSMUSG00000025979	*Mob4*
ENSMUSG00000022436	*Sh3bp1*		ENSMUSG00000026003	*Acadl*
ENSMUSG00000022453	*Naga*		ENSMUSG00000026029	*Casp8*
ENSMUSG00000022507	*1810013L24Rik*		ENSMUSG00000026103	*Gls*
ENSMUSG00000022533	*Atp13a3*		ENSMUSG00000026107	*Nabp1*
ENSMUSG00000022602	*Arc*		ENSMUSG00000026177	*Slc11a1*
ENSMUSG00000022656	*Nectin3*		ENSMUSG00000026197	*Zfand2b*
ENSMUSG00000022822	*Abcc5*		ENSMUSG00000026199	*Ankzf1*
ENSMUSG00000022952	*Runx1*		ENSMUSG00000026203	*Dnajb2*
ENSMUSG00000022965	*Ifngr2*		ENSMUSG00000026285	*Pdcd1*
ENSMUSG00000023021	*Cers5*		ENSMUSG00000026305	*Lrrfip1*
ENSMUSG00000023048	*Prr13*		ENSMUSG00000026317	*Cln8*
ENSMUSG00000023050	*Map3k12*		ENSMUSG00000026361	*Cdc73*
ENSMUSG00000023067	*Cdkn1a*		ENSMUSG00000026427	*Eif2d*
ENSMUSG00000023088	*Abcc1*		ENSMUSG00000026483	*Fam129a*
ENSMUSG00000023106	*Denr*		ENSMUSG00000026547	*Tagln2*
ENSMUSG00000023169	*Slc38a1*		ENSMUSG00000026563	*Tada1*
ENSMUSG00000023232	*Serinc2*		ENSMUSG00000026600	*Soat1*
ENSMUSG00000023944	*Hsp90ab1*		ENSMUSG00000026627	*Tmem206*
ENSMUSG00000023992	*Trem2*		ENSMUSG00000026628	*Atf3*
ENSMUSG00000024098	*Twsg1*		ENSMUSG00000026640	*Plxna2*
ENSMUSG00000024142	*Mlst8*		ENSMUSG00000026656	*Fcgr2b*
ENSMUSG00000024143	*Rhoq*		ENSMUSG00000026688	*Mgst3*
ENSMUSG00000024164	*C3*		ENSMUSG00000026701	*Prdx6*

Supplemental Table 2 (continued). IRF7-binding genes.

Gene ID	Symbol		Gene ID	Symbol
ENSMUSG00000026718	*Stam*		ENSMUSG00000028967	*Errfi1*
ENSMUSG00000026822	*Lcn2*		ENSMUSG00000028982	*Slc25a33*
ENSMUSG00000026875	*Traf1*		ENSMUSG00000029063	*Nadk*
ENSMUSG00000026893	*Gca*		ENSMUSG00000029098	*Acox3*
ENSMUSG00000026922	*Agpat2*		ENSMUSG00000029135	*Fosl2*
ENSMUSG00000026939	*Tmem141*		ENSMUSG00000029178	*Klf3*
ENSMUSG00000026984	*Il1f6*		ENSMUSG00000029207	*Apbb2*
ENSMUSG00000026994	*Galnt3*		ENSMUSG00000029217	*Tec*
ENSMUSG00000027035	*Cers6*		ENSMUSG00000029313	*Aff1*
ENSMUSG00000027068	*Dhrs9*		ENSMUSG00000029314	*Gpat3*
ENSMUSG00000027099	*Mtx2*		ENSMUSG00000029330	*Cds1*
ENSMUSG00000027111	*Itga6*		ENSMUSG00000029344	*Tpst2*
ENSMUSG00000027171	*Prrg4*		ENSMUSG00000029384	*2010109A12Rik*
ENSMUSG00000027203	*Dut*		ENSMUSG00000029397	*Rchy1*
ENSMUSG00000027215	*Cd82*		ENSMUSG00000029403	*Cdkl2*
ENSMUSG00000027322	*Siglec1*		ENSMUSG00000029484	*Anxa3*
ENSMUSG00000027342	*Pcna*		ENSMUSG00000029534	*St7*
ENSMUSG00000027367	*Stard7*		ENSMUSG00000029552	*Tes*
ENSMUSG00000027368	*Dusp2*		ENSMUSG00000029580	*Actb*
ENSMUSG00000027381	*Bcl2l11*		ENSMUSG00000029642	*Polr1d*
ENSMUSG00000027387	*Zc3h8*		ENSMUSG00000029681	*Bcl7b*
ENSMUSG00000027430	*Dtd1*		ENSMUSG00000029708	*Gcc1*
ENSMUSG00000027465	*Tbc1d20*		ENSMUSG00000029720	*Gm20605*
ENSMUSG00000027487	*Cdk5rap1*		ENSMUSG00000029723	*Tsc22d4*
ENSMUSG00000027506	*Tpd52*		ENSMUSG00000029735	*Tpk1*
ENSMUSG00000027519	*Rab22a*		ENSMUSG00000029771	*Irf5*
ENSMUSG00000027544	*Nfatc2*		ENSMUSG00000029860	*Zyx*
ENSMUSG00000027639	*Samhd1*		ENSMUSG00000029920	*Smarcad1*
ENSMUSG00000027660	*Skil*		ENSMUSG00000030007	*Cct7*
ENSMUSG00000027737	*Slc7a11*		ENSMUSG00000030042	*Pole4*
ENSMUSG00000027843	*Ptpn22*		ENSMUSG00000030103	*Bhlhe40*
ENSMUSG00000027907	*S100a11*		ENSMUSG00000030122	*Ptms*
ENSMUSG00000027940	*Tpm3*		ENSMUSG00000030208	*Emp1*
ENSMUSG00000027947	*Il6ra*		ENSMUSG00000030232	*Aebp2*
ENSMUSG00000027995	*Tlr2*		ENSMUSG00000030314	*Atg7*
ENSMUSG00000028042	*Zbtb7b*		ENSMUSG00000030403	*Vasp*
ENSMUSG00000028064	*Sema4a*		ENSMUSG00000030409	*Dmpk*
ENSMUSG00000028080	*Lrba*		ENSMUSG00000030557	*Mef2a*
ENSMUSG00000028089	*Chd1l*		ENSMUSG00000030560	*Ctsc*
ENSMUSG00000028121	*Bcar3*		ENSMUSG00000030583	*Sipa1l3*
ENSMUSG00000028152	*Tspan5*		ENSMUSG00000030662	*Ipo5*
ENSMUSG00000028229	*Rmdn1*		ENSMUSG00000030707	*Coro1a*
ENSMUSG00000028249	*Sdcbp*		ENSMUSG00000030717	*Nupr1*
ENSMUSG00000028271	*Gtf2b*		ENSMUSG00000030789	*Itgax*
ENSMUSG00000028393	*Alad*		ENSMUSG00000030801	*Kat8*
ENSMUSG00000028417	*Tal2*		ENSMUSG00000030847	*Bag3*
ENSMUSG00000028454	*Pigo*		ENSMUSG00000031015	*Swap70*
ENSMUSG00000028458	*Tesk1*		ENSMUSG00000031097	*Tnni2*
ENSMUSG00000028552	*Eps15*		ENSMUSG00000031146	*Plp2*
ENSMUSG00000028581	*Laptm5*		ENSMUSG00000031536	*Polb*
ENSMUSG00000028617	*Lrrc42*		ENSMUSG00000031557	*Plekha2*
ENSMUSG00000028673	*Fuca1*		ENSMUSG00000031604	*Msmo1*
ENSMUSG00000028680	*Plk3*		ENSMUSG00000031628	*Casp3*
ENSMUSG00000028751	*Pla2g2e*		ENSMUSG00000031659	*Adcy7*
ENSMUSG00000028793	*Rnf19b*		ENSMUSG00000031662	*Snx20*
ENSMUSG00000028803	*Nipal3*		ENSMUSG00000031731	*Ap1g1*
ENSMUSG00000028841	*Cnksr1*		ENSMUSG00000031762	*Mt2*
ENSMUSG00000028842	*Ago3*		ENSMUSG00000031827	*Cotl1*
ENSMUSG00000028874	*Fgr*		ENSMUSG00000031844	*Hsd17b2*
ENSMUSG00000028931	*Kcnab2*		ENSMUSG00000031904	*Slc7a6*

Supplemental Table 2 (continued). IRF7-binding genes.

Gene ID	Symbol		Gene ID	Symbol
ENSMUSG00000032046	*Abhd12*		ENSMUSG00000035293	*G2e3*
ENSMUSG00000032047	*Acat1*		ENSMUSG00000035325	*Sec31a*
ENSMUSG00000032066	*Bco2*		ENSMUSG00000035342	*Lzts2*
ENSMUSG00000032089	*Il10ra*		ENSMUSG00000035376	*Hacd2*
ENSMUSG00000032204	*Aqp9*		ENSMUSG00000035441	*Myo1d*
ENSMUSG00000032228	*Tcf12*		ENSMUSG00000035513	*Ntng2*
ENSMUSG00000032254	*Kif23*		ENSMUSG00000035621	*Midn*
ENSMUSG00000032294	*Pkm*		ENSMUSG00000035673	*Sbno2*
ENSMUSG00000032300	*1700017B05Rik*		ENSMUSG00000035828	*Pim3*
ENSMUSG00000032366	*Tpm1*		ENSMUSG00000035898	*Uba6*
ENSMUSG00000032370	*Lactb*		ENSMUSG00000035914	*Cd276*
ENSMUSG00000032401	*Lctl*		ENSMUSG00000036057	*Ptpn23*
ENSMUSG00000032407	*U2surp*		ENSMUSG00000036067	*Slc2a6*
ENSMUSG00000032412	*Atp1b3*		ENSMUSG00000036181	*Hist1h1c*
ENSMUSG00000032422	*Snx14*		ENSMUSG00000036323	*Srp72*
ENSMUSG00000032440	*Tgfbr2*		ENSMUSG00000036452	*Arhgap26*
ENSMUSG00000032487	*Ptgs2*		ENSMUSG00000036480	*Prss56*
ENSMUSG00000032501	*Trib1*		ENSMUSG00000036526	*Card11*
ENSMUSG00000032578	*Cish*		ENSMUSG00000036533	*Cdc42ep3*
ENSMUSG00000032691	*Nlrp3*		ENSMUSG00000036620	*Mgat4b*
ENSMUSG00000032842	*Abcc10*		ENSMUSG00000037012	*Hk1*
ENSMUSG00000032855	*Pkd1*		ENSMUSG00000037295	*Ldlrap1*
ENSMUSG00000032870	*Smap2*		ENSMUSG00000037344	*Slc12a9*
ENSMUSG00000033004	*Mycbp2*		ENSMUSG00000037405	*Icam1*
ENSMUSG00000033014	*Trim33*		ENSMUSG00000037466	*Tedc1*
ENSMUSG00000033016	*Nfatc1*		ENSMUSG00000037573	*Tob1*
ENSMUSG00000033068	*Entpd6*		ENSMUSG00000037742	*Eef1a1*
ENSMUSG00000033124	*Atg9a*		ENSMUSG00000037815	*Ctnna1*
ENSMUSG00000033161	*Atp1a1*		ENSMUSG00000037868	*Egr2*
ENSMUSG00000033192	*Lpcat2*		ENSMUSG00000037966	*Ninj1*
ENSMUSG00000033220	*Rac2*		ENSMUSG00000037995	*Igsf9*
ENSMUSG00000033416	*Gucd1*		ENSMUSG00000038025	*Phf2*
ENSMUSG00000033629	*Hacd3*		ENSMUSG00000038037	*Socs1*
ENSMUSG00000033705	*Stard9*		ENSMUSG00000038067	*Csf3*
ENSMUSG00000033767	*Tmem131l*		ENSMUSG00000038151	*Prdm1*
ENSMUSG00000033863	*Klf9*		ENSMUSG00000038174	*Fam126b*
ENSMUSG00000033871	*Ppargc1b*		ENSMUSG00000038366	*Lasp1*
ENSMUSG00000034041	*Lyl1*		ENSMUSG00000038372	*Gmds*
ENSMUSG00000034075	*Zdhhc5*		ENSMUSG00000038416	*Cdc16*
ENSMUSG00000034101	*Ctnnd1*		ENSMUSG00000038425	*Poli*
ENSMUSG00000034116	*Vav1*		ENSMUSG00000038437	*Mllt6*
ENSMUSG00000034118	*Tpst1*		ENSMUSG00000038517	*Tbkbp1*
ENSMUSG00000034247	*Plekhm1*		ENSMUSG00000038563	*Efl1*
ENSMUSG00000034271	*Jdp2*		ENSMUSG00000038605	*Samd10*
ENSMUSG00000034320	*Slc26a2*		ENSMUSG00000038607	*Gng10*
ENSMUSG00000034557	*Zfyve9*		ENSMUSG00000038612	*Mcl1*
ENSMUSG00000034602	*Mon2*		ENSMUSG00000038650	*Rnh1*
ENSMUSG00000034652	*Cd300a*		ENSMUSG00000038732	*Mboat1*
ENSMUSG00000034706	*Dnaic2*		ENSMUSG00000038844	*Kif16b*
ENSMUSG00000034708	*Grn*		ENSMUSG00000038900	*Rpl12*
ENSMUSG00000034709	*Ppp1r21*		ENSMUSG00000038990	*Cables2*
ENSMUSG00000034801	*Sos2*		ENSMUSG00000039005	*Tlr4*
ENSMUSG00000034854	*Mfsd12*		ENSMUSG00000039048	*Foxred1*
ENSMUSG00000034858	*Fam214a*		ENSMUSG00000039157	*Fam102a*
ENSMUSG00000034917	*Tjp3*		ENSMUSG00000039191	*Rbpj*
ENSMUSG00000034947	*Tmem106a*		ENSMUSG00000039236	*Isg20*
ENSMUSG00000035139	*Secisbp2*		ENSMUSG00000039285	*Azi2*
ENSMUSG00000035158	*Mitf*		ENSMUSG00000039616	*Mocos*
ENSMUSG00000035161	*Ints6*		ENSMUSG00000039701	*Usp53*
ENSMUSG00000035164	*Zc3h12c*		ENSMUSG00000039747	*Orai2*

Supplemental Table 2 (continued). IRF7-binding genes.

Gene ID	Symbol		Gene ID	Symbol
ENSMUSG00000039810	*Zc3h10*		ENSMUSG00000045322	*Tlr9*
ENSMUSG00000039813	*Tbc1d2*		ENSMUSG00000045374	*Wdr81*
ENSMUSG00000039914	*Coq10a*		ENSMUSG00000045404	*Kcnk13*
ENSMUSG00000039942	*Ptger4*		ENSMUSG00000045482	*Trrap*
ENSMUSG00000040010	*Slc7a5*		ENSMUSG00000045795	*Whamm*
ENSMUSG00000040111	*Gramd1b*		ENSMUSG00000045867	*Cradd*
ENSMUSG00000040188	*Scamp2*		ENSMUSG00000045934	*Mtmr11*
ENSMUSG00000040272	*Accs*		ENSMUSG00000045973	*Slc25a51*
ENSMUSG00000040274	*Cdk6*		ENSMUSG00000046079	*Lrrc8d*
ENSMUSG00000040297	*Suco*		ENSMUSG00000046179	*E2f8*
ENSMUSG00000040389	*Wdr47*		ENSMUSG00000046223	*Plaur*
ENSMUSG00000040433	*Zbtb38*		ENSMUSG00000046410	*Kcnk6*
ENSMUSG00000040472	*Rabggta*		ENSMUSG00000046456	*Tmem150b*
ENSMUSG00000040552	*C3ar1*		ENSMUSG00000046546	*Fam43a*
ENSMUSG00000040701	*Ap1g2*		ENSMUSG00000046718	*Bst2*
ENSMUSG00000040713	*Creg1*		ENSMUSG00000046727	*Cystm1*
ENSMUSG00000040751	*Lat2*		ENSMUSG00000046731	*Kctd11*
ENSMUSG00000040848	*Sft2d2*		ENSMUSG00000046805	*Mpeg1*
ENSMUSG00000040964	*Arhgef10l*		ENSMUSG00000047034	*Ankrd33*
ENSMUSG00000041132	*N4bp2l1*		ENSMUSG00000047048	*Olfr432*
ENSMUSG00000041143	*Tmco4*		ENSMUSG00000047067	*Dusp28*
ENSMUSG00000041193	*Pla2g5*		ENSMUSG00000047126	*Cltc*
ENSMUSG00000041362	*Shtn1*		ENSMUSG00000047221	*Fam185a*
ENSMUSG00000041515	*Irf8*		ENSMUSG00000047250	*Ptgs1*
ENSMUSG00000041607	*Mbp*		ENSMUSG00000047412	*Zbtb44*
ENSMUSG00000041625	*Ggact*		ENSMUSG00000047547	*Cltb*
ENSMUSG00000041754	*Trem3*		ENSMUSG00000047671	*Tctex1d4*
ENSMUSG00000041763	*Tpp2*		ENSMUSG00000047767	*Atg16l2*
ENSMUSG00000041773	*Enc1*		ENSMUSG00000047798	*Cd300lf*
ENSMUSG00000041920	*Slc16a6*		ENSMUSG00000047804	*Akap10*
ENSMUSG00000041957	*Pkp2*		ENSMUSG00000047810	*Ccdc88b*
ENSMUSG00000041959	*S100a10*		ENSMUSG00000047898	*Ccr4*
ENSMUSG00000042303	*Sgsm3*		ENSMUSG00000048230	*Fbxo43*
ENSMUSG00000042363	*Lgalsl*		ENSMUSG00000048277	*Syngr2*
ENSMUSG00000042404	*Dennd4b*		ENSMUSG00000048661	*Lemd3*
ENSMUSG00000042410	*Agps*		ENSMUSG00000048701	*Ccdc6*
ENSMUSG00000042476	*Abcb4*		ENSMUSG00000048779	*P2ry6*
ENSMUSG00000042507	*Elmsan1*		ENSMUSG00000048807	*Slc35e4*
ENSMUSG00000042613	*Pbxip1*		ENSMUSG00000048832	*Vps37c*
ENSMUSG00000042626	*Shc1*		ENSMUSG00000048911	*Rnf24*
ENSMUSG00000042742	*Bmt2*		ENSMUSG00000049086	*Bmyc*
ENSMUSG00000042745	*Id1*		ENSMUSG00000049313	*Sorl1*
ENSMUSG00000042759	*Apobr*		ENSMUSG00000049411	*Tmem241*
ENSMUSG00000042826	*Fgf11*		ENSMUSG00000049562	*Ap5b1*
ENSMUSG00000043131	*Mob1a*		ENSMUSG00000049807	*Arhgap23*
ENSMUSG00000043157	*Arl11*		ENSMUSG00000049892	*Rasd1*
ENSMUSG00000043257	*Pigv*		ENSMUSG00000049932	*H2afx*
ENSMUSG00000043279	*Trim56*		ENSMUSG00000049988	*Lrrc25*
ENSMUSG00000043421	*Hilpda*		ENSMUSG00000050022	*Amz1*
ENSMUSG00000043895	*S1pr2*		ENSMUSG00000050335	*Lgals3*
ENSMUSG00000043953	*Ccrl2*		ENSMUSG00000050468	*Astl*
ENSMUSG00000044092	*C130050O18Rik*		ENSMUSG00000050732	*Vamp8*
ENSMUSG00000044313	*Mab21l3*		ENSMUSG00000050821	*Fam131a*
ENSMUSG00000044456	*Rin3*		ENSMUSG00000051113	*Fam71e1*
ENSMUSG00000044641	*Pard6b*		ENSMUSG00000051335	*Gfod1*
ENSMUSG00000044701	*Il27*		ENSMUSG00000051506	*Wdfy4*
ENSMUSG00000044709	*Gemin7*		ENSMUSG00000051627	*Hist1h1e*
ENSMUSG00000044864	*Ankrd50*		ENSMUSG00000051650	*B3gnt2*
ENSMUSG00000045027	*Prss22*		ENSMUSG00000051748	*Wfdc21*
ENSMUSG00000045193	*Cirbp*		ENSMUSG00000052298	*Cdc42se2*

Supplemental Table 2 (continued). IRF7-binding genes.

Gene ID	Symbol		Gene ID	Symbol
ENSMUSG00000052397	*Ezr*		ENSMUSG00000063506	*Arhgap22*
ENSMUSG00000052423	*B4galt3*		ENSMUSG00000063849	*Ppcdc*
ENSMUSG00000052435	*Cebpe*		ENSMUSG00000063851	*Rnf183*
ENSMUSG00000052560	*Cpne8*		ENSMUSG00000064090	*Vrk2*
ENSMUSG00000052688	*Rab7b*		ENSMUSG00000064288	*Hist1h4k*
ENSMUSG00000052749	*Trim30b*		ENSMUSG00000064289	*Tank*
ENSMUSG00000052837	*Junb*		ENSMUSG00000066026	*Dhrs3*
ENSMUSG00000053113	*Socs3*		ENSMUSG00000066306	*Numa1*
ENSMUSG00000053158	*Fes*		ENSMUSG00000066441	*Rdh11*
ENSMUSG00000053477	*Tcf4*		ENSMUSG00000066838	*Zfp772*
ENSMUSG00000053600	*Zfp472*		ENSMUSG00000067274	*Rplp0*
ENSMUSG00000053684	*BC048403*		ENSMUSG00000067455	*Hist1h4j*
ENSMUSG00000054027	*Nt5dc3*		ENSMUSG00000067787	*Blcap*
ENSMUSG00000054051	*Ercc6*		ENSMUSG00000067851	*Arfgef1*
ENSMUSG00000054065	*Pkp3*		ENSMUSG00000068206	*Pick1*
ENSMUSG00000054150	*Syne3*		ENSMUSG00000068699	*Flnc*
ENSMUSG00000054302	*Eapp*		ENSMUSG00000068798	*Rap1a*
ENSMUSG00000054428	*Atpif1*		ENSMUSG00000069266	*Hist1h4b*
ENSMUSG00000054619	*Mettl7a1*		ENSMUSG00000069273	*Hist1h3e*
ENSMUSG00000054702	*Ap1s3*		ENSMUSG00000069631	*Strada*
ENSMUSG00000054733	*Msra*		ENSMUSG00000069892	*9930111J21Rik2*
ENSMUSG00000054793	*Cadm4*		ENSMUSG00000070283	*Ndufaf3*
ENSMUSG00000054808	*Actn4*		ENSMUSG00000070327	*Rnf213*
ENSMUSG00000054894	*Atp5s*		ENSMUSG00000070462	*Tlnrd1*
ENSMUSG00000055447	*Cd47*		ENSMUSG00000070524	*Fcrlb*
ENSMUSG00000055805	*Fmnl1*		ENSMUSG00000070717	*Gm10300*
ENSMUSG00000055835	*Zfp1*		ENSMUSG00000071042	*Rasgrp3*
ENSMUSG00000055926	*Gm14137*		ENSMUSG00000071064	*Zfp827*
ENSMUSG00000055994	*Nod2*		ENSMUSG00000071076	*Jund*
ENSMUSG00000056267	*Cep70*		ENSMUSG00000071369	*Map3k5*
ENSMUSG00000056413	*Adap1*		ENSMUSG00000071516	*Hist1h2ai*
ENSMUSG00000056498	*Tmem154*		ENSMUSG00000071573	*Rnls*
ENSMUSG00000056501	*Cebpb*		ENSMUSG00000071637	*Cebpd*
ENSMUSG00000056515	*Rab31*		ENSMUSG00000071713	*Csf2rb*
ENSMUSG00000056749	*Nfil3*		ENSMUSG00000071715	*Ncf4*
ENSMUSG00000056888	*Glipr1*		ENSMUSG00000073412	*Lst1*
ENSMUSG00000056952	*Tatdn2*		ENSMUSG00000073631	*Gm10553*
ENSMUSG00000057649	*Brd9*		ENSMUSG00000074063	*Osgin1*
ENSMUSG00000057672	*Pkn1*		ENSMUSG00000074221	*Zfp568*
ENSMUSG00000058216	*Gstp3*		ENSMUSG00000074361	*C5ar2*
ENSMUSG00000058427	*Cxcl2*		ENSMUSG00000074403	*Hist2h3b*
ENSMUSG00000058755	*Osm*		ENSMUSG00000074417	*Gm14548*
ENSMUSG00000058773	*Hist1h1b*		ENSMUSG00000074623	*Gm826*
ENSMUSG00000059108	*Ifitm6*		ENSMUSG00000074802	*Gas2l3*
ENSMUSG00000059288	*Cdyl*		ENSMUSG00000074825	*Itpripl1*
ENSMUSG00000059456	*Ptk2b*		ENSMUSG00000074862	*BC025920*
ENSMUSG00000059923	*Grb2*		ENSMUSG00000074886	*Grk6*
ENSMUSG00000060093	*Hist1h4a*		ENSMUSG00000075284	*Wipf1*
ENSMUSG00000060639	*Hist1h4i*		ENSMUSG00000075486	*Commd6*
ENSMUSG00000060950	*Trmt61a*		ENSMUSG00000075703	*Selenoi*
ENSMUSG00000061028	*Clasrp*		ENSMUSG00000076618	*Ighj4*
ENSMUSG00000061132	*Blnk*		ENSMUSG00000078249	*Hmga1b*
ENSMUSG00000061482	*Hist1h4d*		ENSMUSG00000078515	*Ddi2*
ENSMUSG00000061755	*Bod1l*		ENSMUSG00000079016	*Gm11034*
ENSMUSG00000062031	*Pgghg*		ENSMUSG00000079550	*Mpp4*
ENSMUSG00000062300	*Nectin2*		ENSMUSG00000079794	*AC125149.2*
ENSMUSG00000062743	*Zfp677*		ENSMUSG00000086962	*Gm12248*
ENSMUSG00000063015	*Ccni*		ENSMUSG00000087610	*Gm16253*
ENSMUSG00000063193	*Cd300lb*		ENSMUSG00000089669	*Tnfsf13*
ENSMUSG00000063234	*Gpr84*		ENSMUSG00000090115	*Usp49*

Supplemental Table 2 (continued). IRF7-binding genes.

Gene ID	Symbol
ENSMUSG00000091405	*Hist2h4*
ENSMUSG00000093938	*Evi2b*
ENSMUSG00000094018	*S100a2*
ENSMUSG00000094028	*Ighd4-1*
ENSMUSG00000094127	*G530012D18Rik*
ENSMUSG00000095041	*AC149090.1*
ENSMUSG00000095450	*AC132444.4*
ENSMUSG00000095787	*AC133103.6*
ENSMUSG00000095891	*Gm10717*
ENSMUSG00000096201	*Gm10715*
ENSMUSG00000096736	*Gm17535*
ENSMUSG00000096764	*Gm21985*
ENSMUSG00000098112	*Bin2*
ENSMUSG00000099655	*2310034G01Rik*
ENSMUSG00000100210	*Hist1h3f*
ENSMUSG00000101972	*Hist1h3i*
ENSMUSG00000105827	*Hist2h2bb*
ENSMUSG00000108596	*Gm49368*
ENSMUSG00000108900	*Ccdc194*
