# Supplementary material for: IRF7 drives macrophages to kill bacteria and improves septic outcomes via autophagy
Source: JCI Insight. 2025 Nov 10;10(21):e189420. doi: 10.1172/jci.insight.189420 (PMC12643508; doi:10.1172/jci.insight.189420)

Unedited blot for Figure 4A

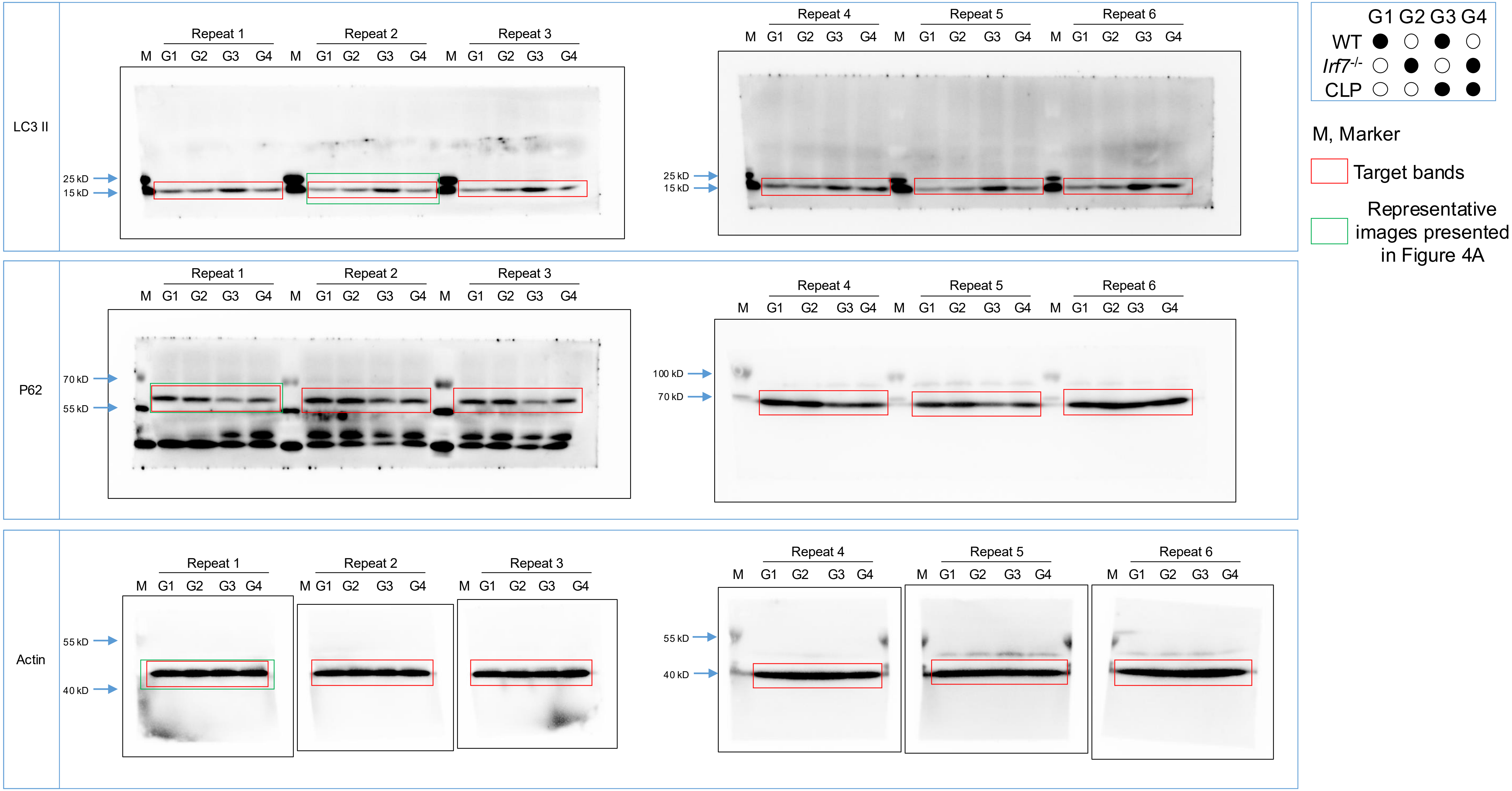

Unedited blot for Figure 4B and Supplemental Figure 5A

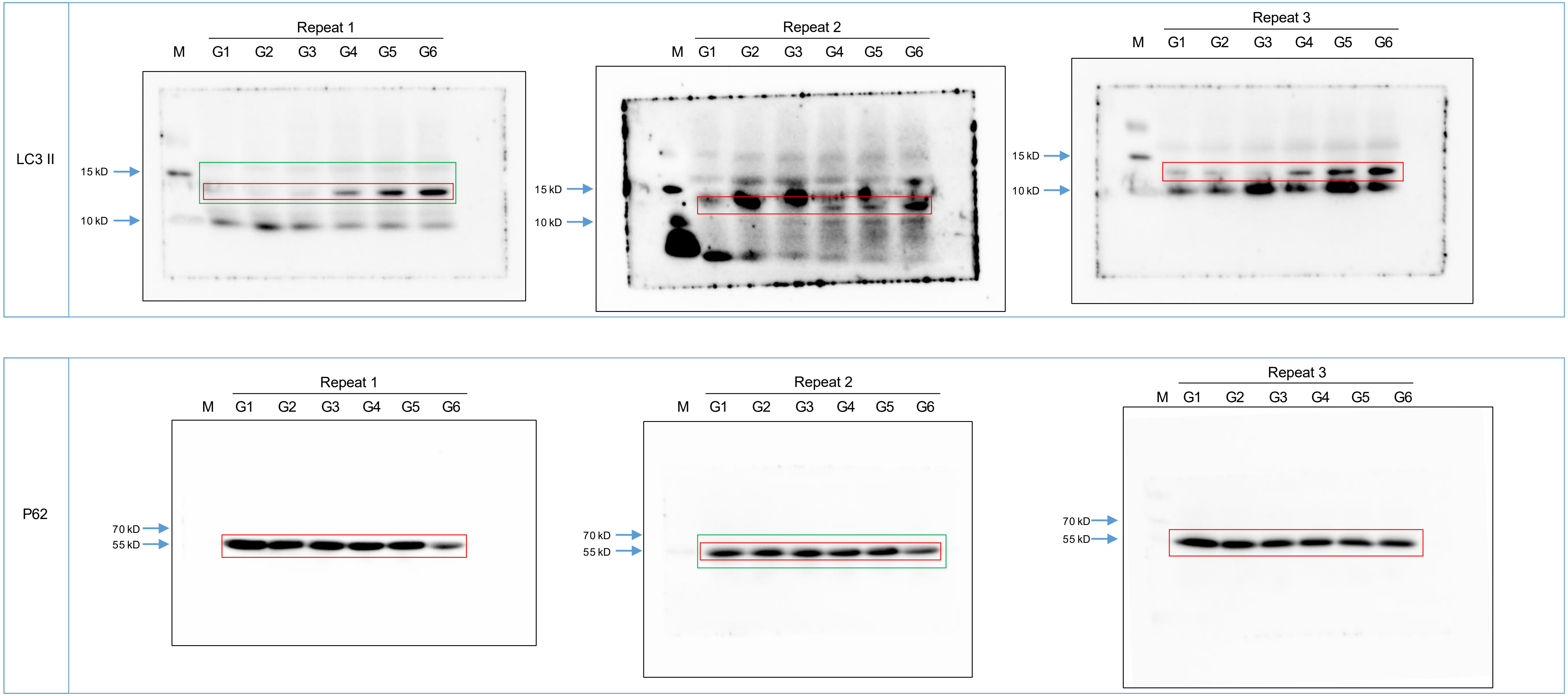

	G1	G2	G3	G4	G5	G6
Vector	○	●	○	○	●	○
Irf7	○	○	●	○	○	●
LPS	○	○	○	●	●	●

M, Marker

Target bands

Representative images presented in Figure 4B

Unedited blot for Figure 4B and Supplemental Figure 5A

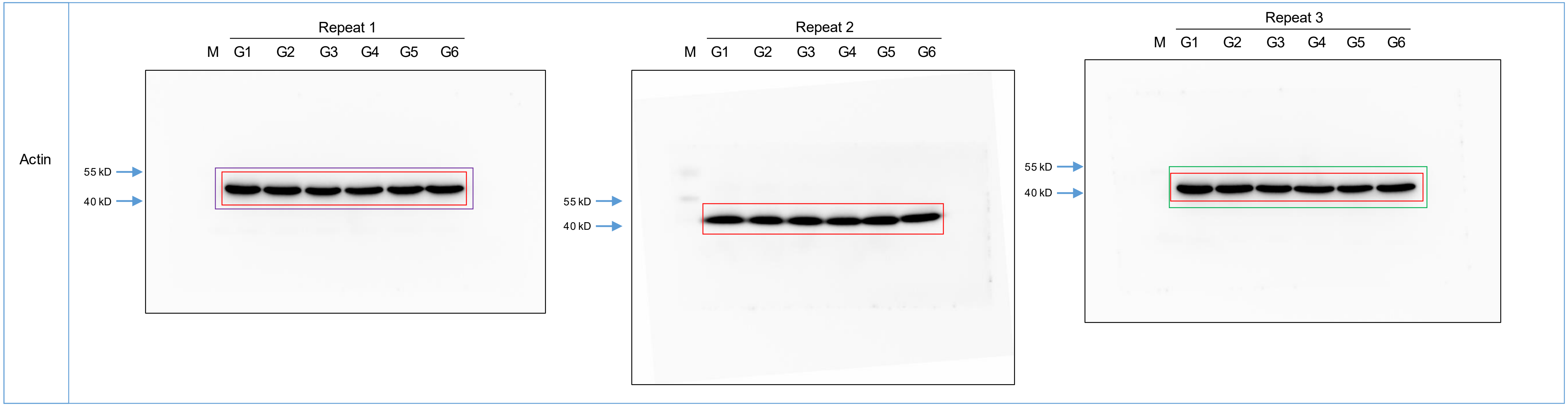

	G1	G2	G3	G4	G5	G6
Vector	○	●	○	○	●	○
Irf7	○	○	●	○	○	●
LPS	○	○	○	●	●	●

M, Marker

Red box: Target bands

Purple box: Representative images presented in Supplemental Figure 5A

Green box: Representative images presented in Figure 4B

Unedited blot for Figure 4B and Supplemental Figure 5A

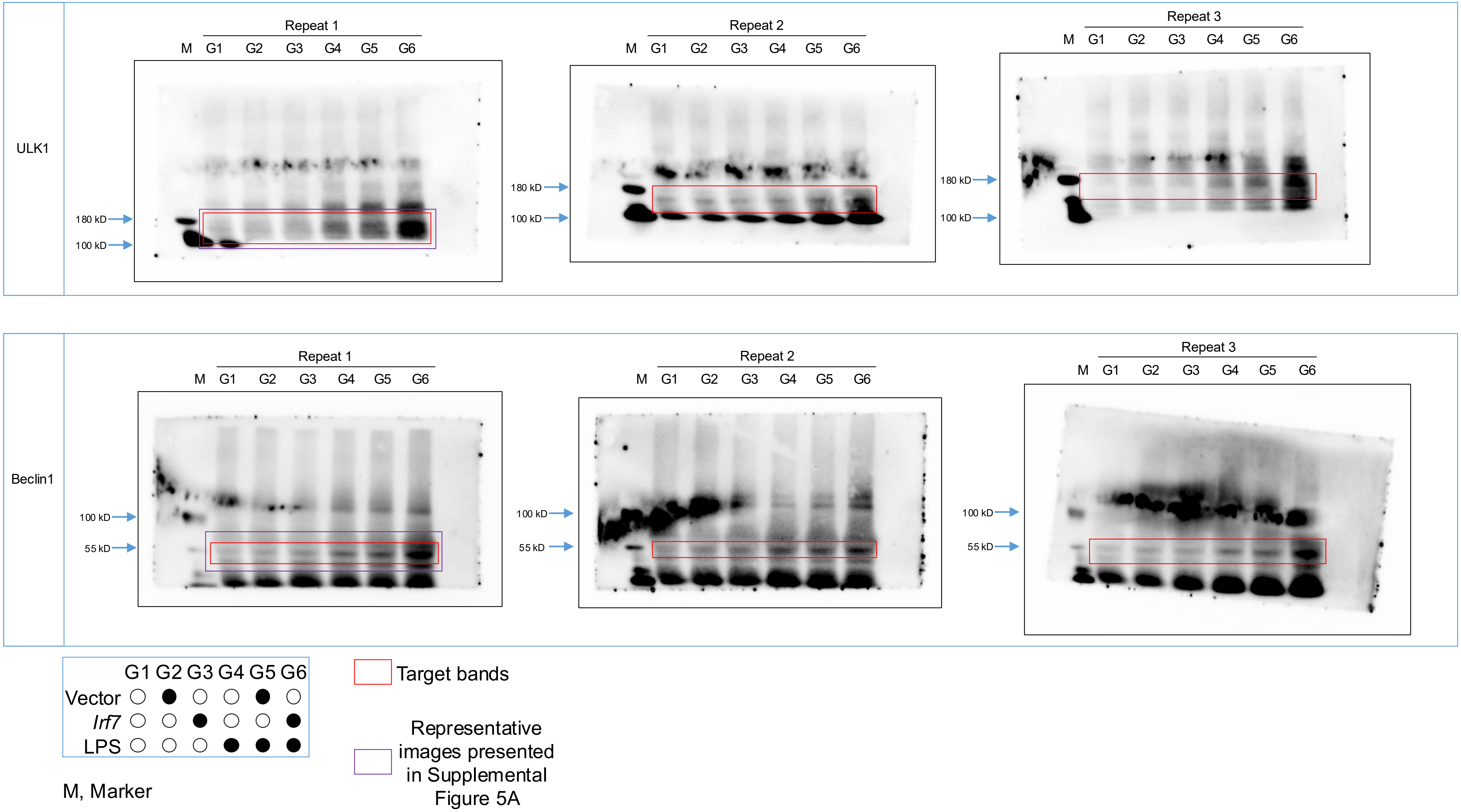

Unedited blot for Figure 4B and Supplemental Figure 5A

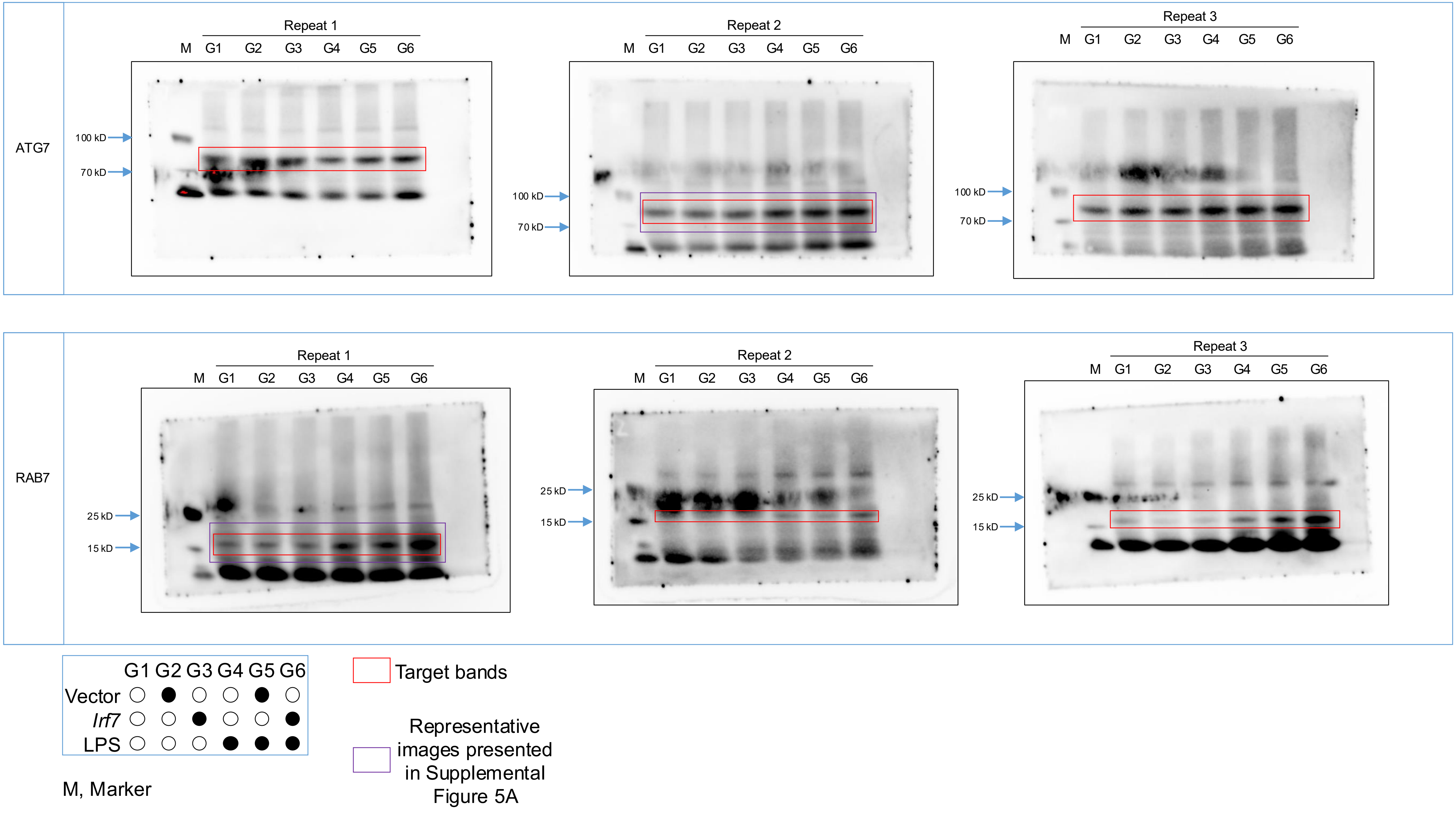

Unedited blot for Figure 4B and Supplemental Figure 5A

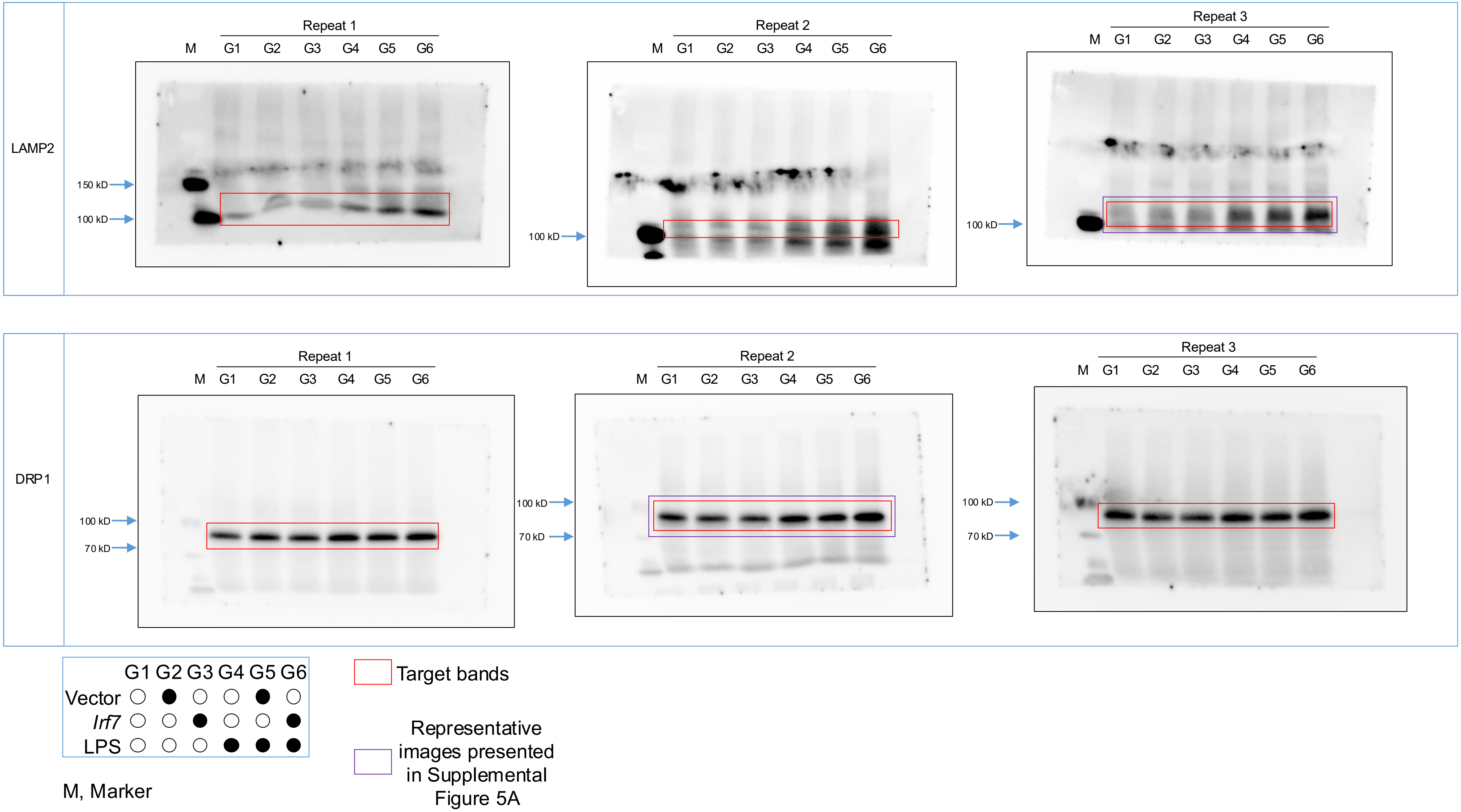

Unedited blot for Figure 4C and Supplemental Figure 5B

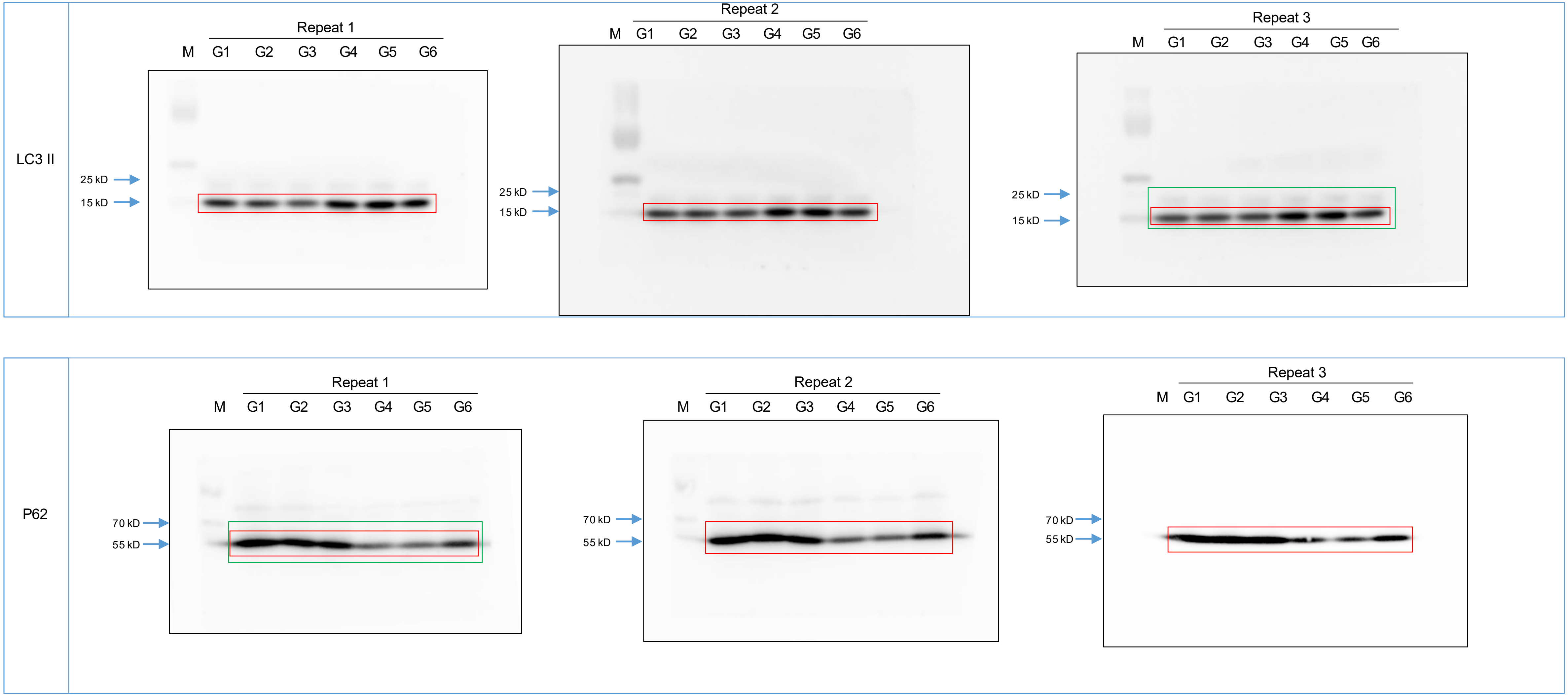

	G1	G2	G3	G4	G5	G6
NC siRNA	○	●	○	○	●	○
Irf7 siRNA	○	○	●	○	○	●
LPS	○	○	○	●	●	●

M, Marker

Red box: Target bands

Green box: Representative images presented in Figure 4C

Unedited blot for Figure 4C and Supplemental Figure 5B

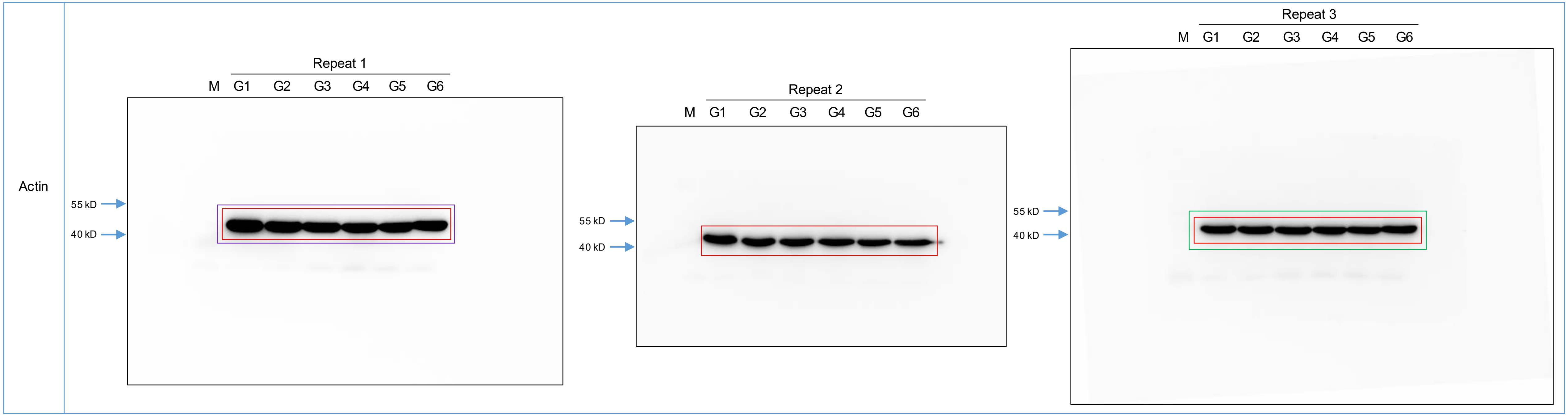

	G1	G2	G3	G4	G5	G6
NC siRNA	○	●	○	○	●	○
Irf7 siRNA	○	○	●	○	○	●
LPS	○	○	○	●	●	●

M, Marker

Red box: Target bands

Purple box: Representative images presented in Supplemental Figure 5B

Green box: Representative images presented in Figure 4C

Unedited blot for Figure 4C and Supplemental Figure 5B

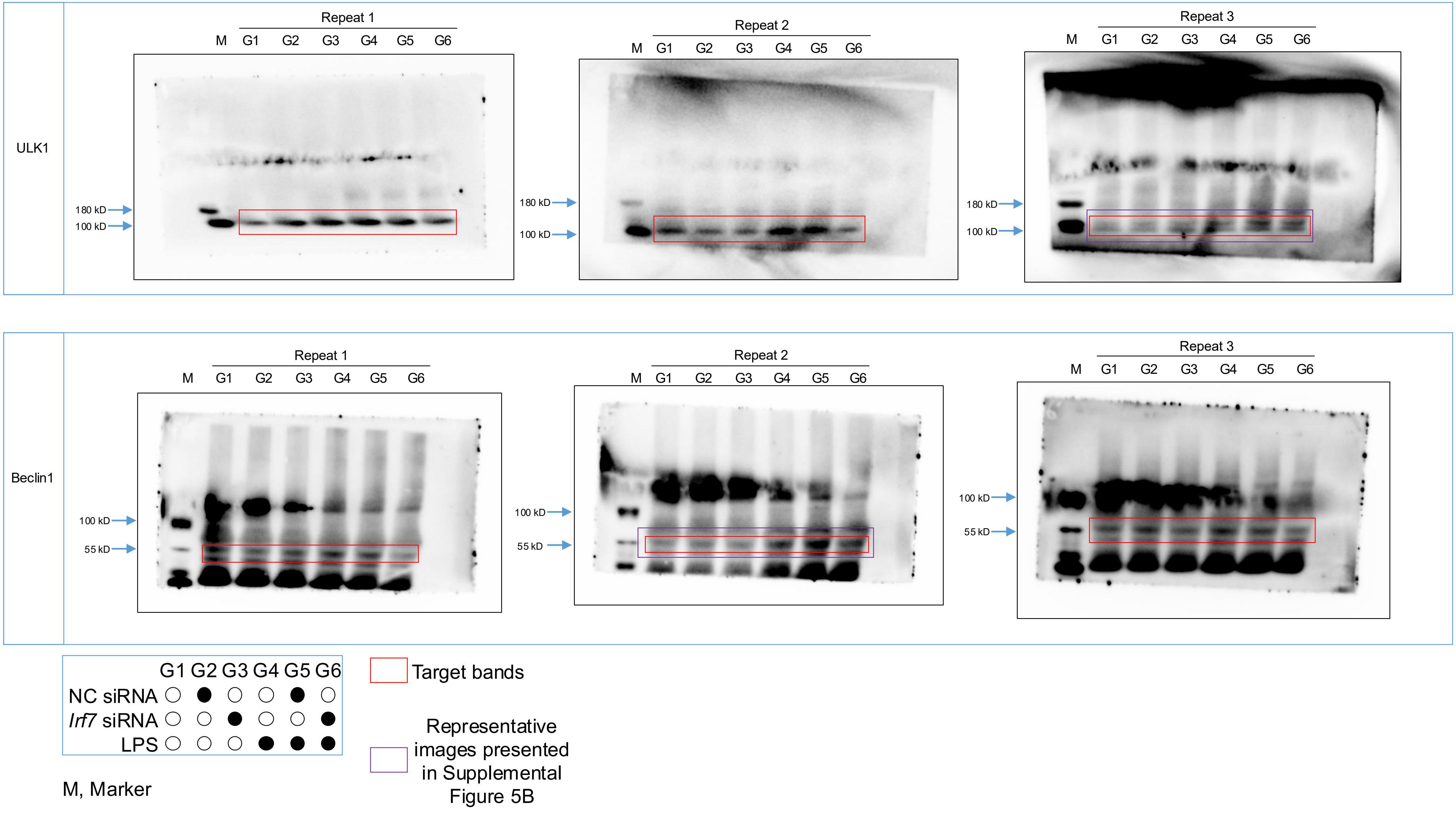

Unedited blot for Figure 4C and Supplemental Figure 5B

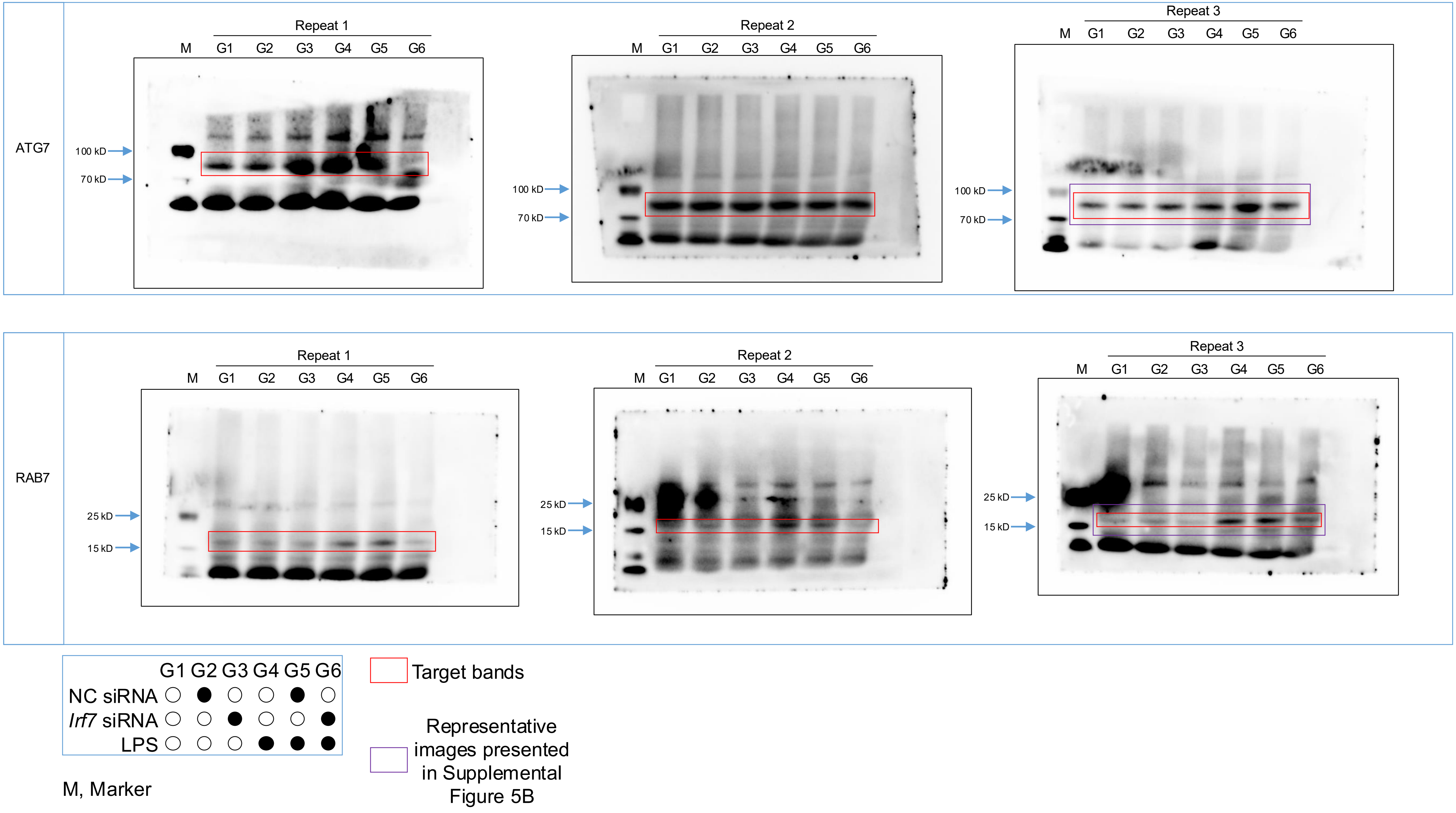

Unedited blot for Figure 4C and Supplemental Figure 5B

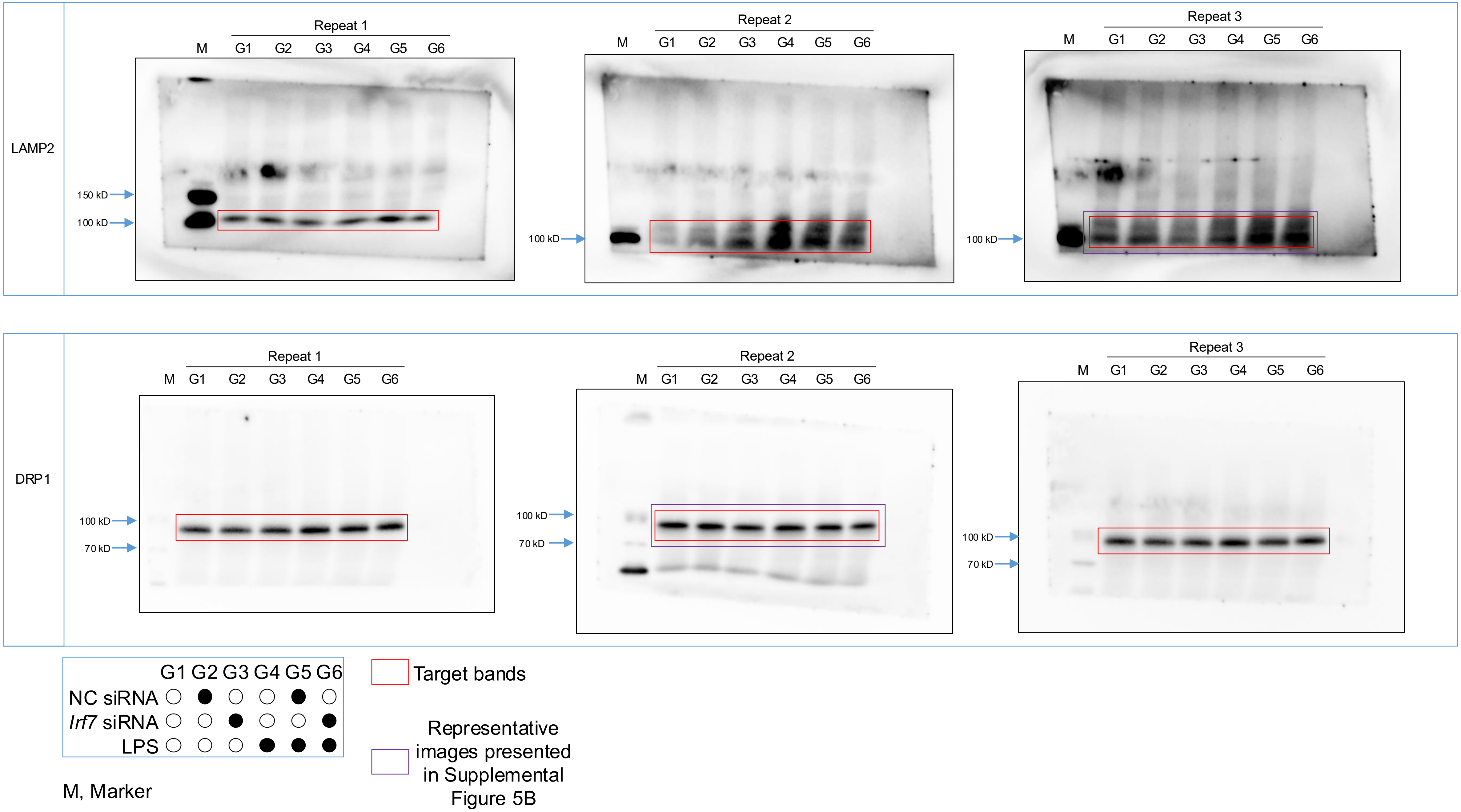

Unedited blot for Supplemental Figure 1

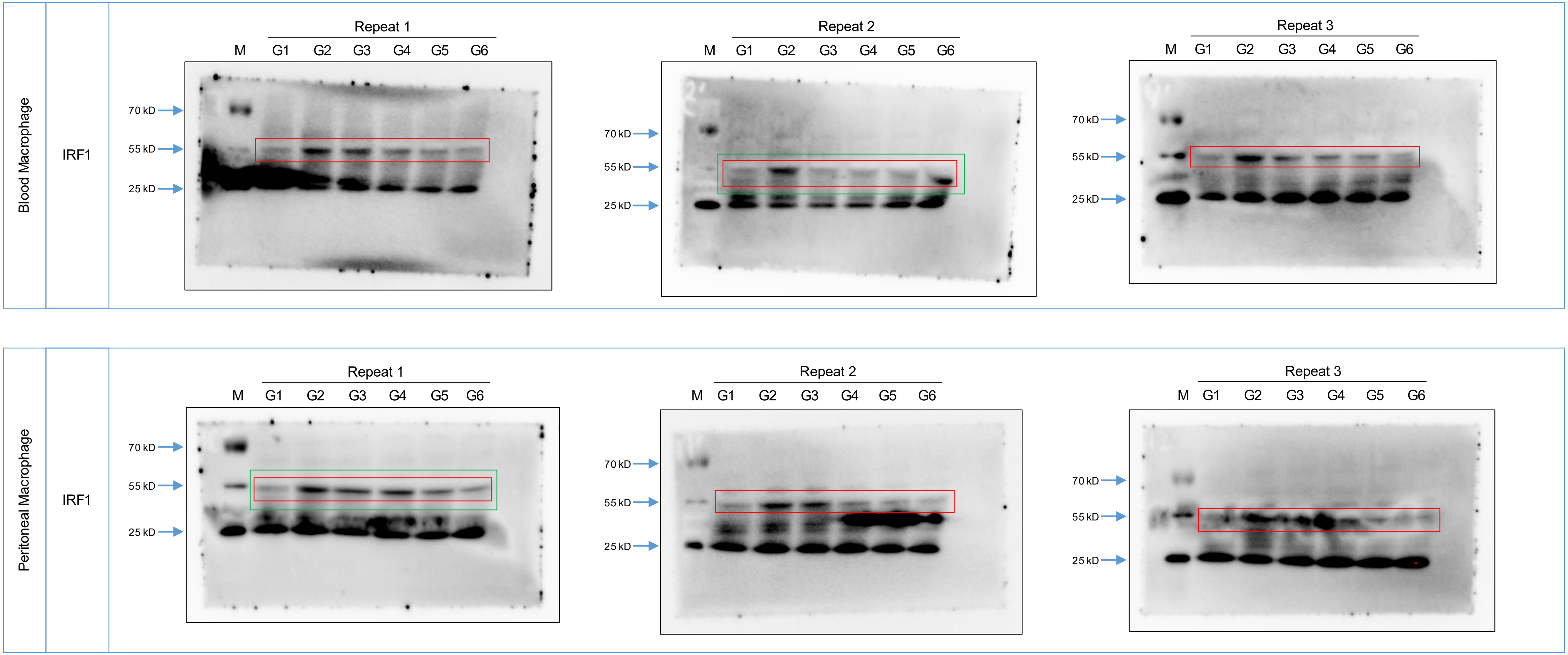

	G1	G2	G3	G4	G5	G6
CLP (h)	0	2	4	8	16	24

M, Marker

Target bands

Representative images presented in Supplemental Figure 1A

Unedited blot for Supplemental Figure 1

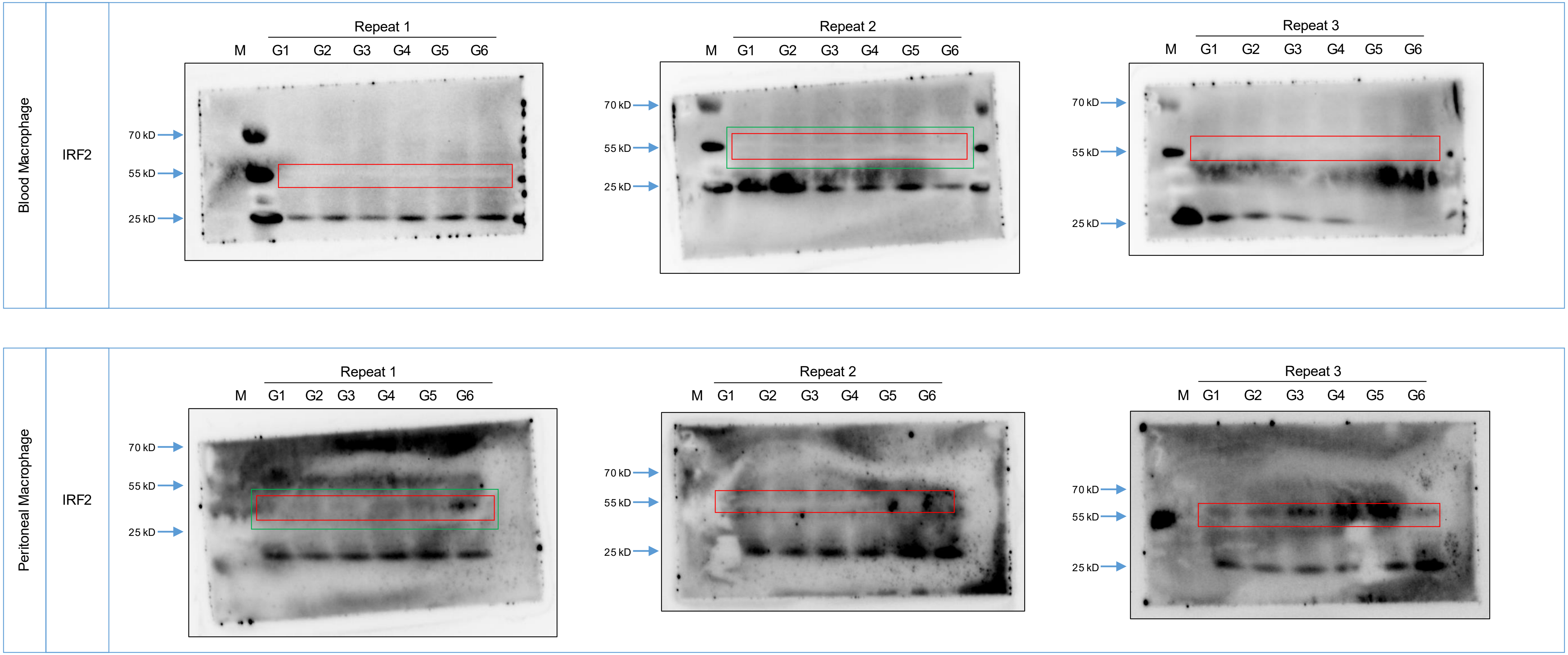

	G1	G2	G3	G4	G5	G6
CLP (h)	0	2	4	8	16	24

M, Marker

Target bands

Representative images presented in Supplemental Figure 1A

Unedited blot for Supplemental Figure 1

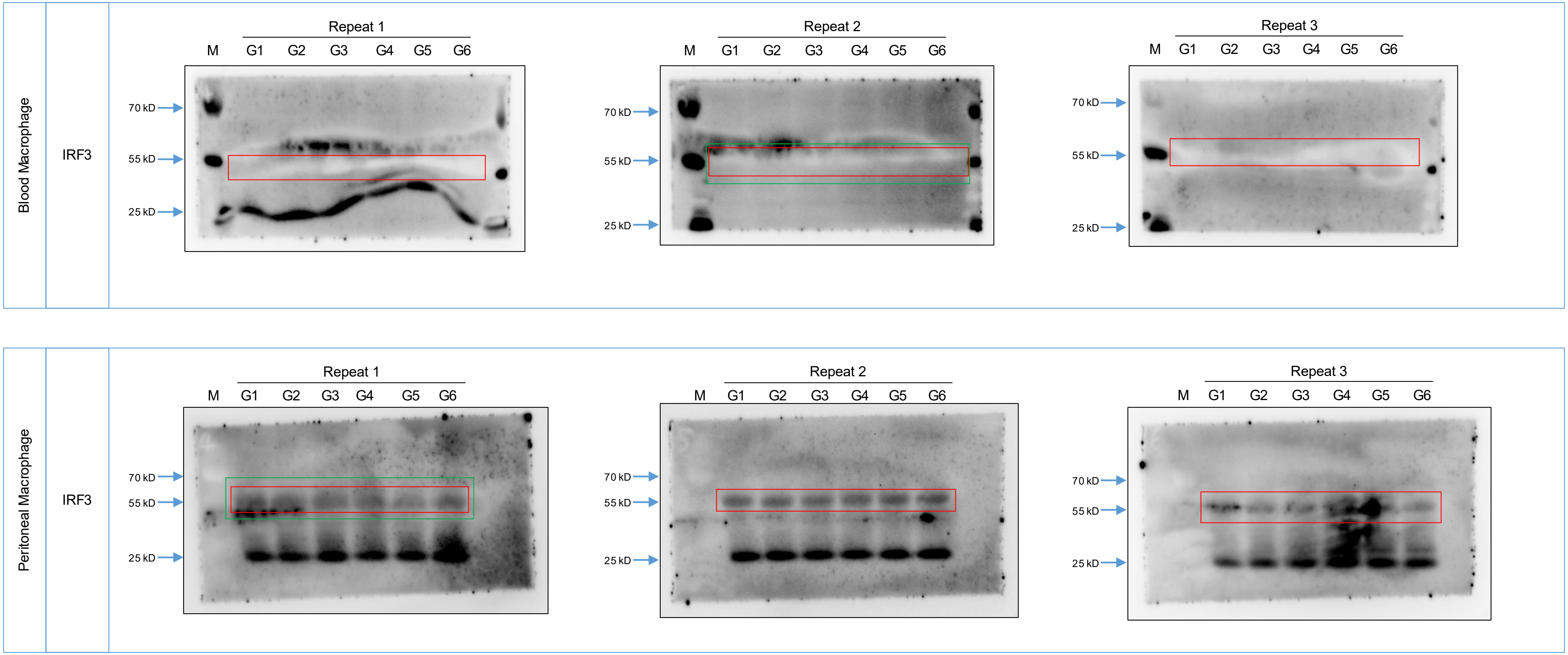

	G1	G2	G3	G4	G5	G6
CLP (h)	0	2	4	8	16	24

M, Marker

Target bands

Representative images presented in Supplemental Figure 1A

Unedited blot for Supplemental Figure 1

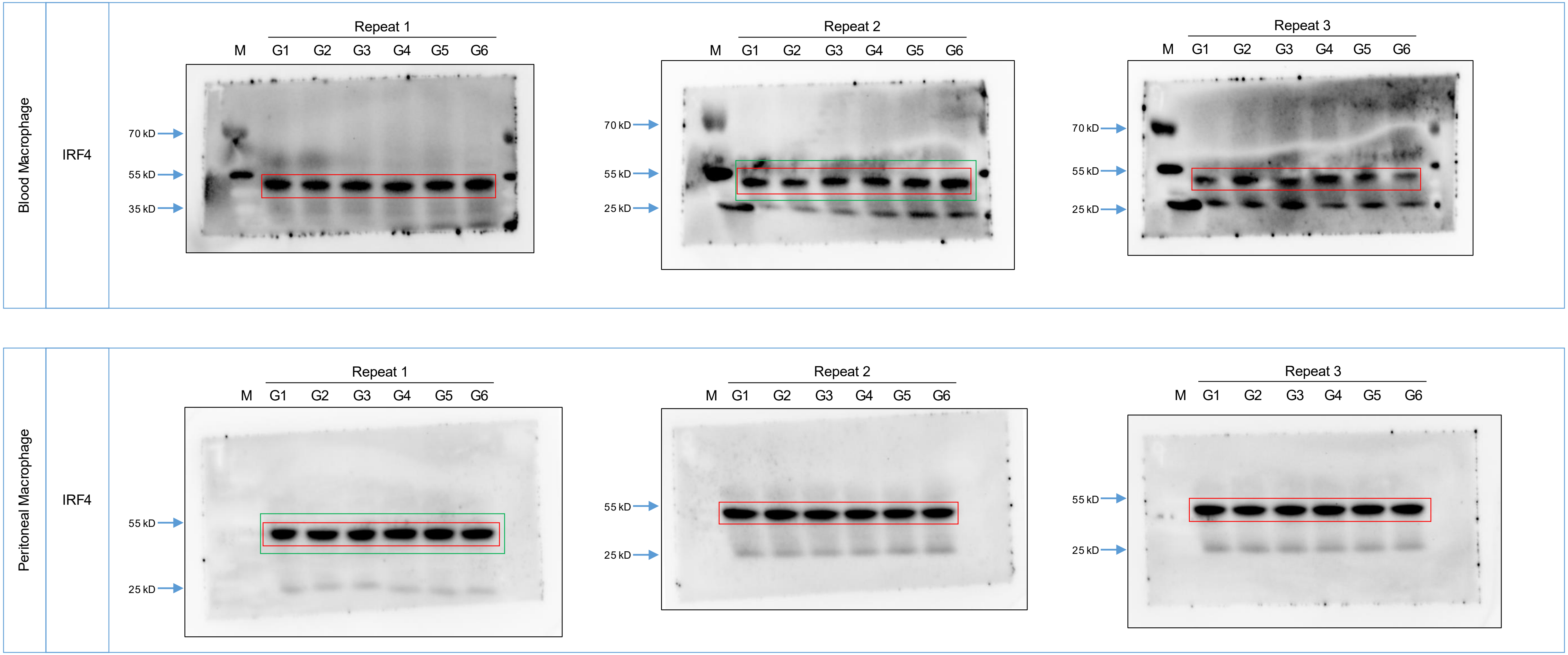

	G1	G2	G3	G4	G5	G6
CLP (h)	0	2	4	8	16	24

M, Marker

Target bands

Representative images presented in Supplemental Figure 1A

Unedited blot for Supplemental Figure 1

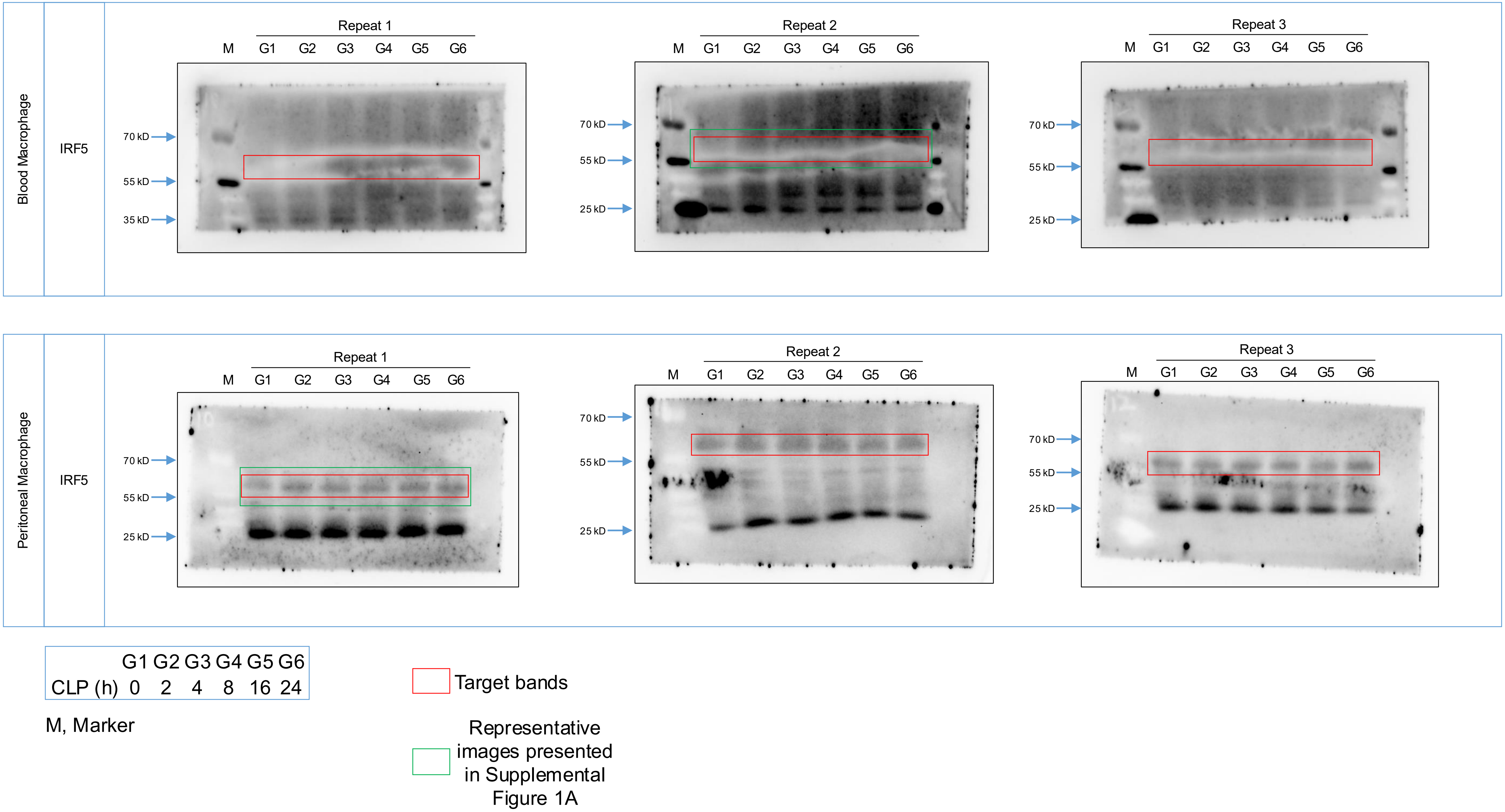

Unedited blot for Supplemental Figure 1

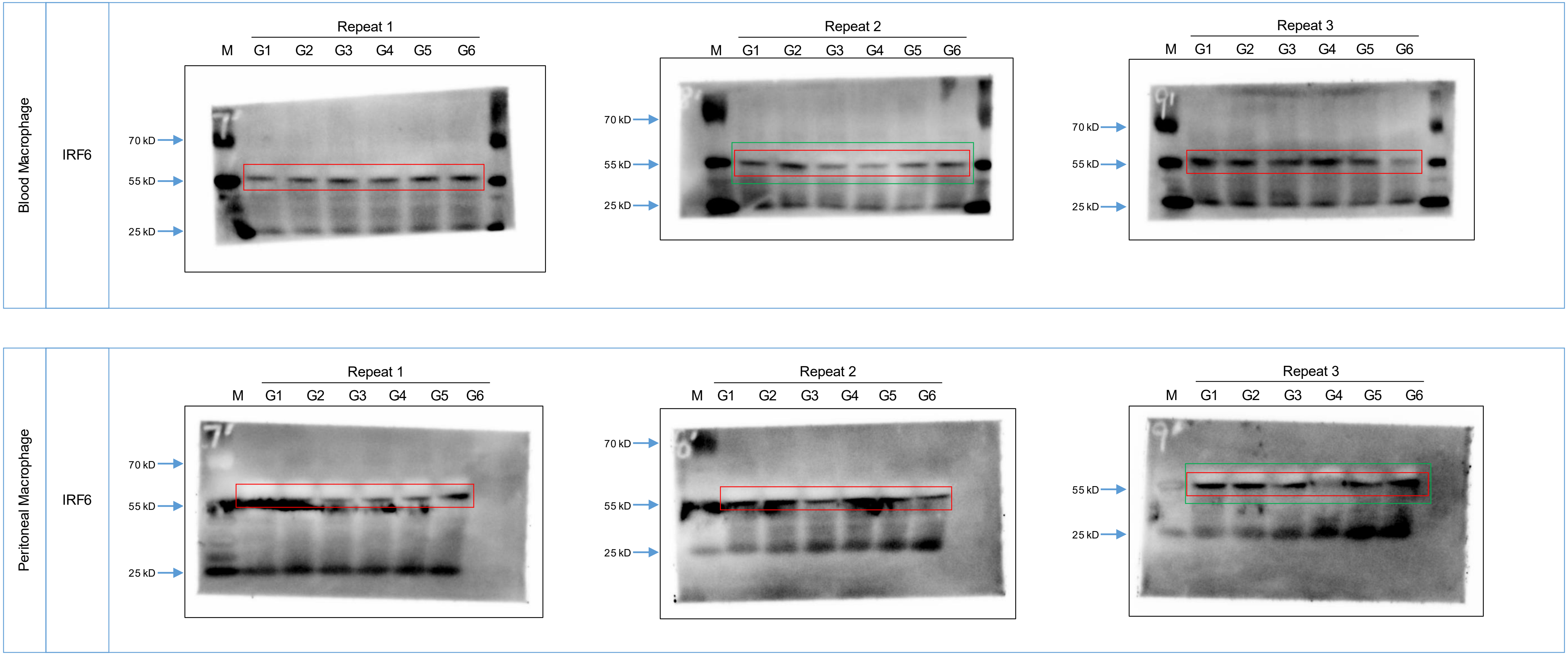

	G1	G2	G3	G4	G5	G6
CLP (h)	0	2	4	8	16	24

M, Marker

Target bands

Representative images presented in Supplemental Figure 1A

Unedited blot for Supplemental Figure 1

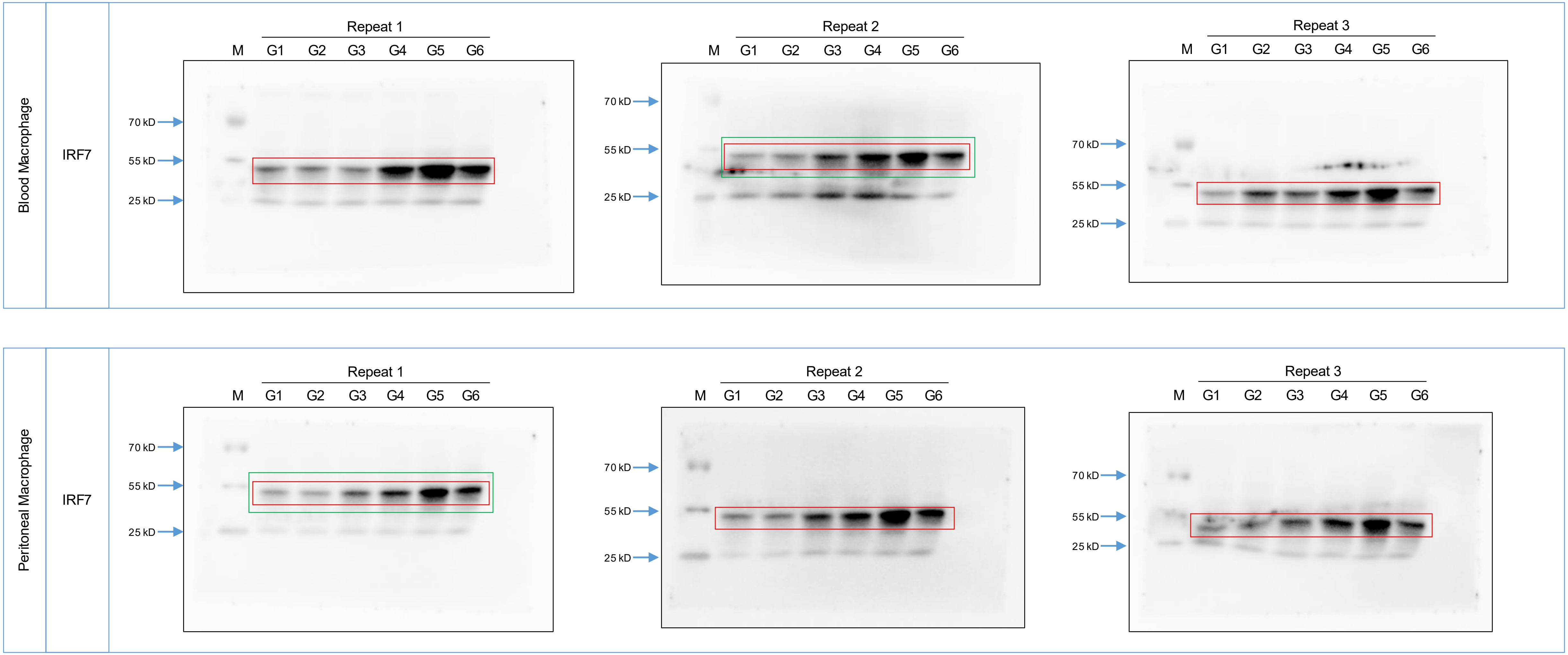

	G1	G2	G3	G4	G5	G6
CLP (h)	0	2	4	8	16	24

M, Marker

Target bands

Representative images presented in Supplemental Figure 1A

Unedited blot for Supplemental Figure 1

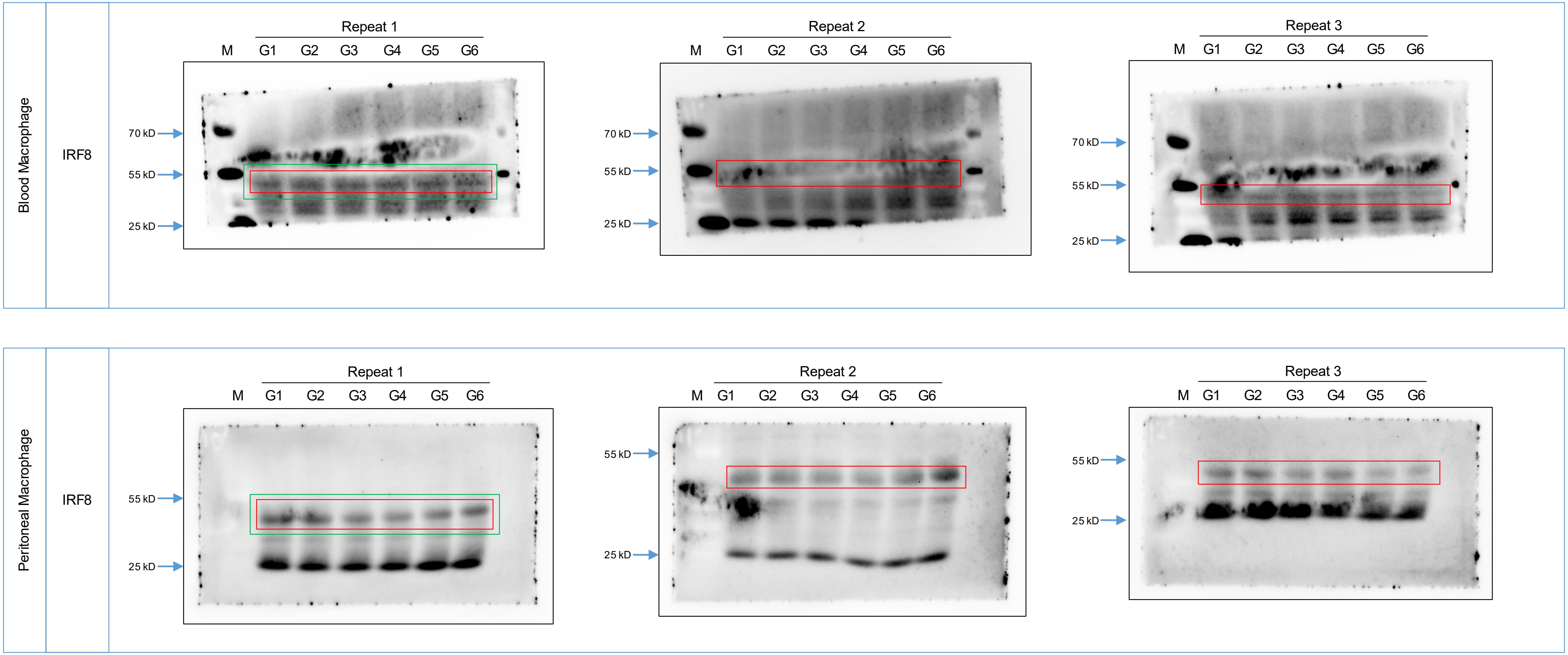

	G1	G2	G3	G4	G5	G6
CLP (h)	0	2	4	8	16	24

M, Marker

Target bands

Representative images presented in Supplemental Figure 1A

Unedited blot for Supplemental Figure 1

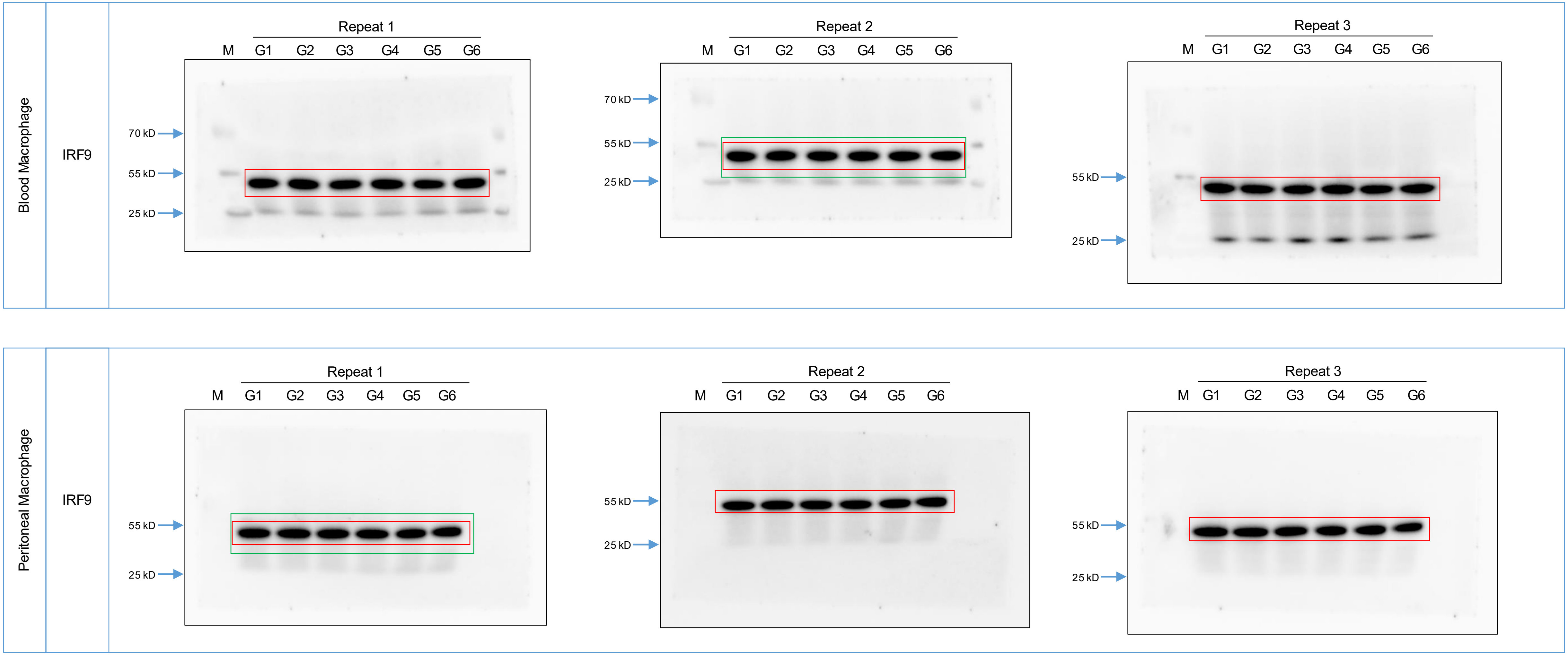

	G1	G2	G3	G4	G5	G6
CLP (h)	0	2	4	8	16	24

M, Marker

Target bands

Representative images presented in Supplemental Figure 1A

Unedited blot for Supplemental Figure 1

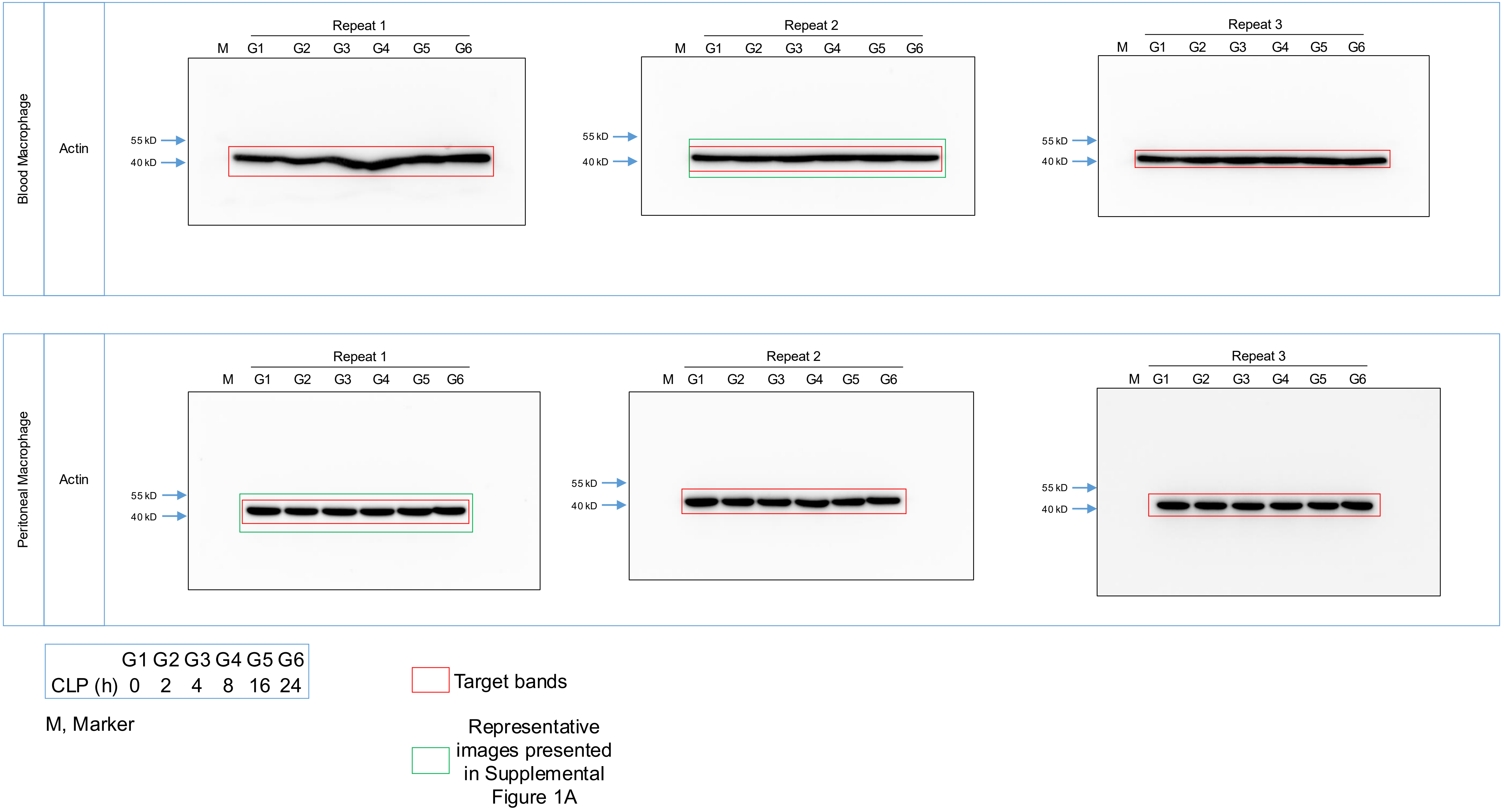

Unedited blot for Supplemental Figure 3A, B

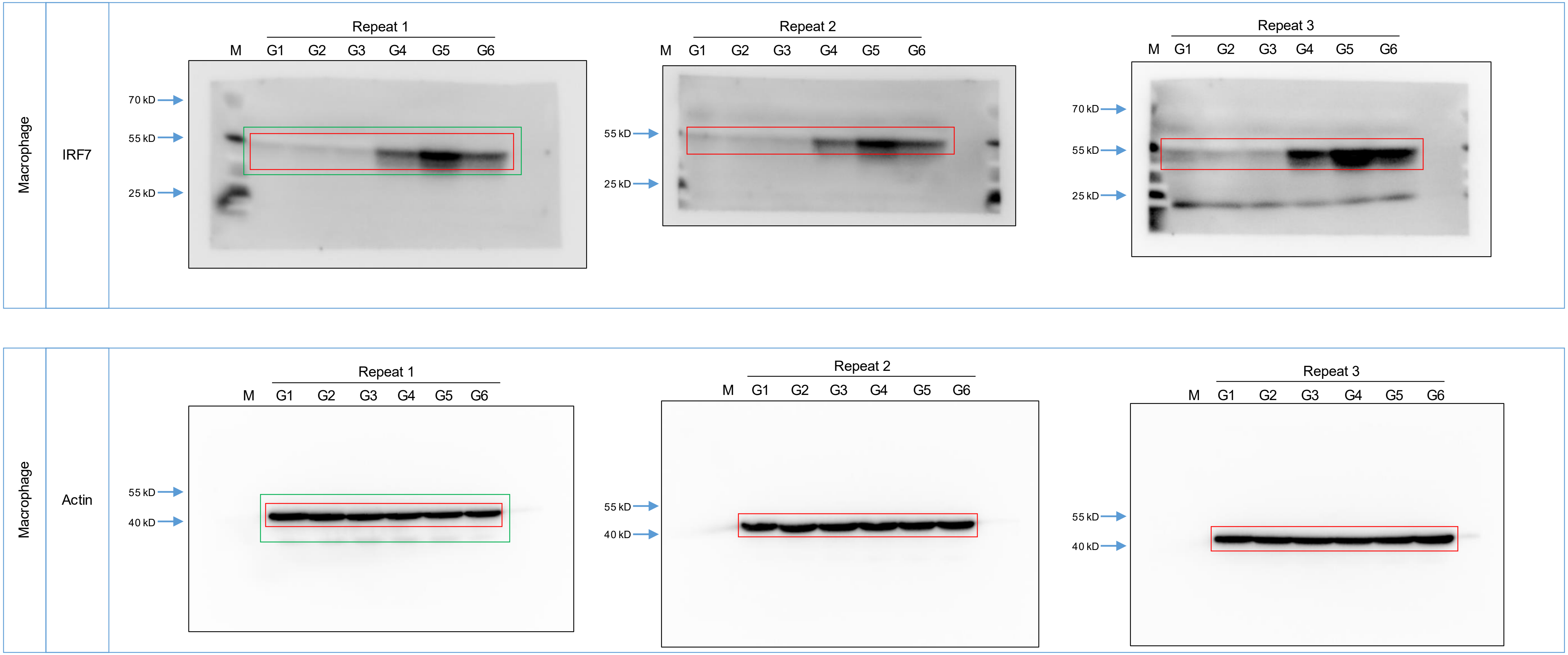

	G1	G2	G3	G4	G5	G6
CLP (h)	0	2	4	8	16	24

M, Marker

Target bands (red box)

Representative images presented in Supplemental Figure 3A (green box)

Unedited blot for Supplemental Figure 3A, B

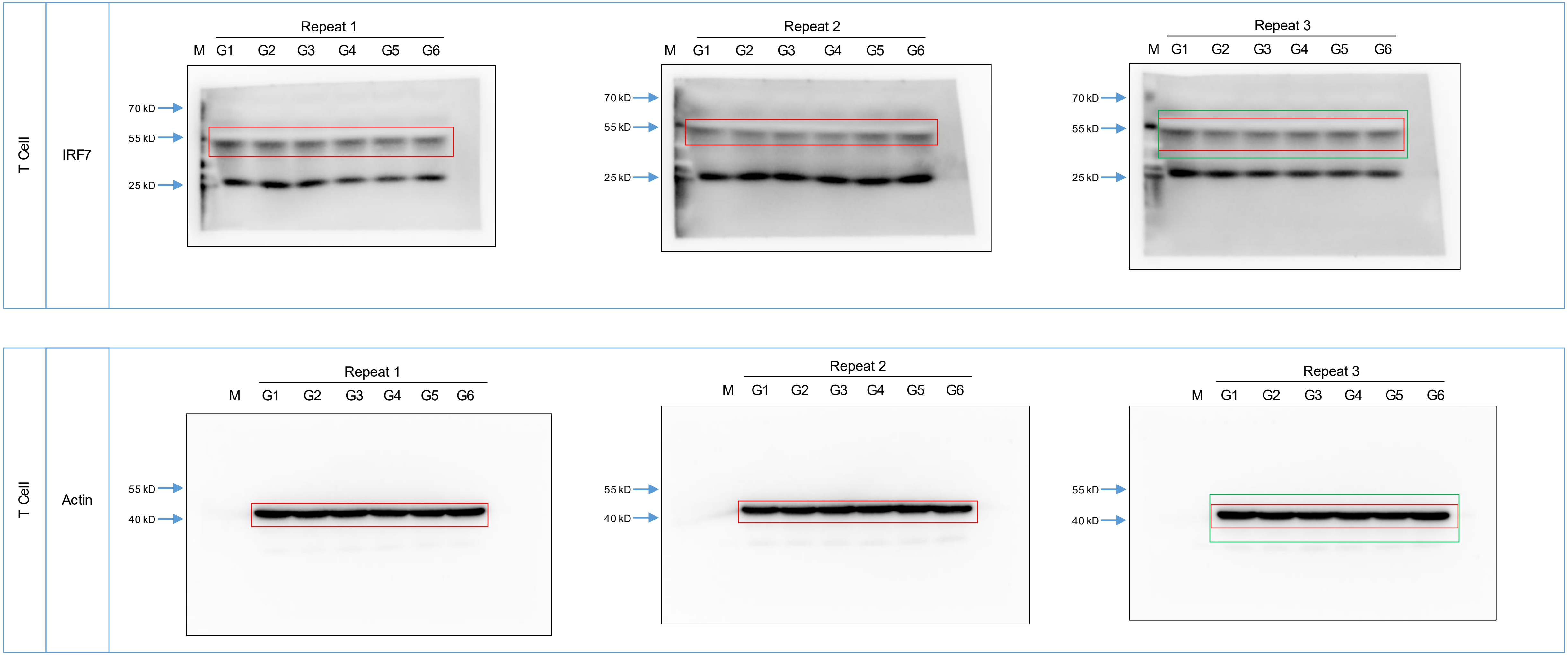

	G1	G2	G3	G4	G5	G6
CLP (h)	0	2	4	8	16	24

M, Marker

Target bands (red box)

Representative images presented in Supplemental Figure 3A (green box)

Unedited blot for Supplemental Figure 3A, B

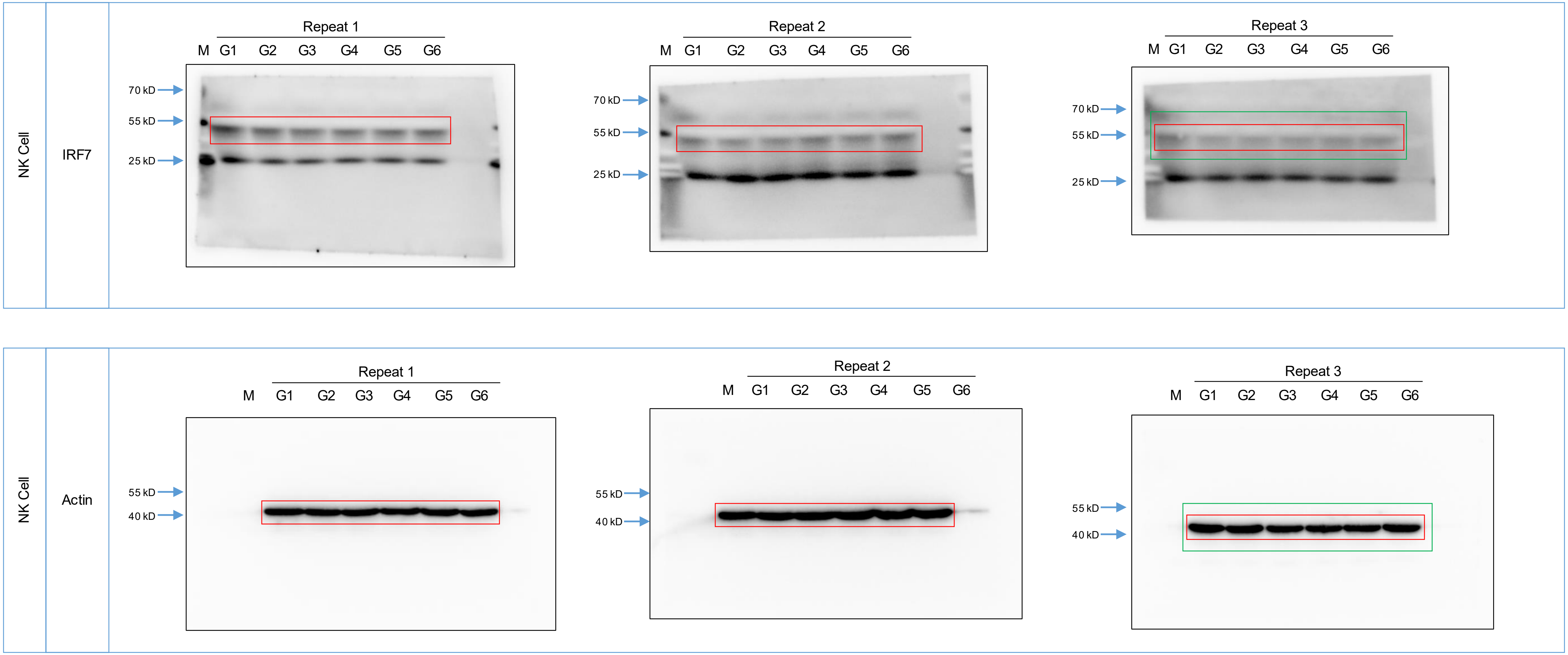

	G1	G2	G3	G4	G5	G6
CLP (h)	0	2	4	8	16	24

M, Marker

Target bands

Representative images presented in Supplemental Figure 3A

Unedited blot for Supplemental Figure 8A

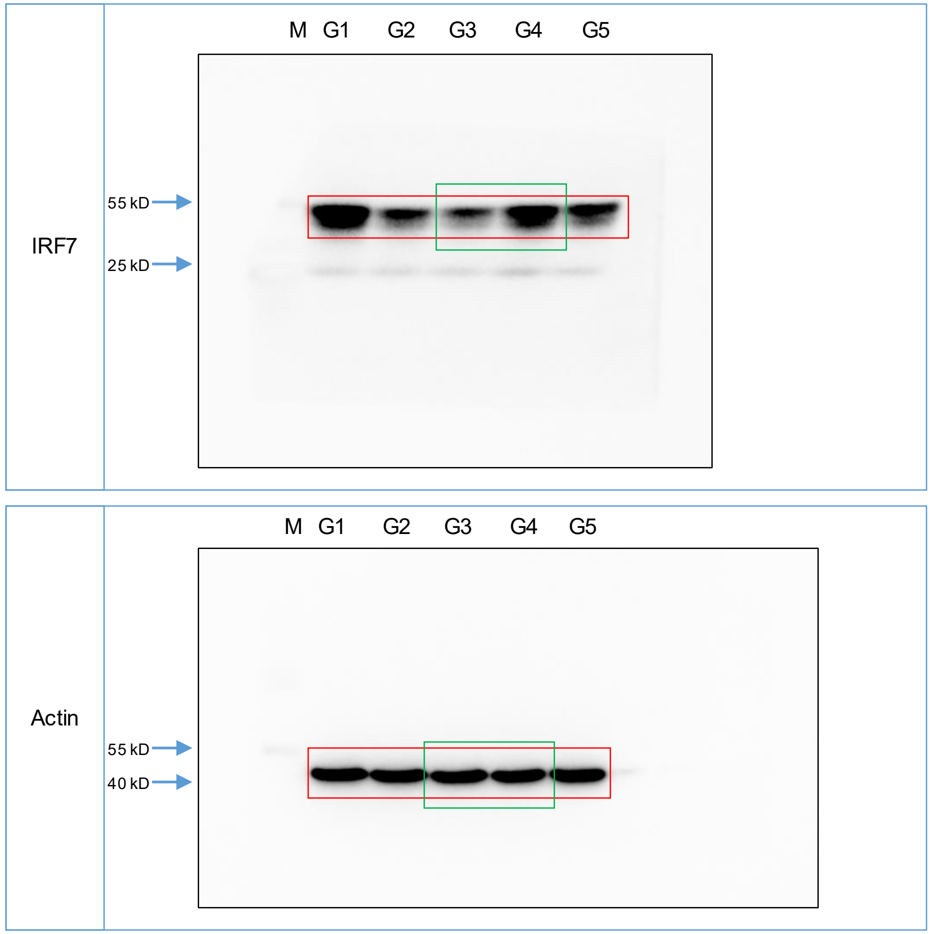

G1, Macrophages from WT mice underwent CLP for 16h
G2, BMDMs with normal culture
G3, BMDMs transfected with vector for 48h
G4, BMDMs transfected with *Irf7* gene for 48h
G5, BMDMs transfected with *Irf7* gene for 72h

M, Marker
Representative images presented in Supplemental Figure 8A
Target bands

Unedited blot for Supplemental Figure 8B

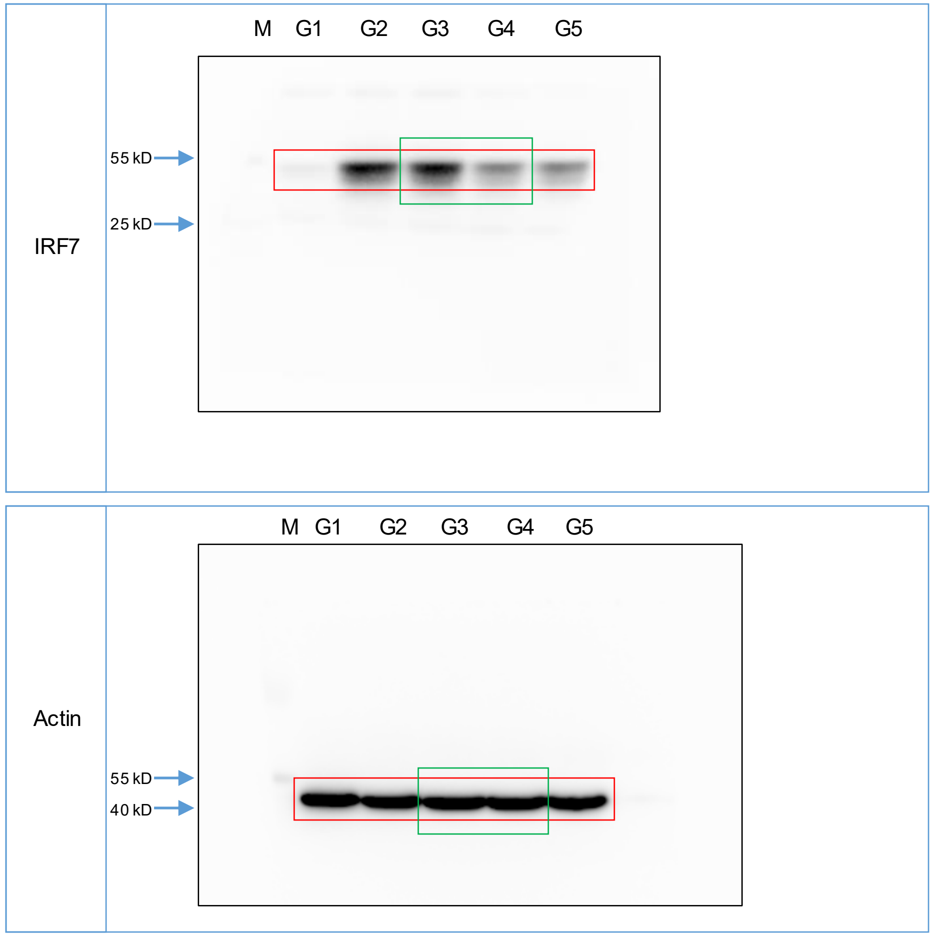

G1, Macrophages from WT mice underwent CLP for 0h
G2, BMDMs with normal culture
G3, BMDMs transfected with NC siRNA for 48h
G4, BMDMs transfected with *Irf7* siRNA for 48h
G5, BMDMs transfected with *Irf7* siRNA for 72h

M, Marker
Representative images presented in Supplemental Figure 8B
Target bands

Unedited blot for Supplemental Figure 13A

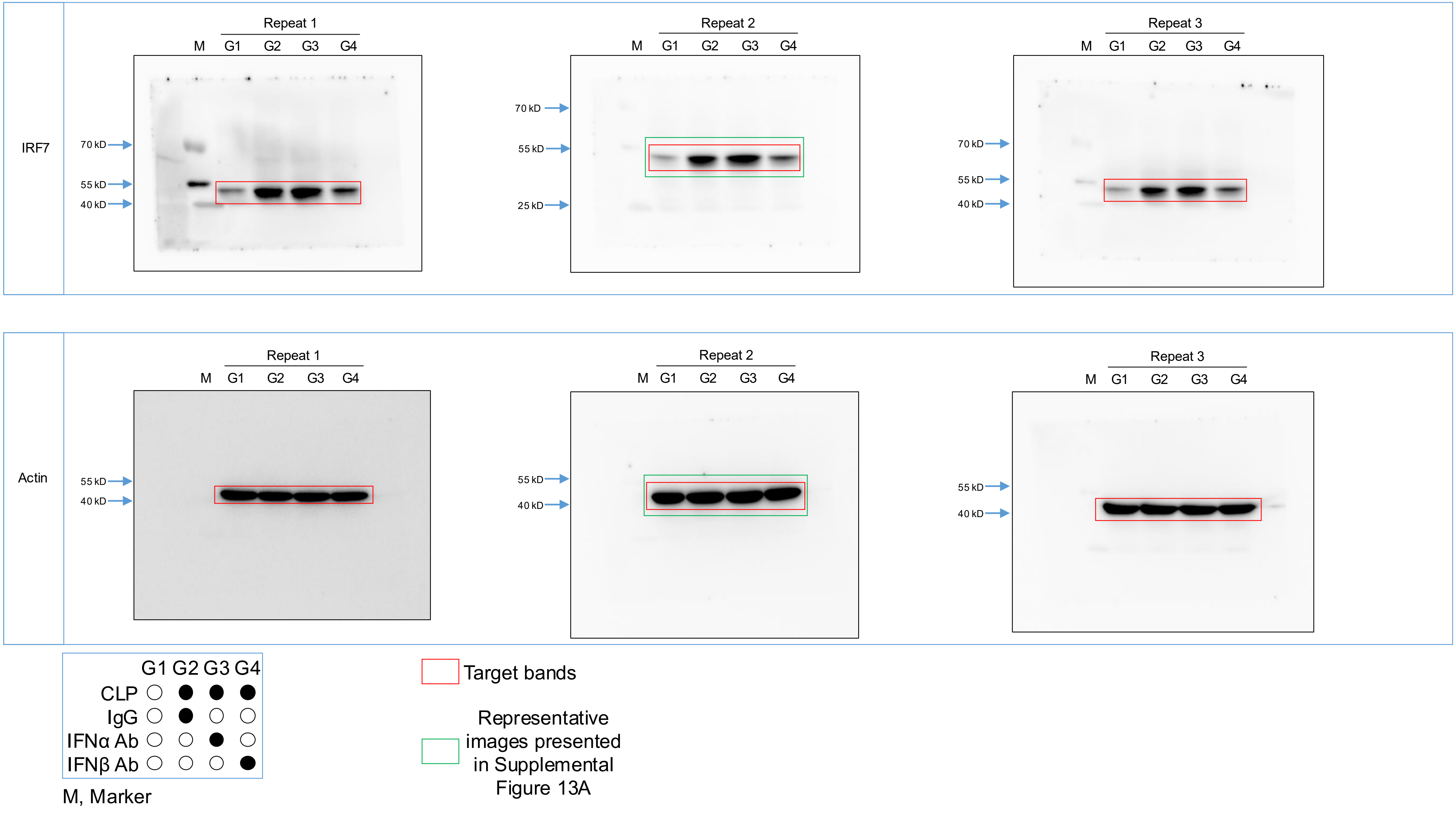

Unedited blot for Supplemental Figure 13B

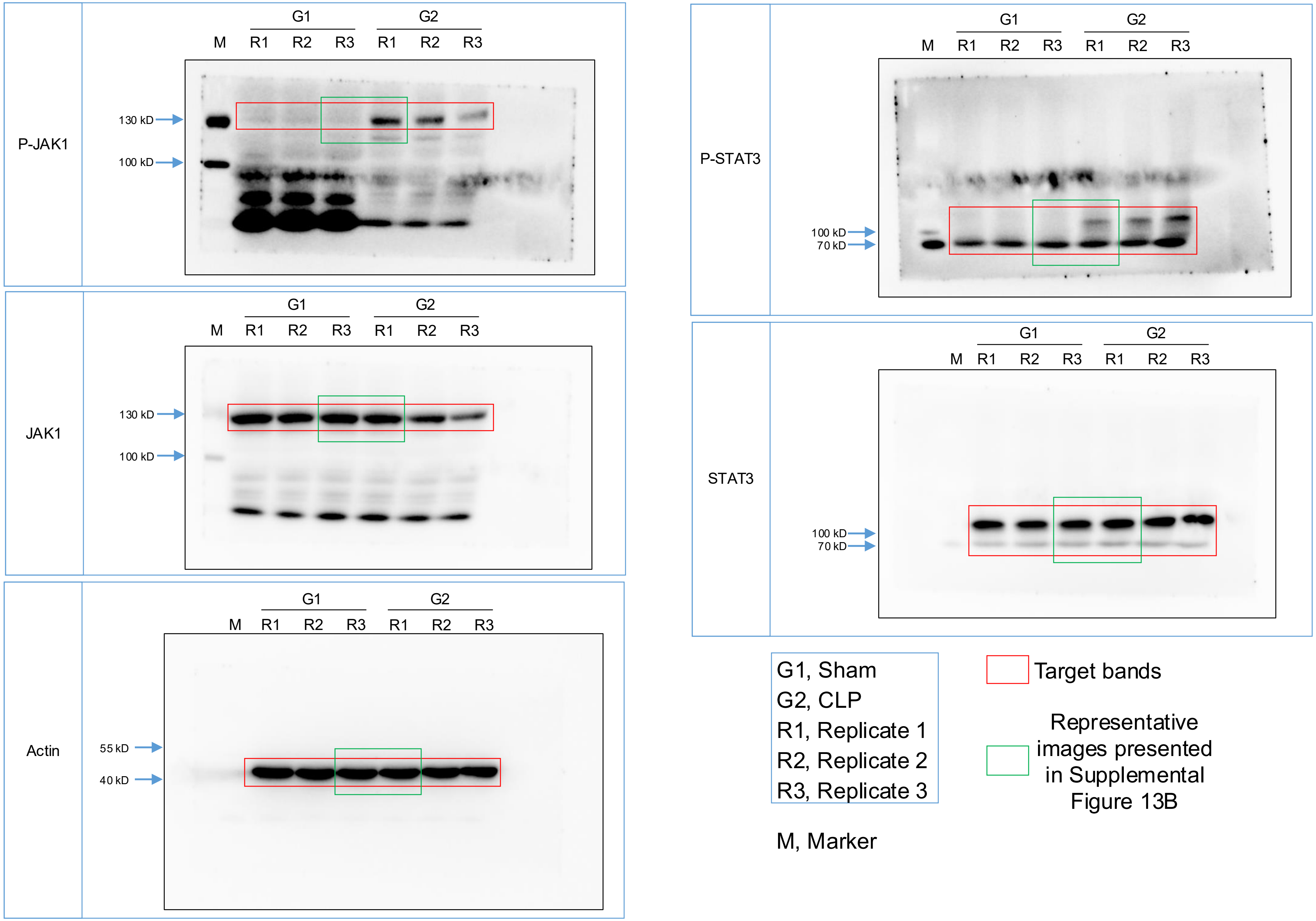

Unedited blot for Supplemental Figure 13C

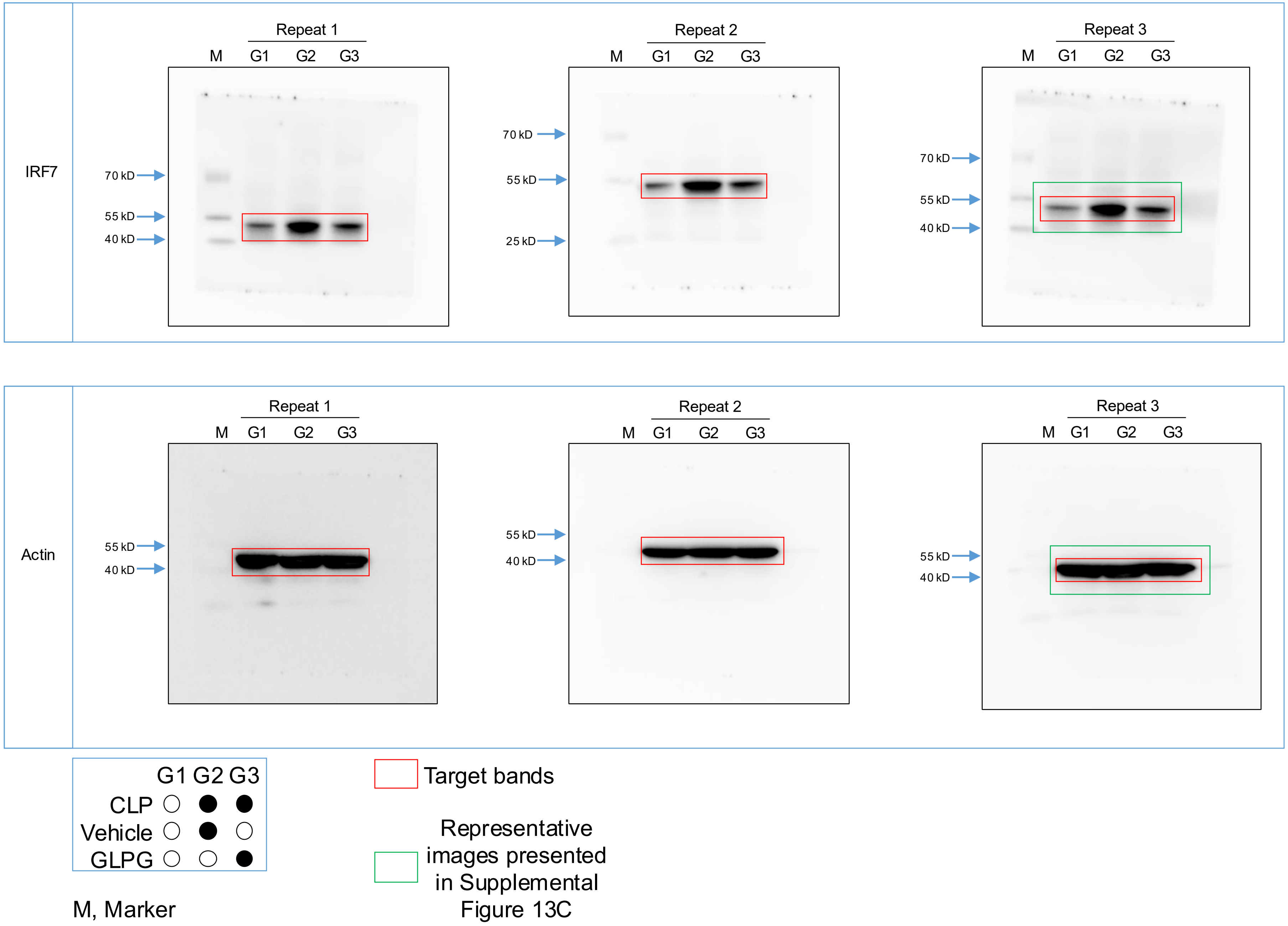

Unedited blot for Supplemental Figure 13D

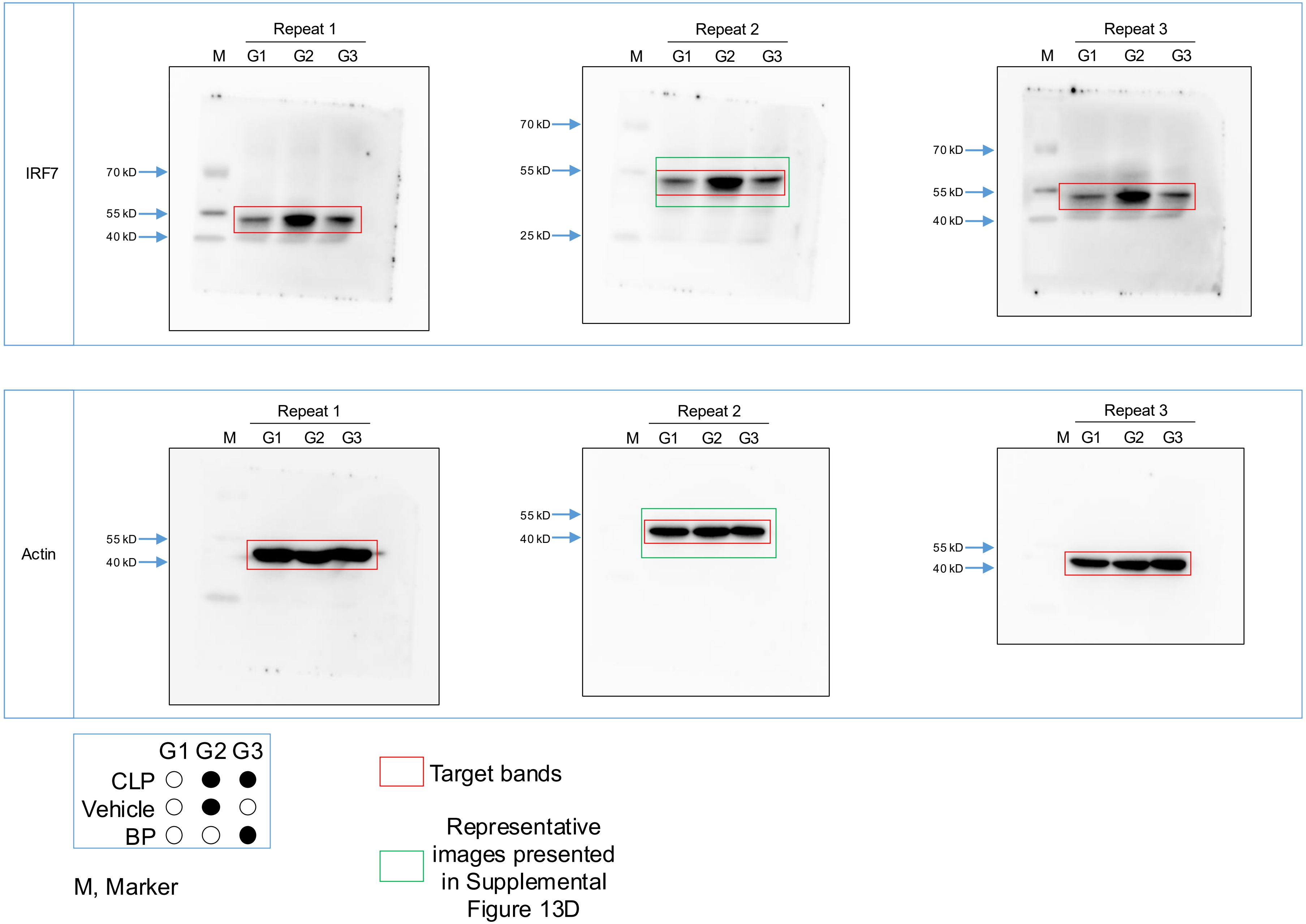

Supplement: Unedited blot and gel images [file jciinsight-10-189420-s009.pdf]
